# Fenugreek seed extract–doxorubicin synergy against hepatocellular carcinoma in HepG2 cells: in vitro and in silico mechanistic studies

**DOI:** 10.1186/s12906-026-05386-3

**Published:** 2026-05-06

**Authors:** Wesam Ragab, Kamel Mahmoud, Rasha M. Allam, Wesam S. Qayed, Osama M. Gomaa, Seham S. El-Hawary, Abeer S. Moawad, Rabab Mohammed

**Affiliations:** 1 Pharmacognosy Department, Faculty of Pharmacy, MUST University, 6th October City, Giza, 12566 Egypt; 2 Pharmacology Department, Medical and Clinical Research Institute, National Research Centre, 33 El-Bohouth St., Dokki, P.O.12622, Cairo, Egypt; 3 Medicinal Chemistry Department, Faculty of Pharmacy, Assuit University, Assuit, 71526 Egypt; 4 Pharmacognosy Department, Faculty of Pharmacy, Cairo University, Cairo, 11562 Egypt; 5 Natural Products Discovery Core, Life Sciences Institute, University of Michigan, Ann Arbor, MI 48109 USA; 6 Pharmacognosy Department, Faculty of Pharmacy, Beni-Suef University, Beni-Suef, 62514 Egypt

**Keywords:** Fenugreek seeds, *UHPLC-QTOF-MS/MS*, Trigonelline, Hepatocellular carcinoma (HCC), Synergistic cytotoxicity, CompuSyn

## Abstract

**Background:**

Hepatocellular carcinoma (HCC) is one of the most prevalent malignancies worldwide. The therapeutic efficacy of conventional chemotherapeutic agents such as doxorubicin (DOX) is limited by dose-dependent toxicity and the development of drug resistance. Combination strategies incorporating bioactive natural products may enhance anticancer efficacy while enabling dose reduction. The present study aimed to evaluate the potential synergistic cytotoxicity of combining Fenugreek aqueous extract (FAE) with (DOX) against the HepG2 cell line.

**Methods:**

Phytochemical characterization was performed using *UHPLC-QTOF-MS/MS Profiling* and HPLC. Cell viability and selectivity were assessed using the SRB assay. Apoptosis, necrosis, autophagy, and cell cycle distribution were analysed by flow cytometry and Western blotting. Drug–drug interaction was evaluated using the Chou–Talalay method. Molecular docking was performed to explore potential interactions between selected FAE constituents and apoptosis- and autophagy-related protein targets.

**Results:**

FAE enhanced DOX’s cytotoxicity on HepG2 cells, with the interaction ranging from synergistic to additive depending on the concentration ratio. The DOX/FAE combination enhanced cell death through sub-G1 arrest and augmented apoptotic, necrotic, and autophagic responses compared with monotherapies. Western blot analysis demonstrated modulation of the Bax/Bcl-2 ratio and increased LC3-II expression. Docking simulations suggested favourable binding of selected steroidal saponins to Bcl-2 and LC3 proteins.

**Conclusion:**

These findings indicate that FAE potentiates DOX-induced cytotoxicity in vitro through modulation of multiple regulated cell death pathways. While the results support the possibility of this combination as a dose-modulating strategy, further validation in additional HCC models and in vivo systems is required.

**Graphical abstract:**

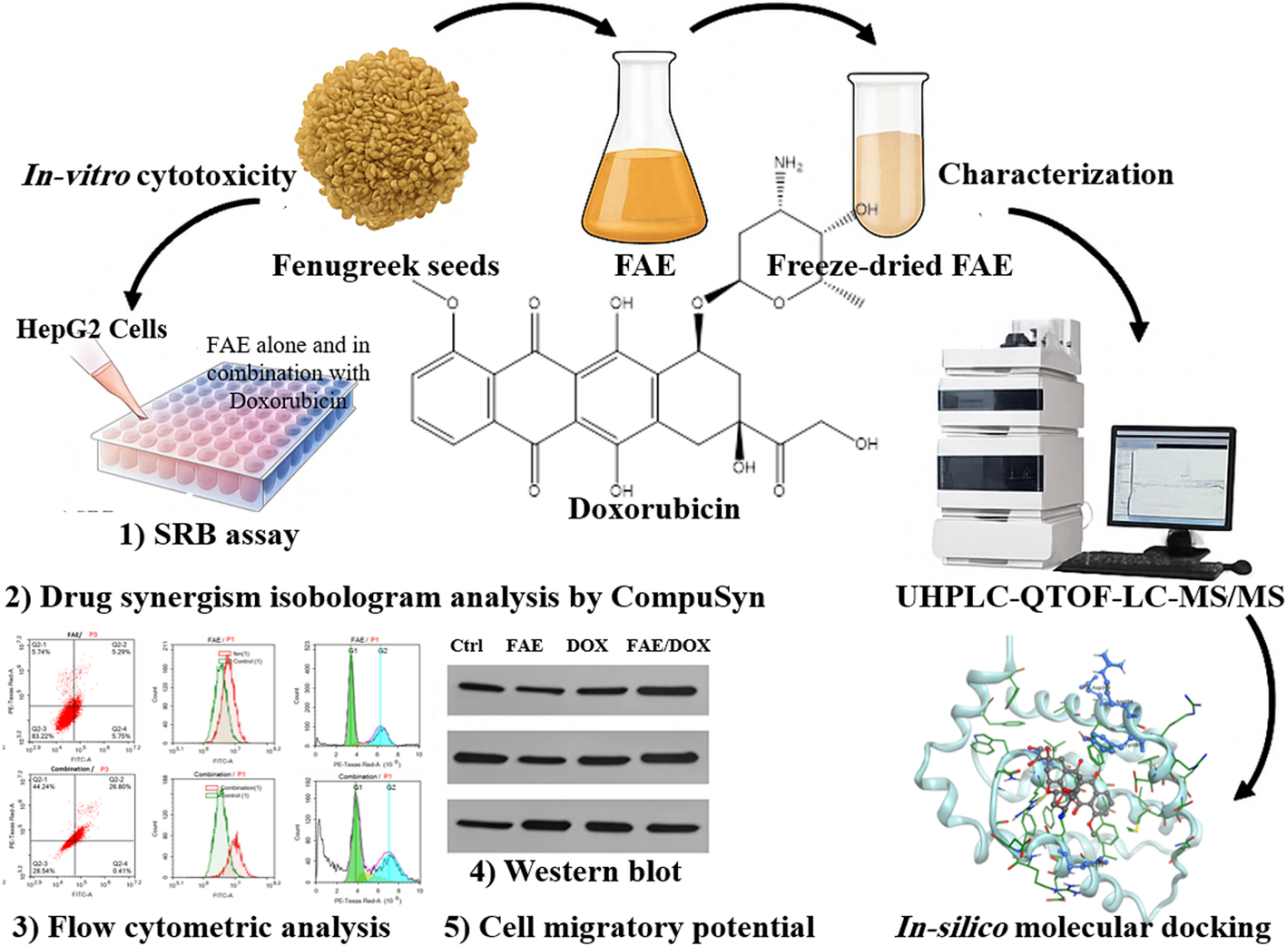

**Supplementary Information:**

The online version contains supplementary material available at 10.1186/s12906-026-05386-3.

## Introduction

Liver cancer remains one of the most prevalent malignancies and the leading cause of cancer-related mortality globally, with hepatocellular carcinoma (HCC) accounting for approximately 80–90% of cases [1, 2]. Despite advances in therapeutic approaches, the prognosis for HCC patients remains poor, primarily due to late diagnosis, high recurrence rates, and limited treatment efficacy [3, 4].

Conventional chemotherapeutics, such as doxorubicin, remain a cornerstone in HCC management, whether used alone or in combination; however, their clinical efficacy is limited by severe dose-dependent toxicities and the development of drug resistance [5, 6]. These challenges underscore the crucial need for innovative therapeutic approaches to achieve improved clinical outcomes in HCC [7].

Natural products represent a promising source of novel therapies, offering multitarget benefits with potentially fewer side effects, which is extremely valuable in HCC treatment [8, 9]. Fenugreek (*Trigonella foenum-graecum* L.), an annual legume of the Fabaceae family cultivated worldwide and historically used as a medicinal herb, has been extensively studied for its diverse pharmacological activities, which include antioxidant, anti-inflammatory, antilipidemic, anticancer, antiulcerogenic, antipyretic, antibacterial, and antifungal properties [10, 11]. Notably, fenugreek extracts and their bioactive constituents have been reported to modulate multiple hallmarks of cancer, including proliferation, inflammation, angiogenesis, invasion, and metastasis [12].

Fenugreek has attracted considerable attention for its anticancer properties, particularly against HCC. Several studies have demonstrated that fenugreek extracts and their bioactive constituents exert cytotoxic and chemosensitizing effects in liver cancer models. Diosgenin, a major steroidal saponin, has been reported to inhibit STAT3 signaling, suppress proliferation, and enhance chemosensitivity in HCC cells [13]. Additionally, fenugreek seed extracts have shown selective cytotoxic activity and proteomic modulation in cancer cells [14], while apoptosis induction in HepG2 cells has been associated with upregulation of p53 and proliferating cell nuclear antigen [15]. A recent comprehensive review further highlighted the molecular targets, potential benefits, and safety considerations of fenugreek administration in HCC [16].

Furthermore, the integration of computational techniques—such as virtual screening, molecular dynamics simulations, pharmacophore modeling, network biology, and machine learning—has revolutionized the drug discovery process by enhancing efficiency, reducing time, and lowering costs. Among these approaches, structure-based drug design is particularly valuable for predicting ligand–protein interactions through molecular docking simulations, enabling the evaluation of binding affinity and the identification of optimal binding conformations within predefined active sites [17].

Previous studies exploring fenugreek cytotoxic activity have primarily focused on isolated compounds, organic solvent extracts, or single mechanistic endpoints, with limited integration of phytochemical profiling, synergy analysis, and mechanistic validation [18–20].

In contrast, the present study was designed to evaluate, in vitro, the potential synergistic antitumor activity of the crude aqueous extract of Fenugreek seeds in combination with doxorubicin against the HepG2 human hepatoblastoma cell line, as a well-characterized in vitro model of HCC, widely used in mechanistic and chemosensitization studies due to its stable phenotype and functional p53 status [21]. Furthermore, an in silico investigation was performed to elucidate the molecular mechanisms underlying the observed synergistic effect. This combined therapeutic approach aimed to reduce the cytotoxic dose of doxorubicin while maintaining its antitumor efficacy, thereby potentially minimizing the adverse effects associated with conventional chemotherapy.

## Materials and methods

### Plant material and extraction procedure

Fenugreek seeds (*Trigonella foenum-graecum* L.) were kindly provided by Harraz farm in Nubaria and authenticated by Dr. Ibrahim Elgarf, Professor of Taxonomy at Cairo University, Egypt. A voucher specimen (No. ID- BUPD-134) was deposited in the Herbarium, Faculty of Pharmacy, Beni-Suef University, Egypt. The seeds were reduced to a mesh number. 36 powder, and kept in a dim, tightly closed container for further processing. Fenugreek aqueous extract (FAE) was prepared by boiling dried plant powder (20 g) in 100 ml of distilled water (1:5 *w/v*) three times. Subsequently, the resulting supernatants were filtered; the collected filtrates were then concentrated and lyophilized, yielding 3 g of a yellow residue.

### *UHPLC-QTOF-MS/MS* profiling of fenugreek crude extract

Qualitative analysis for fenugreek extract was conducted by Agilent LC–MS system (Natural Products Discovery Core, Life Sciences Institute, University of Michigan, Ann Arbor, MI 48109, USA) composed of an Agilent 1290 Infinity II UHPLC coupled to an Agilent 6545 ESI-Q-TOF-MS in both negative and positive modes, aliquots (1 µL) of extract (1 mg/ml in MeOH) were analyzed on a Kinetex phenyl-hexyl (1.7 μm, 2.1_ 50 mm) column eluted with 1 min isocratic elution of 90% A (A: 100% H_2_O + 0.1% formic acid) followed by 6 min linear gradient elution to 100% B (95% MeCN + 5% H_2_O + 0.1% formic acid) with a flow rate of 0.4 ml/min. ESI conditions were set with a capillary temperature of 320 °C, a source voltage of 3.5 kV, and a sheath gas flow rate of 11 L/min. Ions detected in the full scan at an intensity above 1000 counts at 6 scans/s, with an isolation width of 1.3 ~ *m/z*, a maximum of 9 selected precursors per cycle, and using ramped collision energy (5 x *m/z*/100 + 10 eV). Purine C_5_H_4_N_4_ [M + H] + ion (*m/z* 121.050873) and hexakis (1 H,1 H,3 H-tetrafluoropropoxy)-phosphazene C_18_H_18_F_24_N_3_O_6_P_3_ [M + H] + ion (*m/z* 922.009798) were used as internal lock masses for positive mode while TFA C_2_HF_3_O_2_ [M - H]- ion (*m/z* 112.985587) and hexakis (1 H,1 H,3 H-tetrafluoropropoxy)-phosphazene C_18_H_18_F_24_N_3_O_6_P_3_ [M + TFA-H]- ion (*m/z* 1033.988109) were used as internal lock masses for negative mode. The acquired MS/MS data were converted from Agilent MassHunter (.d) files to the mzXML format using MSConvert [22].

### Quantitative analysis using HPLC

Trigonelline hydrochloride standard (Sigma-Aldrich ˗ St. Louis, MO, USA) and fenugreek aqueous extract (FAE) were analysed using a Waters 2690 Alliance HPLC system equipped with a Waters 996 photodiode array detector and Inertsil ODS Column (4.6 × 150 mm x 5 μm) at Nawah Scientific Centre, Mokattam, Cairo, Egypt.

#### Quantification of trigonelline HCl in fenugreek aqueous extract

Trigonelline HCl was quantified in FAE according to the modified method developed by PD, Hamrapurkar, and M. Faria 2008 [23]. A mixture of 0.1% Orthophosphoric acid: acetonitrile was used at a flow rate of 1 ml/min; detection was done at 255 nm, and the injection volume was 10 µl. A serial dilution of the standard stock solution was completed, and a calibration curve was established.

### Preparation of drug solutions and treatment protocol

FAE and DOX were dissolved in DMSO (Sigma-Aldrich). Working concentrations of FAE (31.25, 62.5, 125, 250, and 500 µg/ml) and DOX (0.01, 0.1, 1, 10, and 100 µg/ml) were freshly prepared before experiment by serial dilution in DMEM culture medium. For combination experiments, both agents were added simultaneously to the cells to ensure consistent exposure conditions. The final solvent concentration did not exceed 0.1% (*v/v*) in any experimental condition, a level confirmed to have no measurable effect on cell viability.

### Cell culture

HepG2, a human hepatoblastoma cancer cell line, and BNL CL2, Mouse normal liver cells, were obtained from Nawah Scientific Centre (Mokattam, Cairo, Egypt). Cells were maintained in Dulbecco’s Modified Eagle Medium (DMEM) media supplemented with 100 µg/ml of streptomycin, 100 units/ml of penicillin, and 10% of heat-inactivated fetal bovine serum (FBS) in a humidified, 5% (*v/v*) CO_2_ atmosphere at 37 °C [24].

### In vitro cell viability assay (SRB Assay)

The cell viability was determined using the Sulforhodamine B (SRB) method at Nawah Scientific Centre, Mokattam, Cairo, Egypt. Cells (5 × 10³/well) were seeded in 96-well plates, pre-incubated for 24 h, and treated with serial concentrations of Fenugreek aqueous extract (FAE) and Doxorubicin (DOX) for 72 h. After fixation with 10% Trichloroacetic acid (TCA; 1 h, 4 °C), cells were stained with 0.4% SRB (10 min), washed with 1% acetic acid, and air-dried. Protein-bound SRB dye was solubilized in 10 mM TRIS base, and absorbance was measured at 540 nm with a plate reader (BMG LABTECH; Germany). Cytotoxicity was evaluated as a percentage reduction in viability compared to untreated controls. Half maximal inhibitory concentrations (IC_50_) values were calculated for each experiment using GraphPad Prism Version 9.3.1. IC_50_ values were reported as mean ± SD [25].

The selectivity, or preferential cytotoxicity, of FAE and DOX towards cancer cells compared to normal cells was evaluated using the Selectivity Index (SI).

The SI values were calculated following the equation:

$$\:\mathrm{S}\mathrm{I}=\frac{\mathrm{I}\mathrm{C}50\:\mathrm{f}\mathrm{o}\mathrm{r}\:\mathrm{B}\mathrm{N}\mathrm{L}\:\mathrm{C}\mathrm{L}2\:\left(\mathrm{n}\mathrm{o}\mathrm{r}\mathrm{m}\mathrm{a}\mathrm{l}\:\mathrm{l}\mathrm{i}\mathrm{v}\mathrm{e}\mathrm{r}\:\mathrm{c}\mathrm{e}\mathrm{l}\mathrm{l}\:\mathrm{l}\mathrm{i}\mathrm{n}\mathrm{e}\right)\:}{\:\mathrm{I}\mathrm{C}50\:\mathrm{f}\mathrm{o}\mathrm{r}\:\mathrm{H}\mathrm{e}\mathrm{p}\mathrm{G}2\:\mathrm{c}\mathrm{e}\mathrm{l}\mathrm{l}\:\mathrm{l}\mathrm{i}\mathrm{n}\mathrm{e}}$$

An SI value greater than 2 indicates a high degree of selectivity against cancer cells, whereas an SI value less than 2 demonstrates nonspecific cytotoxicity and potential toxicity to normal cells [26].

### Drug synergism evaluation and selection of combination concentrations

The synergistic cytotoxic effects of DOX and FAE combinations on HepG2 cells were evaluated using CompuSyn software. The combinations were prepared with a non-fixed ratio of DOX to FAE concentrations. The CompuSyn software calculates the combination index (CI) value by the median effect principle [27]. This software is based on Chou and Talalay’s multiple drug effect equations. The following equation is used to calculate the CI:

$$\:\mathrm{C}\mathrm{I}=\frac{\left(\mathrm{D}\right)1}{\left(\mathrm{D}\mathrm{x}\right)1}+\frac{\left(\mathrm{D}\right)2}{\left(\mathrm{D}\mathrm{x}\right)2}$$

In the equation, (Dx)1 and (Dx)2 indicate the individual dose of DOX and FAE required to inhibit the prescribed level of cell growth. The (D)1 and (D)2 are the doses of individual drugs necessary to produce an equal effect in combination. Combination index (CI) values < 1, =1, and > 1 indicate synergism, additive effect, and antagonism, respectively.

The concentrations used for combination studies were selected based on the individually determined IC₅₀ values of FAE and DOX. Two sub-inhibitory levels corresponding to 25% and 50% of the IC₅₀ were tested to evaluate potential interaction patterns. The concentration pair demonstrating synergistic interaction (CI < 1) was selected for subsequent mechanistic investigations, including apoptosis, autophagy, cell cycle, and Western blot analyses.

### Determination of apoptosis by annexin V-FITC/PI

Apoptosis and necrosis were assessed using the Annexin V-FITC/PI Apoptosis Detection Kit (Abcam, Cambridge, UK) according to the manufacturer’s protocol. HepG2 cells (1 × 10⁵) treated with FAE, DOX, or their combination for 48 h were harvested by trypsinization, washed twice with cold PBS (pH 7.4), and incubated with 0.5 ml of Annexin V-FITC/PI solution for 30 min at room temperature in the dark. Samples were analyzed using an ACEA Novocyte™ flow cytometer (ACEA Biosciences, San Diego, CA, USA). FITC and PI fluorescence signals were detected using FL1 (λ_ex/em: 488/530 nm) and FL2 (λ_ex/em: 535/617 nm) channels, respectively. A total of 12,000 events per sample were acquired, and data were analyzed using ACEA NovoExpress™ software at Nawah Scientific Centre, Mokattam, Cairo, Egypt [28].

### Determination of autophagy

Autophagic cell death was quantified using acridine orange lysosomal stain coupled with flow cytometric analysis. Treated cells (1 × 10⁵) were collected after 48 h, washed twice with cold PBS, and stained with acridine orange (10 µM) at 37 °C for 30 min in the dark. Fluorescence was detected using the FL1 channel (λ_ex/em: 488/530 nm) on the Novocyte™ system. A total of 12,000 events per sample were acquired, and net fluorescence intensity (NFI) was analyzed using NovoExpress™ software at Nawah Scientific Centre, Mokattam, Cairo, Egypt [29].

### Determination of cell cycle arrest

Cell cycle distribution was analyzed following ethanol fixation and propidium iodide (PI) staining. After 48 h of treatment, cells (1 × 10⁵) were fixed in 60% cold ethanol for 1 h at 4 °C, washed twice with PBS, and stained with a solution of RNase A (50 µg/ml) and PI (10 µg/ml) in PBS. After 20 min of incubation at 37 °C in the dark, DNA content was measured using the FL2 channel (λ_ex/em: 535/617 nm) of the Novocyte™ flow cytometer. A total of 12,000 events were recorded per sample, and cell cycle phases were quantified using NovoExpress™ software at Nawah Scientific Centre, Mokattam, Cairo, Egypt [30].

### Cell migration assessment (scratch assay)

The effect of FAE and DOX on HepG2 cell migration was studied using an in vitro scratch assay [31]. HepG2 cells (2 × 10⁵/well) were seeded into coated 12-well plates and incubated overnight to form a confluent monolayer. Scratches were then made, and the wells were washed with PBS. The control wells were replenished with fresh medium, whereas the treated wells were treated with media containing test compounds. Images were captured daily using an inverted microscope, with incubation maintained at 37 °C and 5% CO₂ between observations.

Wound width was measured using MII ImageView software (version 3.7.) at Nawah Scientific Centre, Mokattam, Cairo, Egypt.

The percentage wound closure was determined using the following equation:

Wound closure %: (Wt=0 h – Wt = Δh/Wt = 0 h) × 100, where Wt=0 h is the average width of the wound measured immediately after scratching (time zero), and Wt = Δh is the average width of the wound measured h hours after the scratch is performed. All results are displayed as mean ± SD [32].

### Western blot

HepG2 cells were pre-treated with blank vehicle (0.1% DMSO), FAE, DOX, and combination for 48 h. The cells in each group were washed three times with TBST and then lysed in lysis buffer. The total protein content was determined by the Bradford (BCA) method. Twenty microliters of the lysate were separated by SDS-PAGE and transferred to a PVDF membrane, as described previously [33].

The membranes were washed three times with TBST, then blocked with 5% (*w/v*) skimmed milk in TBST for 1 h at room temperature. The membranes were incubated overnight at 4 °C with primary antibodies diluted in 5% BSA/TBST, including Bax (Invitrogen, Cat. No. MA5-14003; 1:1000), Bcl-2 (Invitrogen, Cat. No. 13–8800; 1:500), LC3B (Cell Signaling Technology, Cat. No. 2775; 1:1000), and GAPDH (Invitrogen, Cat. No. PA1-987; 1:1000). The blots were washed 3 times for 5 min each with TBST and incubated with HRP (horseradish peroxidase) conjugated secondary antibodies (Rabbit anti-mouse IgG (H + L), Goat anti-Rabbit IgG (H + L)) 1:3000 diluted in % skimmed milk in TBST for one hour at room temperature. Subsequently, the bound secondary antibodies were visualized by enhanced chemiluminescence (ECL; Thermo Scientific) and photographed with a Molecular Imager ChemiDoc Gel (Bio-Rad) at Nawah Scientific Centre, Mokattam, Cairo, Egypt. Protein expression was quantified by densitometric analysis of band intensity in Quantity One Software. The optical density values of the protein bands were normalized against those of GAPDH.

### Computational docking analysis

Molecular docking simulations were conducted using the Molecular Operating Environment (MOE 2020.0901, Chemical Computing Group, Montreal, Canada) as the computational software at the Department of Medicinal Chemistry, Faculty of Pharmacy, Assiut University, Assiut/Egypt. The X-ray crystallographic structure of apoptosis regulator Bcl-2 and LC3 (PDB: ID: 4MAN and 6TBE, respectively) complexed with their native substrates (Navitoclax Analog and novobiocin, respectively) was downloaded from the RCSB protein data bank. The method is detailed in the Additional File 2.

### Statistical analysis

All analyses were performed using GraphPad Prism Ver. 10.3.0 (GraphPad Software Inc., San Diego, CA). All data were expressed as mean ± SD, with statistical significance indicated when *P* < 0.05. Statistical comparisons between the control and treated groups were determined using one-way ANOVA with the Tukey-Kramer post hoc test.

## Results and discussion

### Qualitative analysis of fenugreek extract using *UHPLC-QTOF-MS/MS*

**Fig. 1:**
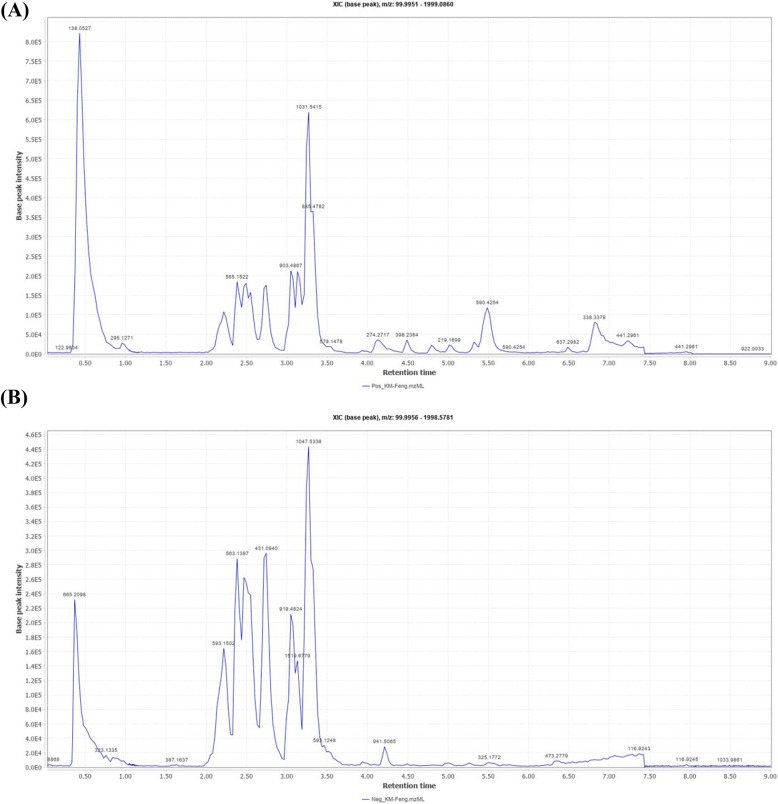
Total ion chromatogram (base peak) of aqueous extract of fenugreek seed (FAE) in (**A**) positive and (**B**) negative ionization modes

The *UHPLC-QTOF-MS/MS* analysis of FAE (Table 1, Fig. 1) indicated the presence of a wide range of compounds with different chemical classes.

As summarized in Tables 1 , 38 compounds were tentatively identified based on MS/MS literature data [34–42].

**Table 1:** Phytochemicals tentatively identified in the aqueous extract of fenugreek seed (Trigonella foenum-graecum Linn)

Comp.No.	Mol. Formula	*m/z*[M- H]-	*m/z*[M+ H]+	Rt	PPM	Name	Class	MS/MS	Ref
1	C_6_H_14_N_4_O_2_		175.1188	0.35	−0.87	Arginine	Amino acid	158.0916, 130.0962	[39]
2	C_5_H_14_NO		104.1067	0.41		Choline	Nitrogenous compound	104.1063, 60.0797, 59.0719	[39]
3	C_5_H_11_NO_2_		118.0859	0.41	−3.01	Betaine (trimethylglycine)	Amino acid	59.0719, 58.0647	[39]
4	C_7_H_8_NO_2_		138.0548	0.44	−1.12	Trigonelline	Alkaloid	138.0527, 94.0646, 92.0480, 78.0338	[37]
5	C_6_H_13_NO_3_		148.0966	0.45	−1.48	4-hydroxyisoleucine	Amino acid	130.0843, 113.0566, 102.0895, 84.0792, 74.0223	[37][39]
6	C_14_H_18_N_2_O_6_		311.1249	0.58	3.66	*N*-gamma-glutamyltyrosine	Amino acid	165.0520, 136.0728	[37]
7	C_18_H_24_O_12_	431.1187		0.60	−1.86	Hydroxybenzoic acid pentosyl hexoside	Phenolic acid	137.0216, 93.0343	[35]
8	C_19_H_26_O_13_	461.1301		0.65	0.08	Hydroxy-methoxybenzoic acid pentosyl hexoside	Phenolic acid	167.0307, 152.0100	
9	C_18_H_24_O_13_	447.1131		0.71	−2.94	Dihydroxybenzoic acid pentosyl hexoside	Phenolic acid	447.1105, 315.0664, 152.0100	[36]
10	C_27_H_30_O_15_	593.1499		2.23	−2.18	Vicenin-2 (Apigenin 3,6-di-C- hexoside)	C-Flavonoid	593.1502, 473.1013, 353.0611, 269.0387	[34]
11	C_28_H_32_O_16_	623.1589		2.35	−4.59	4'-O-Methyllucenin II (Diosmetin 6,8-di-C- hexoside)	C-Flavonoid	533.1287, 503.1146, 413.0876, 383.0765, 353.0611, 299.0520	[43]
12	C_26_H_28_O_14_	563.1395		2.39	2.01	Vicenin-1 or vicenin-3 (Apigenin-6,8-di-C-hexosyl-pentosyl)	C-Flavonoid	563.1387, 473.1013, 353.0611, 269.0387	[34]
13	C_21_H_20_O_11_	447.0924		2.48	−1.98	Orientin or isoorientin; luteolin-C-hexoside	C-Flavonoid	447.0884, 327.0452, 297.0385, 285.0354	[39]
14	C_27_H_30_O_14_	577.1551		2.59	−2.04	Violanthin (6-C-hexosyl-8-C- deoxyhexosyl apigenin) or Isoviolanthin (6-C- deoxyhexosyl −8-C-hexosylapigenin)	C-Flavonoid	577.1544, 457.1114, 353.0611, 269.0558	[37]
15	C_25_H_26_O_13_	533.1287		2.65	−2.56	Apigenin-6,8-di-C-pentoside	C-Flavonoid	515.1135, 473.1089, 443.0972, 383.0765, 353.0611	[36]
16	C_21_H_20_O_10_	431.0975		2.72	−2.02	Vitexin or isovitexin; apigenin-C- hexoside	C-Flavonoid	431.0940, 311.0558, 269.0444	[41]
17	C_22_H_22_O_11_	461.1074		2.87	−3.33	Scoparin or isoscoparin; (Chrysoeriol-C- hexoside)	C-Flavonoid	371.0718, 353.0611, 341.0656, 298.0444	[39]
18	C_22_H_22_O_11_	461.1074		2.87	−3.33	Diosmetin-C-hexoside(Orientin-O-methyl ether)	C-Flavonoid	371.0718, 353.0611, 341.0656, 298.0444	[37][35]
19	C_51_H_86_O_24_	1081.5422		3.00	−1.32	Trigoneoside xviia,Trigoneoside xviib	Saponins	1081.5325, 919.4824, 773.4227, 611.3799, 449.3225	[41]
20	C_44_H_72_O_19_	903.4575		3.01	−2.22	25-Furost-5-en-2,3,22,26-tetrol,3-O-[pentosyl-(1→6)-hexoside]−26-O-hexoside	Saponins	903.4484, 771.4063, 609.3560, 447.3087	[41]
21	C_44_H_74_O_19_	905.4739		3.03	−1.38	Trigoneoside Ia, Ib, xib	Saponins	905.4739, 773.4227, 611.3714, 449.3225	[41]
22	C_45_H_76_O_19_	919.4893		3.06	−1.64	Trigoneoside Xa,Trigoneoside Xb	Saponins	919.4824, 773.4227, 611.3714, 449.3225	[41]
23	C_33_H_54_O_9_		595.3839	3.06	−0.27	2-hydroxy-3-O-[hexosyl] tigogenin; (25)−5-spirostane-2,3-diol 3-O-hexoside	Saponins	433.3300, 289.2153, 271.2036, 253.1904	[38]
24	C_33_H_52_O_8_		577.3732	3.12	−0.51	Trillin; diosgenyl- hexoside	Saponins	415.3194, 271.2036, 253.1904	[37]
25	C_63_H_104_O_33_	1387.6370		3.14	−1.23	26-O-hexosyl-(25)−5-enfurost-3,22,26-triol 3-O- deoxyhexosyl-(1->2)-[hexosyl-(1->6)-hexosyl-(1->3)-hexosyl-(1->4)]-hexoside	Saponins	1387.6298, 1225.5803, 1063.5254, 901.4670, 755.4183, 593.3701	[41]
26	C_68_H_112_O_37_	1519.6789		3.14	−1.36	Trigoneoside Va,Trigoneoside Vb	Saponins	1519.6779, 1387.6298, 1225.5803, 1063.5254, 901.4775, 755.4183	[41]
27	C_33_H_50_O_8_		575.3569	3.18	−1.64	Sceptrumgenin 3-O-hexoside	Saponins	575.3517, 413.3897, 290.2142, 271.2036, 253.1904	
28	C_39_H_62_O_12_		723.4294	3.19	−2.77	Diosgenin-3-O- deoxyhexosyl- hexoside	Saponins	415.3194, 271.2036, 157.0976	[38]
29	C_57_H_94_O_28_	1225.5848		3.20	−0.89	Trigoneoside xiiia,Trigoneoside xiiib	Saponins	1225.5803, 1063.5254, 901.4670, 755.4183, 593.3616	[41]
30	C_51_H_84_O_23_	1063.5311		3.22	−1.85	Trigofoenoside F,Trigoneoside Iva	Saponins	1063.5254, 901.4775, 755.4183, 593.3616, 431.3102, 289.0876	[41]
31	C_44_H_72_O_18_	887.4628		3.23	−2.02	Trigoneoside VIII	Saponins	887.7623, 755.4183, 593.3616, 431.3102, 293.0852	[38]
32	C_51_H_82_O_22_	1045.5208		3.26	−1.62	Trigoneoside XIV	Saponins	1045.5125, 899.4567, 753.3970, 591.3424	[41]
33	C_51_H_84_O_22_	1047.5360		3.28	−2.05	Protodioscin	Saponins	1047.5338, 901.4775, 755.4183, 593.3616, 431.3102, 289.0876	[41]
34	C_45_H_74_O_18_	901.4789		3.31	−1.49	Trigofoenoside A	Saponins	901.4775, 755.4183, 593.3616, 431.3102,	[41]
35	C_30_H_26_O_13_	593.1287		3.39	−2.30	Orientin-2''-O-p-trans-coumarate	Flavonoids	447.0884, 327.0452, 285.0354	[39][42]
36	C_30_H_26_O_12_	577.1332		3.53	−3.38	Vitexin-2''-O-p-trans-coumarate	Flavonoids	431.0940, 311.0558, 269.0387	
37	C_48_H_78_O_18_	941.5092		4.22	−2.48	Soyasaponin I; soyasaponin Bb	Saponins	795.4429, 633.4041, 457.3564, 440.5872	[38]
38	C_16_H_32_O_3_	271.2273		5.69	−2.10	Hydroxy palmitic acid	Fatty acid	253.2117, 225.2198	[36]

16 Steroidal saponins, 11 flavonoid aglycones & glycosides, 4 amino acids, 3 phenolic acids, 2 fatty acids, and one alkaloid were identified in both negative and positive ion modes. The fenugreek phytochemicals were defined based on retention time, accurate mass, and fragmentation pattern. The spectra of the identified compounds were provided in the Additional File (1).

Because herbal extracts have heterogeneous compositions, *UHPLC-QTOF-MS/MS* analysis is usually performed in switching polarity, positive/negative ion mode. Two mean classes of active constituents were tentatively identified in FAE: flavonoid-C-glycosides and steroidal saponins.

The flavonoid-C-glycosides usually show fragmentation patterns different from flavonoid O-glycosides, as the O-C glycosidic bond is easier to be cleaved than the C-glycosidic bond. Breaking the O-C glycosidic bond usually generates signals corresponding to the neutral loss of sugar moieties: 162 for hexoses, 146 for deoxyhexoses, and 132 for pentoses. In contrast, C-C glycoside-aglycone bond resists fragmentation, and intra-glycosidic bonds fragmentation occurs instead, generating fragment ions differing by 30 Da, corresponding to CH-OH moieties, such as 120, 90 for hexoses, 104, 74 for deoxyhexoses, and 90, 60 for pentoses [44] (Table 2). Steroidal saponins possess O–C glycosidic linkages and typically undergo ionization in negative-ion mode to yield [M–H]⁻ ions, along with fragment ions corresponding to the sequential loss of sugar moieties [38].

**Table 2:** Major sugar moieties and aglycone fragments of the C-glycosides (m/z)

	C-flavonoid mass fractions		
Sugar moieties	C-Hexose	−120	−90
	C-deoxyhexose	−104	−74
	C-pentose	−90	−60
Aglycone		Di-C-glycoside	Mono-C-glycoside
	Apigenin	353	311
	Luteolin	369	327
	Chrysoeriol	383	341
	Disometin	383	341

Previous reports on the biological activities of major phytochemicals identified in FAE against liver cancer are briefly summarized:

- *Trigonelline* was the only identified alkaloid in our FAE. Trigonelline increased Bcl-2 expression, an apoptosis inhibitor gene, and decreased Bax expression in liver tissues, thereby preventing hepatic autophagy impairment [45, 46].
- *Flavonoid-C-glycosides* have been previously identified in the aqueous extract of fenugreek seeds. Compounds such as vicenin-2, isovitexin, vitexin, and orientin have been shown to exhibit potent cytotoxic effects against HepG2 cells via binding to topoisomerase IIα, suppressing STAT3 activation; downregulating anti-apoptotic and angiogenic proteins, reducing tumor volume, and promoting apoptosis in a hepatocarcinoma xenograft model. Upregulating Caspase-3, downregulating Bcl-2, and inhibiting autophagy through decreased LC3-II expression. Also reducing viability, inhibiting proliferation, and triggering apoptosis in HepG2 and SK-Hep1 cells, while modulating apoptosis–autophagy interactions. Inhibiting proliferation and migration in Huh-7 cells via NF-κB pathway activation, and inducing both apoptosis and autophagy in HepG2 cells [39, 47–53].
- *UHPLC-QTOF-MS/MS* analysis of FAE revealed its richness in flavonoid C-glycosides. The C-glycosyl flavonoids exhibit higher antioxidant and anti-diabetic activity than their corresponding O-glycosyl flavonoids and aglycones. However, limited literature is available on the anticancer activity of C-glycosyl flavonoids (54).
- *Steroidal saponins* and saponin-glycosides such as trigoneosides, diosgenin, gitogenin, and tigogenin were identified in our FAE. Diosgenin seems to be one of the most promising steroidal saponins due to its mixed anticancer activities that target most hallmarks of cancer: proliferation, growth, invasion and metastasis, angiogenesis, and cell death. Diosgenin inhibited proliferation and potentiated the apoptotic effects of paclitaxel and doxorubicin in HepG-2 cells by blocking STAT3 activation. Diosgenyl-glucosides exhibited potent cytotoxicity by inducing endoplasmic reticulum stress and mitochondrial-mediated apoptotic pathways in liver cancer [13, 55–57].

### Quantitative analysis of fenugreek extract using HPLC

**Fig. 2:**
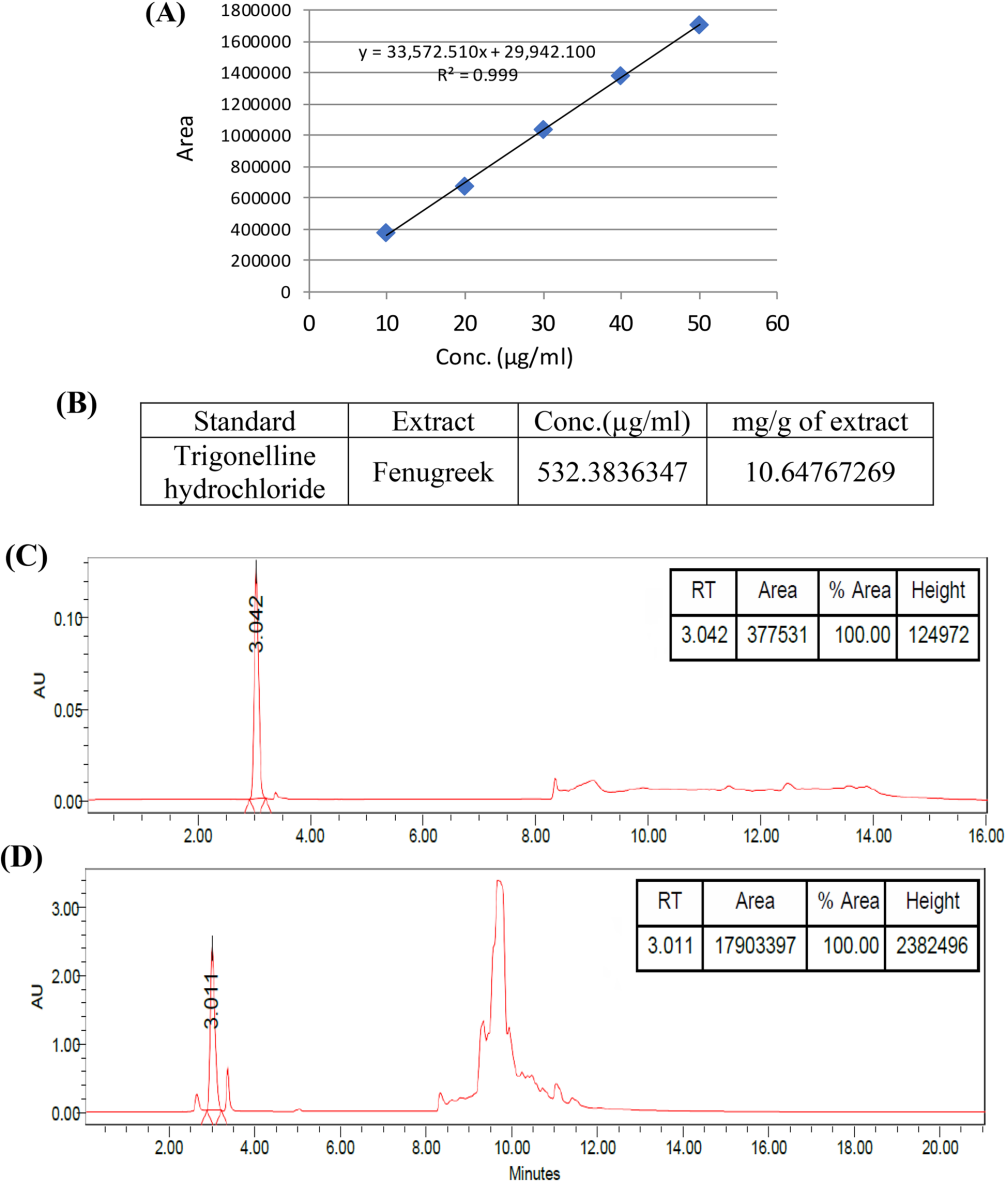
HPLC quantitative analysis results; (**A**) Calibration curve of trigonelline hydrochloride standard solution, (**B**) Measured concentration of trigonelline hydrochloride in FAE using HPLC, (**C**) and (**D**) HPLC chromatogram at 255 nm of a standard solution of trigonelline hydrochloride and FAE at retention times 3.042 and 3.011 minutes, respectively

Quantitative analysis of fenugreek extract by HPLC was done using trigonelline hydrochloride as a standard. A calibration curve for the standard was established (Fig. 2 A), showing its percentage in FAE as 1.065% (Fig. 2B-D).

### Effects of FAE and DOX on the inhibition of HepG2 and BNL CL2 cell proliferation

**Fig. 3:**
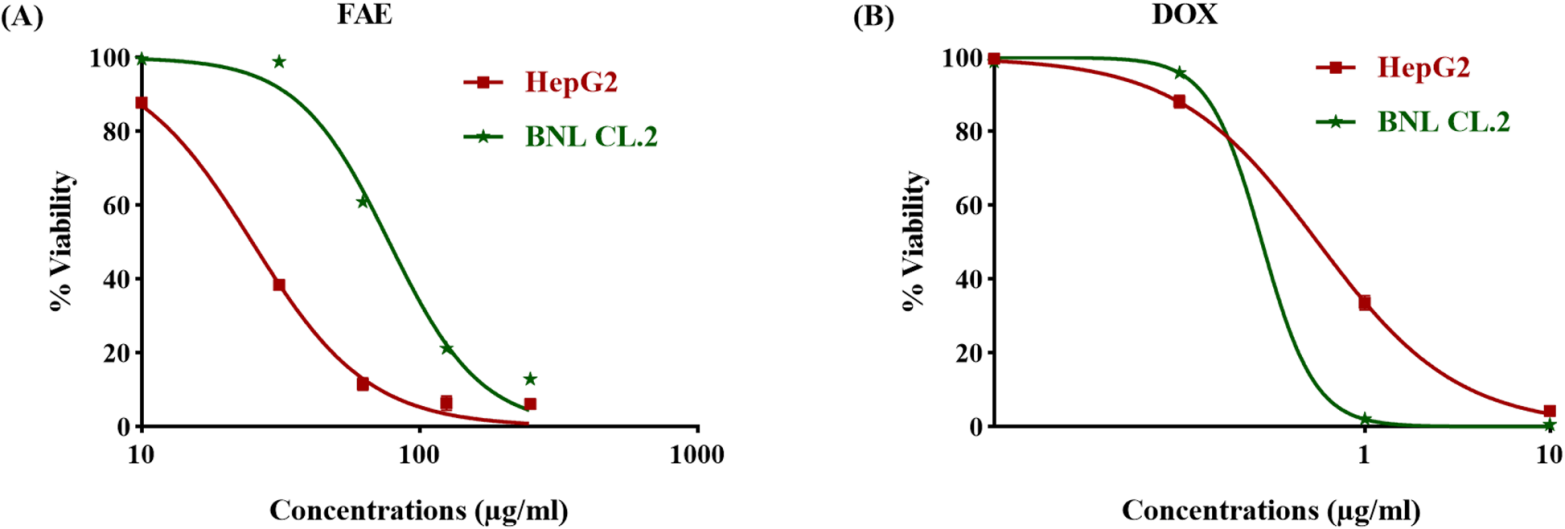
The inhibitory effects of (**A**) Fenugreek aqueous extract (FAE) and (**B**) Doxorubicin (DOX) on the viability of HepG2 cells and BNL CL2 cells following 72 hours of treatment. Cell viability was evaluated using the SRB assay, and the data are expressed as mean ± SD (n = 3)

The cytotoxicity of Fenugreek aqueous extract (FAE) **(**Fig. 3A**)** and Doxorubicin (DOX) **(**Fig. 3B**)** against HepG2 liver cancer cells was examined by SRB assay. After 72 h of treatment, both FAE and DOX inhibited the viability of HepG2 cancer cells in a dose-dependent manner. The IC_50_ values of FAE and Dox were 25.16 ± 1.21 µg/ml and 0.54 ± 0.06 µg/ml, respectively.

Furthermore, the cytotoxic effects of FAE and DOX on the normal mouse liver cells (BNL CL2) were evaluated using the SRB assay to determine their selectivity toward cancer cells **(**Fig. 3**)**. The IC_50_ values for FAE and DOX were 77.73 ± 2.94 µg/ml and 0.26 ± 0.01 µg/ml, respectively, corresponding to selectivity index (SI) values of 3.1 and 0.48. These results demonstrate that FAE exhibits greater selectivity for cancer cells, whereas DOX shows potent cytotoxicity against both cancer and normal cells, suggesting limited selectivity and a greater potential for off-target toxicity.

### Synergistic anticancer effects of DOX and FAE

**Fig. 4:**
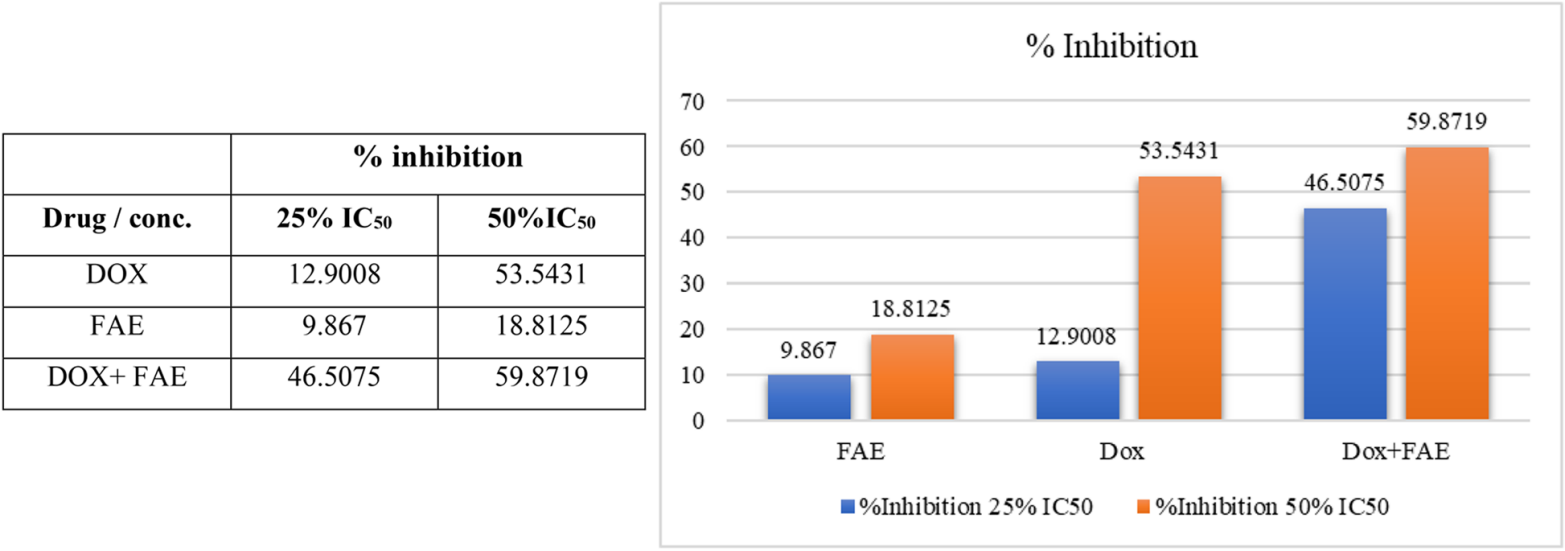
Effect of 25% and 50% IC50 of FAE, DOX, and combinations on the % inhibition of HepG2 cells

To investigate the synergistic cytotoxic effects of the combination of DOX/FAE in liver cancer cells, we examined cytotoxicity in HepG2 cancer cells after treatment with two different concentration ratios of DOX/FAE (25% and 50% IC_50_) (Fig. 4). Two DOX concentrations (0.15 µg/ml and 0.3 µg/ml) with two FAE concentrations (6.25 µg/ml and 12.5 µg/ml) were selected and paired with each other to form four different concentration combinations (DOX/FAE: 0.15/6.25, 0.15/12.5, 0.15/12.5 and 0.3/12.5).

**Fig. 5:**
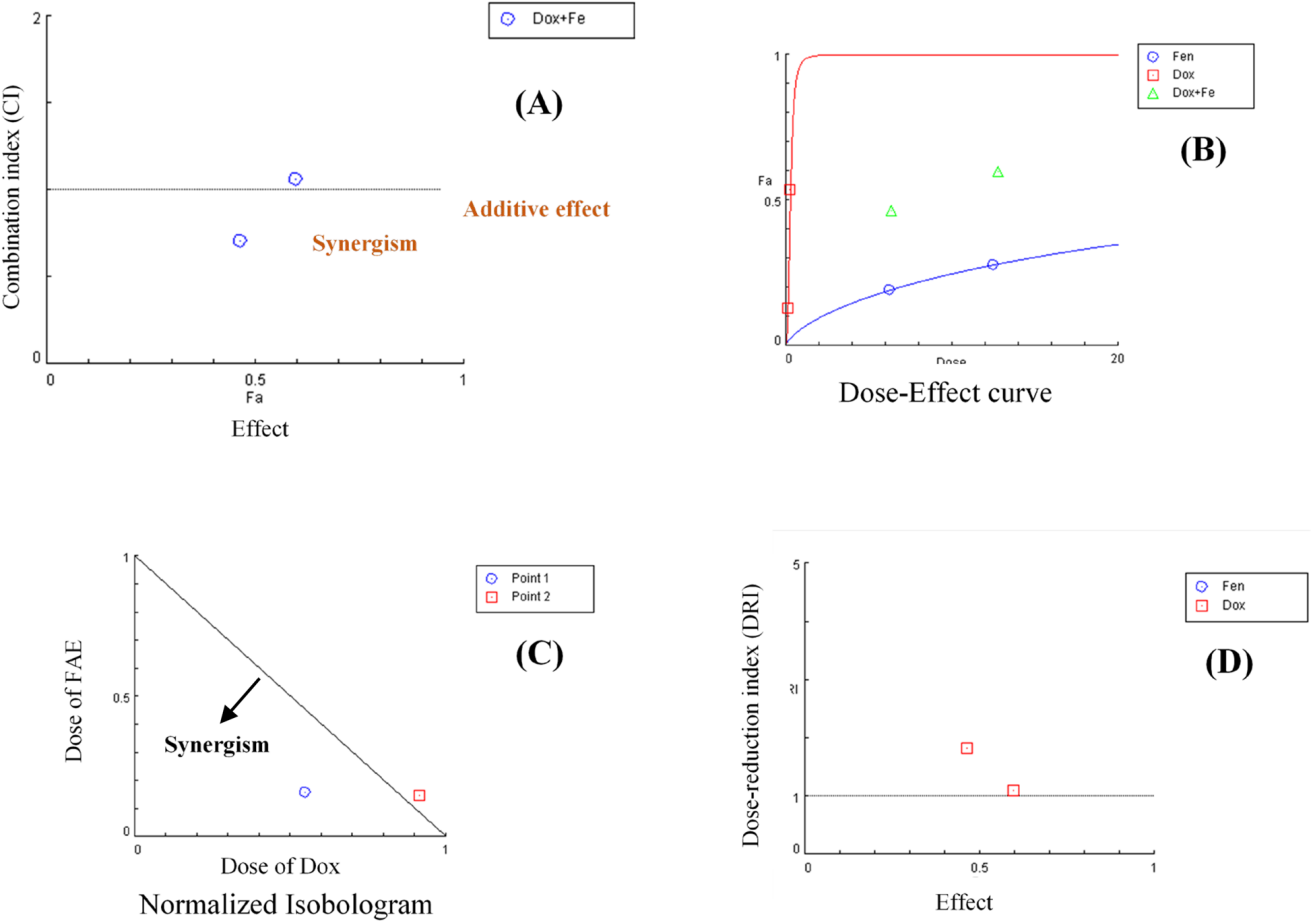
CompuSyn Report, (**A**) CI Data, (**B**) Dose-Effect curve, (**C**) Normalized Isobologram, and (**D**) DRI Data for Non-Constant Combination (DOX/FAE)

At the concentration ratio of (DOX/FAE: 0.15/6.25), the combination exhibits synergistic effects (Combination index CI < 1). While at the concentration ratio of (DOX/FAE: 0.3/12.5), a CI value = 1 indicates an additive cytotoxic effect (Table 3; Fig. 5).

At a concentration ratio of (DOX/FAE: 0.15/6.25), the treated dose of DOX and FAE on HepG2 cancer cells decreased by 1.82% and 6.37%, respectively, according to dose-reduction data.

**Table 3:** Combination index (CI) values for a non-constant combination of DOX and FAE

	Dose FAE	Dose DOX	Effect	CI
Point 1	6.25	0.15	0.46508	0.70705
Point 2	12.5	0.3	0.59872	1.06298

The combined treatment showed enhanced cytotoxic activity compared to monotherapies. Therefore, DOX/FAE may be a promising combination in managing liver cancer, effectively inhibiting tumor growth while possibly reducing doxorubicin-induced toxicity via reducing the DOX dose [58, 59].

### Synergistic induction of cell death by DOX/FAE

**Fig. 6:**
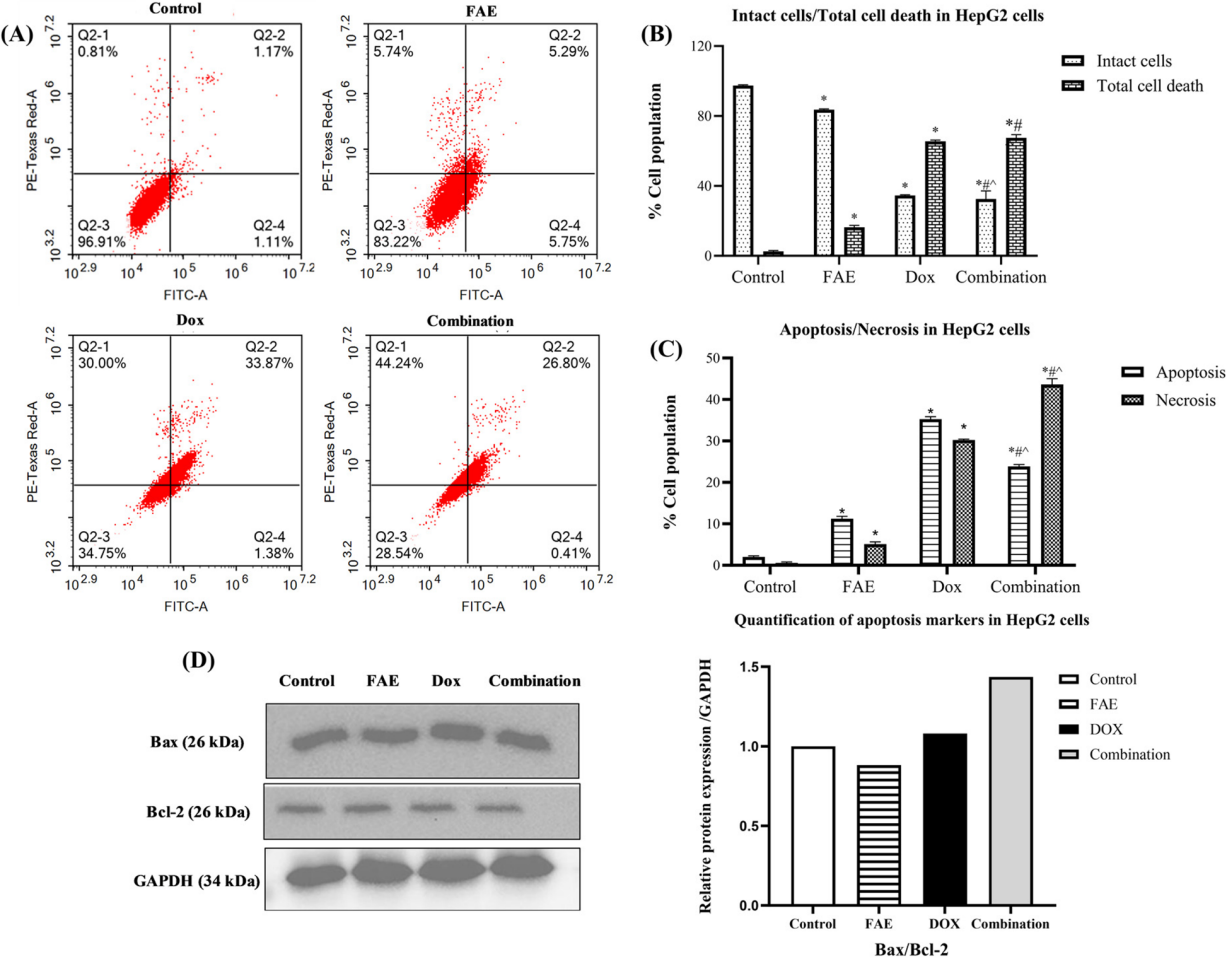
(**A**) Apoptosis/necrosis analysis by flow cytometry using annexin V-FITC/PI double staining assay for HepG2 cells treated with FAE, DOX, and their combination compared with control untreated cells for 48 h, (**B**) Intact cancer cells versus total cell death, and (**C**) Apoptosis/necrosis assessment in HepG2 cells treated with FAE, DOX, and their combination. Data are presented as mean ± SD (n=3). (**D**) The protein expression levels of the apoptotic markers Bax and Bcl-2 in HepG2 cells were determined by Western blot analysis and expressed relative to the control (Uncropped full-length Western blot images are provided in Additional File 3). *Significantly different from control group, #significantly different from DOX treatment, and ^significantly different from FAE treatment. *P*<0.0001

To elucidate the mode of cell death induced by FAE, DOX, and their combination, HepG2 cells were analysed by Annexin V-FITC/PI staining coupled with flow cytometry after 48 h of treatment using the synergistic concentration pair identified by CompuSyn analysis (DOX 0.15 µg/ml + FAE 6.25 µg/ml) (Fig. 6).

FAE significantly triggered both apoptotic and necrotic cell death, with apoptosis as the predominant pathway, consistent with previous findings [19, 60, 61]. FAE induced a total of 16.34 ± 1.12% cell death, comprising 11.24 ± 0.57% apoptosis and 5.1 ± 0.55% necrosis, compared with untreated control HepG2 cells, which exhibited only 2.01 ± 0.27% apoptosis and 0.57 ± 0.24% necrosis **(**Fig. 6B, C**)**. DOX treatment markedly increased both apoptotic (35.27 ± 0.58%) and necrotic cells (30.23 ± 0.2%), reflecting its potent cytotoxicity, as evidenced by the pronounced loss of intact cells and the high proportion of apoptotic cells. Interestingly, the DOX/FAE combination exhibited pronounced cytotoxicity, inducing 71.46 ± 1.8% total cell death, comprising 27.2 ± 0.44% apoptosis and 44.24 ± 1.4% necrosis. Compared with either monotherapy, the combination markedly shifted cell fate toward necrosis, implying a synergistic effect and indicating that cells may rapidly progress from early apoptosis to late apoptosis or necrosis under combined treatment [62]. The results demonstrated that, relative to the control, the combination treatment increased the Bax/Bcl-2 ratio by downregulating Bcl-2 expression **(**Fig. 6D**)**. The Bax/Bcl-2 ratio serves as a key determinant of cell fate and the initiation of apoptosis. A higher Bax/Bcl-2 ratio indicates there are more pro-apoptotic factors (Bax) than anti-apoptotic factors (Bcl-2), enhancing cellular susceptibility to death stimuli and promoting apoptotic cell death [63].

### Synergistic induction of autophagy by DOX/FAE combination

**Fig. 7:**
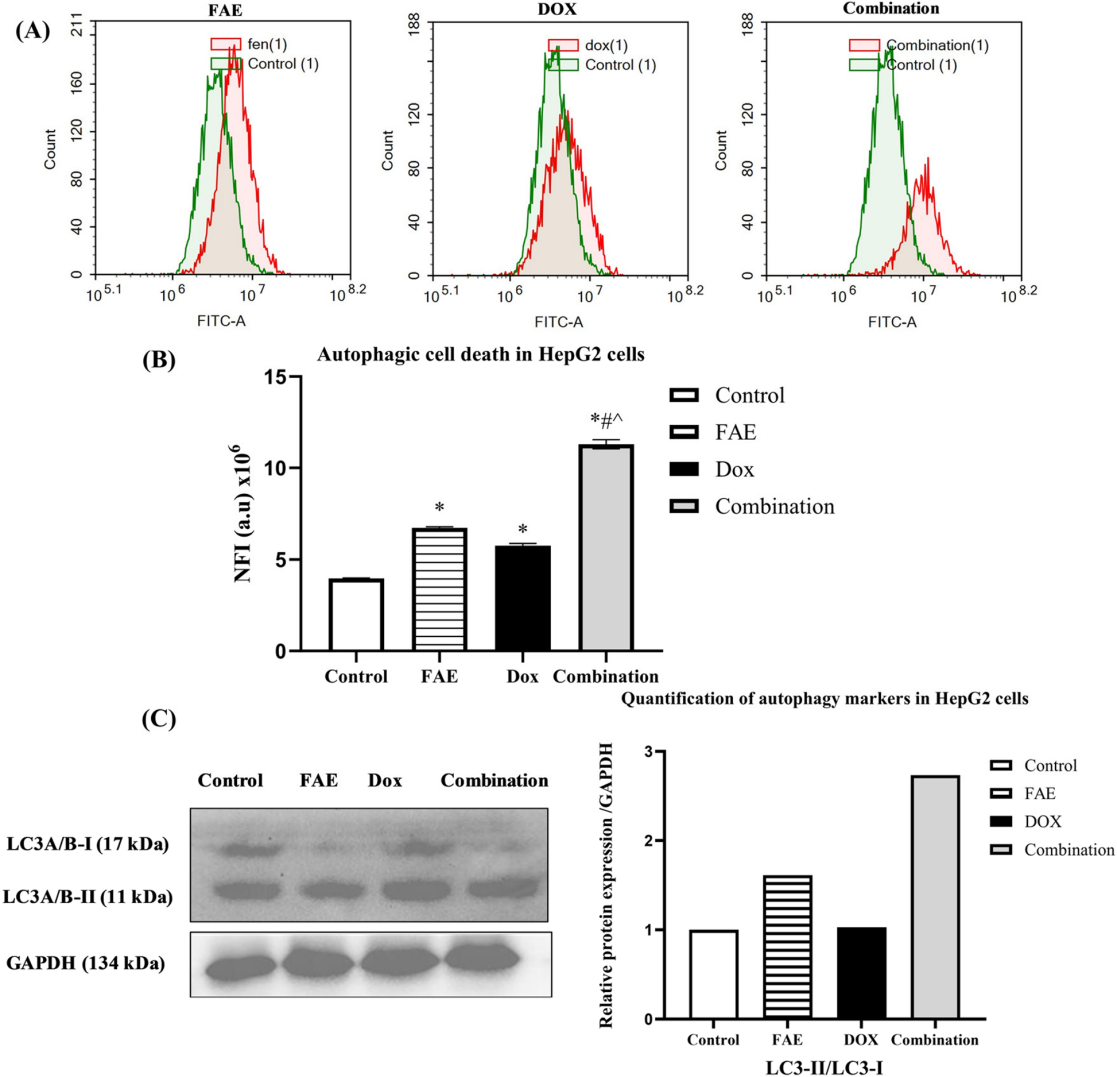
(**A**) Autophagic cell death in HepG2 cells following 24 h exposure to FAE, DOX, and their combination, assessed by acridine orange staining and flow cytometry. (**B**) Net fluorescence intensity (NFI) was plotted relative to the untreated control group. Data are presented as mean ± SD (n=3). (**C**) The protein expression levels of the autophagy marker LC3-II/LC3-I in HepG2 cells were determined by Western blot analysis and normalized to the control (Uncropped full-length Western blot images are provided in Additional File 3). *Significantly different from control group, #significantly different from DOX treatment, and ^significantly different from FAE treatment. *P*<0.0001

To assess programmed cell death via autophagy, HepG2 cells were analyzed using an acridine orange lysosomal stain followed by flow cytometry after 48 h of exposure to FAE, DOX, and their combination (DOX 0.15 µg/ml + FAE 6.25 µg/ml). Both FAE and DOX monotherapies increased autophagic activity, as reflected by net fluorescent intensity (NFI) values of 6.72 ± 0.06 × 10^6 and 5.74 ± 0.12 × 10^6, respectively, compared with 3.962 ± 0.03 × 10^6 in control untreated cells. Notably, the DOX/FAE combination further increased autophagic cell death with NFI of 11.28 ± 0.28 × 10^6 **(**Fig. 7**)**.

These results suggest that autophagy activation contributes to the increased cytotoxicity of the combination, in line with previous studies reporting autophagy-mediated cell death induced by Fenugreek extract and doxorubicin [64–66].

The flow cytometry findings were confirmed by Western blot analysis of the autophagy-related marker LC3-II/LC3-I. During autophagy, the cytosolic microtubule-associated protein light chain 3 (LC3, also known as LC3A/B-I) is cleaved and then conjugated with phosphatidylethanolamine (PE), resulting in the conversion to LC3A/B-II. Consequently, an elevated LC3-II/LC3-I ratio is considered a hallmark indicator of autophagy induction [67].

Our results showed that fenugreek treatment alone significantly raised the LC3-II/LC3-I ratio, indicating autophagy activation. This finding is consistent with previous studies demonstrating that fenugreek seed extracts and their bioactive ingredients induce apoptosis and autophagy in various cancer cell lines [18, 19, 61, 66, 68]. Notably, the effect was more pronounced with the DOX/FAE combination, implying a synergistic role of the combined treatment in boosting autophagic responses.

### Effect of combined DOX/FAE treatment on HepG2 cell cycle distribution

**Fig. 8:**
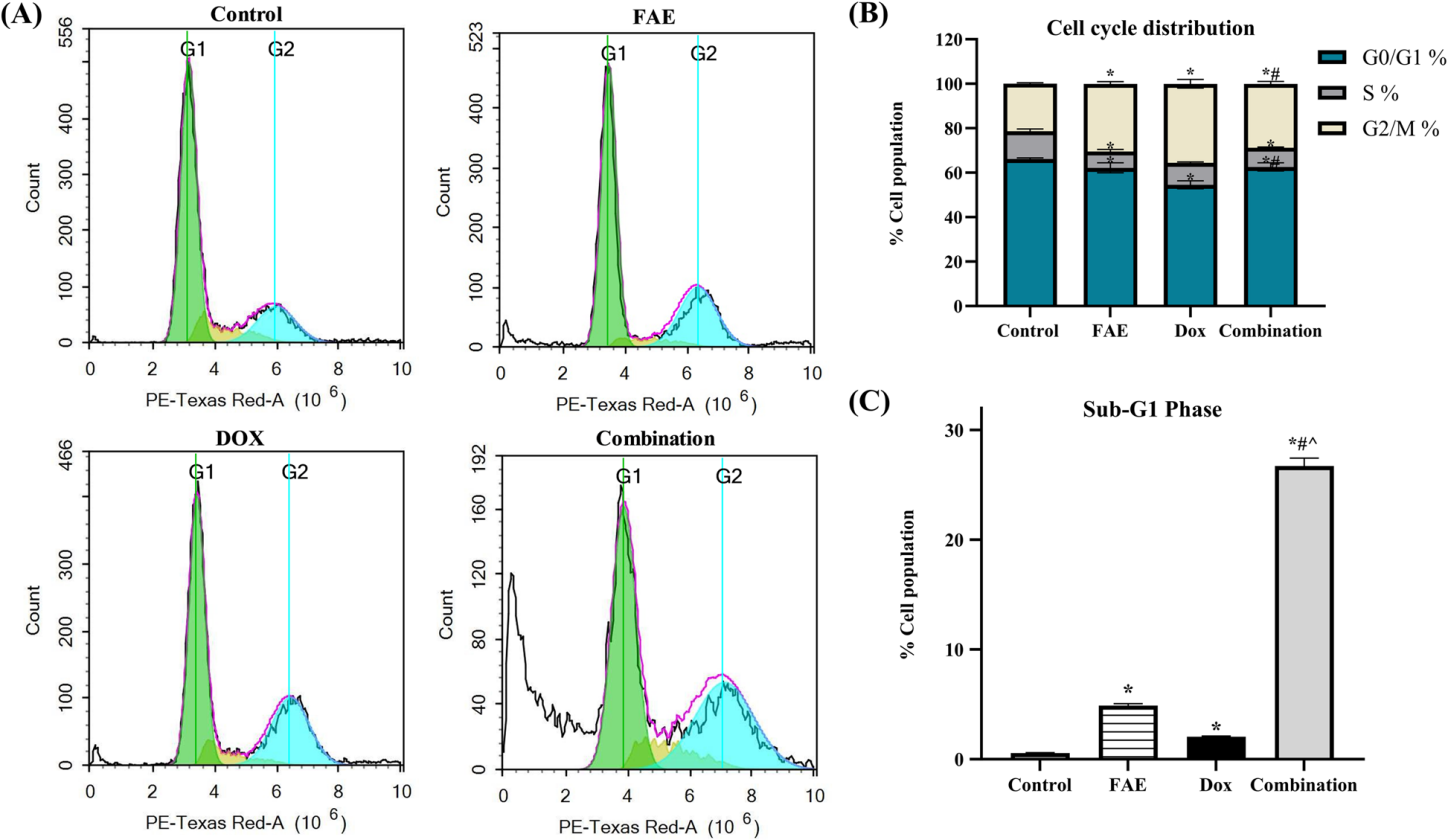
(**A**) Cell cycle arrest assessed by flow cytometry for HepG2 cells treated with FAE, DOX, and their combination compared with control untreated cells for 48 hr. (**B**) Bar graph illustrating the percentage of cells in different cell cycle phases. (**C**) Bar graph illustrating the percentage of cells in the Sub-G1 phase. Data are presented as mean ± SD (n=3). *Significantly different from control group, #significantly different from DOX treatment, and ^significantly different from FAE treatment. *P*<0.0001

Flow cytometry analysis of HepG2 cells treated with FAE, DOX, and their combination (DOX 0.15 µg/ml + FAE 6.25 µg/ml) for 48 h revealed comparable effects on cell cycle distribution. Both treatments significantly reduced the G0/G1 population (65.61 ± 0.45% in control vs. 60.25 ± 2.11% with FAE and 53.63 ± 1.75% with DOX) and slightly decreased the S-phase fraction (12.48 ± 0.97% vs. 7.36 ± 0.89% and 10.11 ± 0.34%, respectively). These alterations were accompanied by a marked increase in the G2/M phase, indicating mitotic arrest (21.35 ± 0.45% in control vs. 32.20 ± 0.90% with FAE and 36.26 ± 1.98% with DOX) **(**Fig. 8 A, B). Increased cell death was reflected by a rise in the sub-G1 fraction (0.56 ± 0.04% in control vs. 5.27 ± 0.18% with FAE and 2.05 ± 0.06% with DOX), indicating fragmented nuclei with diminished DNA content **(**Fig. 8 C**).** These findings are consistent with previous studies demonstrating that fenugreek extract and DOX induce G2/M arrest implicating their role in inducing apoptosis [69, 70].

The combination treatment markedly altered cell cycle distribution compared with monotherapies. DOX/FAE treatment induced pronounced G2/M phase retention (39.17 ± 1.3%), indicating robust mitotic arrest. Consequently, it significantly raised the Sub-G1 population to 28.7 ± 1.1%, representing ~ 13.7- and ~ 5.5-fold increases compared to DOX- and FAE-treated cells, respectively, indicating a synergistic effect. A substantial reduction in G0/G1% (48.95 ± 2.11%) was observed compared with control and a modest decrease relative to DOX, while S-phase proportions remained mainly unchanged (Fig. 8 A, B).

These results suggested that the DOX/FAE combination might be potent at targeting rapidly dividing HepG2 cells by effectively arresting them in the sub-G1 and G2/M phases, inducing both apoptosis and necrosis. These results are supported by Annexin V-FITC/PI staining coupled with flow cytometry, as previously mentioned and agreed with the previous study [71]. This combination therapy may be a more effective strategy for inhibiting HepG2 cell proliferation and inducing cell death than monotherapies.

Programmed cell death type I (apoptosis), type II (autophagy), and necrosis are some of the mechanisms involved in cell death produced by plant products [18]. Autophagy is considered to precede apoptosis in the sequence of programmed cell death. The link between these two pathways could be explained by the capacity of autophagy to reduce intracellular levels of pro-apoptotic proteins, thereby elevating the stress threshold required to induce apoptosis [72]. Our findings indicated that DOX/FAE combined treatment induced cell death in HepG2 cells by arresting cells in the Sub-G1 phase of the cell cycle and inducing autophagy, apoptosis, and necrosis.

Although many studies have demonstrated the cytotoxic effects of fenugreek extracts in experimental cancer models using cell lines, this is the first report showing that FAE enhances the cytotoxicity of DOX in vitro against HepG2 cells. FAE enhanced the anti-proliferative effect of doxorubicin and showed selective cytotoxicity against cancer cells.

Combination strategies integrating natural products with chemotherapeutic agents have also gained interest. For example, fenugreek extracts have demonstrated enhanced anticancer effects when combined with doxorubicin or cisplatin in glioblastoma models [62], supporting the rationale for exploring similar combination strategies in liver cancer. However, few studies have systematically investigated the interaction between crude aqueous fenugreek extract and doxorubicin in HCC models, integrating phytochemical profiling, synergy quantification, and mechanistic validation.

### Assessment of cell migration by scratch assay

**Fig. 9:**
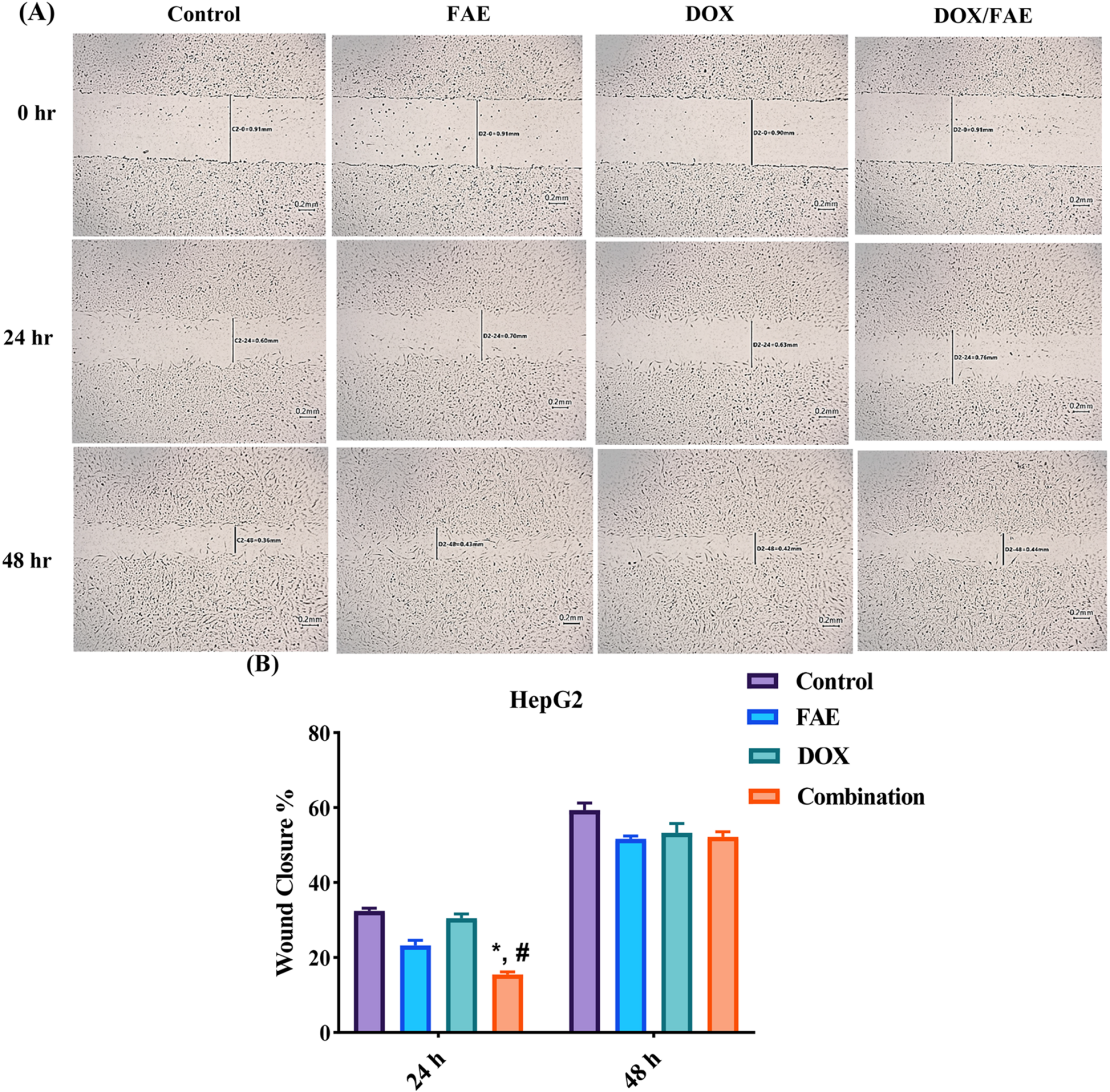
(**A**) Representative images showing the scratch width of HepG2 cells at 0-, 24-, and 48-hours following treatment with FAE, DOX, and their combination, compared with control untreated cells. (**B**) The bar graph represents the percentage of scratch closure. Data are presented as mean ± SD (n=3). *Significantly different from control group, #significantly different from DOX treatment (*P*<0.0001)

Hepatocellular carcinoma (HCC) has a poor prognosis, mainly due to its high metastatic potential [73]. Cancer migration and invasion are crucial for tumor progression and metastasis, and their inhibition represents a promising target for anti-metastatic therapy [74]. The scratch assay was used to assess the effects of FAE, DOX, and their combination on HepG2 cell migration in vitro. Representative images of scratch width at 0, 24, and 48 are illustrated in Fig. 9 A, and the percentage of scratch closure was calculated (Fig. 9 B).

Figure 9 A and B indicate that untreated HepG2 cells exhibited a clear migratory tendency, with scratch closure of 32.42 ± 0.77% at 24 h and 59.34 ± 1.91% at 48 h, reaching complete closure by 72 h. In contrast, treatment with FAE, DOX, or their combination had minimal impact on cell migration, as all treated groups showed complete closure at 72 h. At 24 and 48 h, slightly lower but statistically insignificant closure percentages were observed in DOX (30.48 ± 1.13% and 53.33 ± 2.44%), FAE (23.20 ± 1.38% and 51.67 ± 0.78%), and the combination (15.47 ± 0.68% and 52.22 ± 1.35%).

These findings are consistent with previous studies demonstrating the limited efficacy of DOX in metastasis management [75, 76]. In contrast, they differ from studies indicating that fenugreek extracts and their constituents inhibit invasion and migration in various malignancies [19, 60, 69, 77]. While FAE substantially enhanced DOX cytotoxicity and displayed synergistic effects in combination, it had little effect on DOX-associated metastasis.

Given the complexity of metastasis and the potential for dose-dependent responses, further studies evaluating different concentration ratios are required to define the therapeutic window and explore the possibility of DOX/FAE as an anti-metastatic treatment [78].

The present study employed the HepG2 cell line as a well-established in vitro model of HCC [79], enabling a focused proof-of-concept evaluation of the potential synergistic interaction between fenugreek aqueous extract and doxorubicin under controlled conditions. However, given the molecular heterogeneity of HCC, validation in additional liver cancer cell lines with diverse genetic backgrounds is necessary. Moreover, as the findings are limited to in vitro experiments, further in vivo studies are required to confirm therapeutic efficacy, safety, and translational relevance.

### In-silico molecular docking of major constituents in FAE

When a plant extract exhibits notable biological activity, isolating and identifying its bioactive compounds enables in silico analyses to predict potential drug targets. In recent years, molecular docking has developed as a crucial computer-assisted drug design tool. It allows for the virtual screening of vast libraries of compounds, ranks their binding affinities, and offers structural information on how ligands interact with target proteins. Molecular docking helps develop new treatments by predicting the binding mode of a ligand to a protein with a known three-dimensional structure **[**80, 81**]**.

In the present study, some of the most abundant constituents, steroidal saponins and C-flavonoid glycosides [82, 83], identified in FAE were selected for molecular docking analysis. This in silico approach was employed to complement the experimental findings and to provide molecular-level insights into the binding interactions between the selected compounds and the target proteins. It also clarifies the possible contribution of the extract constituents to the observed biological activities.

The docking analysis (Table 4) revealed distinct interaction profiles of the extract constituents with Bcl-2 and LC3, highlighting binding energy scores (S), ligand–amino acid interaction types, and corresponding intermolecular distances (Å) for the most stable complexes. The remaining docking data are presented in the Additional File (2) (Table 1 S). The docking score (S) and the number of hydrogen bonds served as primary indicators of inhibitory potential, with lower S values corresponding to a more favorable Gibbs free energy of binding, and a greater number of hydrogen bonds indicating enhanced stability and affinity of the ligand–receptor complex.

**Table 4:** Summary of Molecular docking analysis of compounds of high binding scores against Bcl-2 (Pdb: 4MAN) and LC3 (Pdb: 6TBE)* proteins

Compounds	Bcl-2		LC3	
	Docking Score	Interactions residues (Distance)	Docking Score	Interactions residues (Distance)
Co-crystalized ligand	−11.17	ASP 100 (2.04)GLU 133 (2.97)GLY 142 (2.4)GLU 133 (2.27)ARG 104 (2.36)TYR 105 (2.86)	−10.4	GLN 30 (2.33)LEU 57 (3.24)HIS 31 (2.91)LYS 34 (2.79)LEU 57 (3.21)
Doxorubicin	−10.22	GLU 133 (3.13)ASP 108 (2.2)ARG 143 (2.94)TYR 105 (2.61)	−9.6	GLU 23 (2.09)LYS 34 (2.17)LEU 57 (2.03)HIS 31 (3.40)LEU 57 (2.64)
Protodioscin	−11.38	ASN 140 (3.07)ASP 100 (2.83)ASP 100 (2.94)ARG 103 (2.45)	−10.59	LEU 57 2.01GLU 66 (2.17)LYS 53 (2.14)LYS 53 (2.47)
Trigofoenoside A	−11.11	TYR 199 (2.52)GLU 133 (2.91)GLU 111 (2.1)	−10.01	LEU 57 (2.66)GLN 30 (2.99)HIS 31 (2.99)
Trigoneoside Va	−12.19	GLU 133 (2.89)GLY 142 (3.00)ASP 137 (3.16)	−13.26	LEU 57 (2.35)LYS 55 (2.34)LYS 34 (2.01)GLN 26 (2.23)

*Co-crystallized ligand (4MAN) is Navitoclax Analog) and (6TBE) is Novobiocin). Docking score measured in (kcal/mol) and distances in Å

**Fig. 10:**
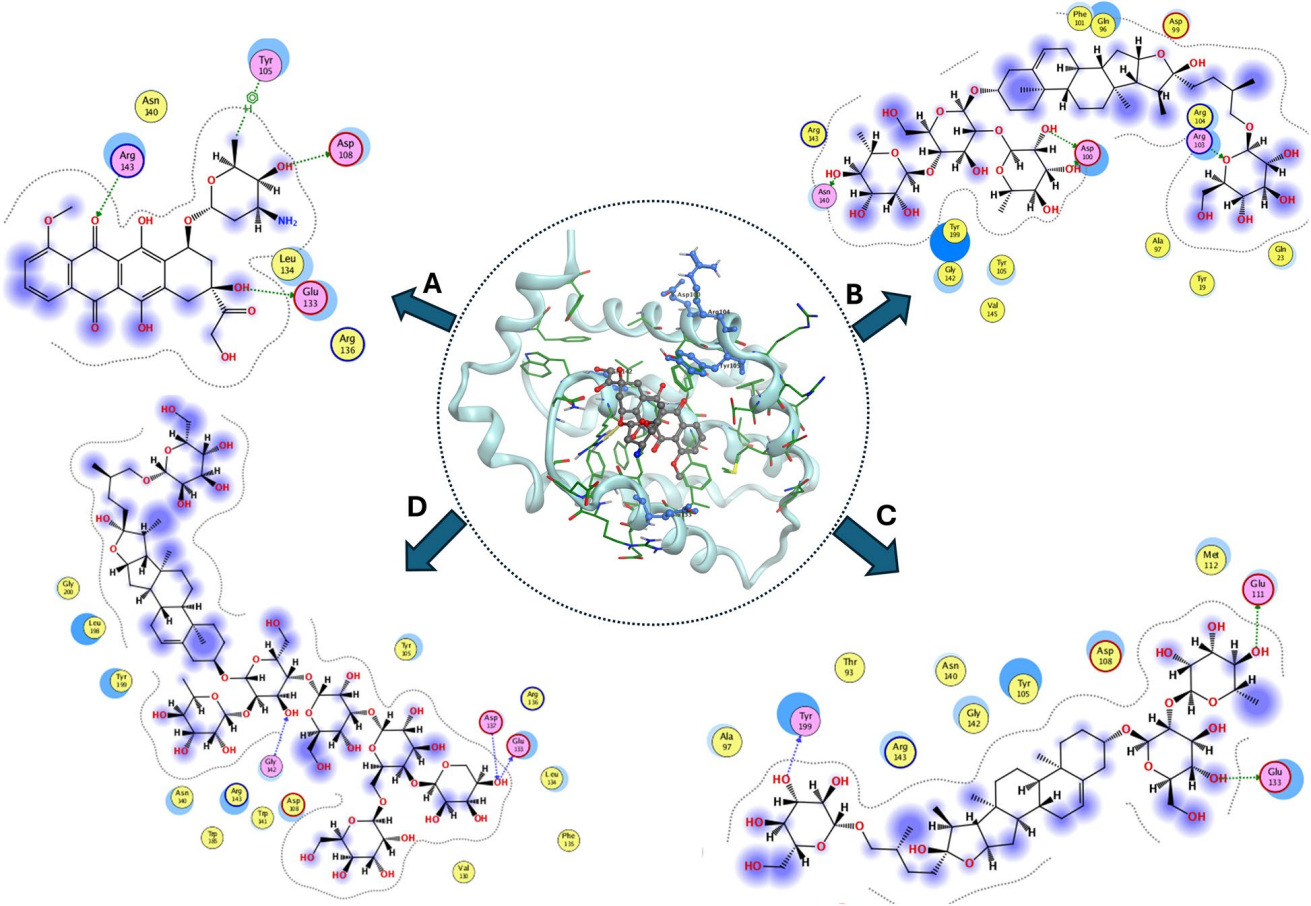
2D display of **(A)** Doxorubicin, **(B)** Protodioscin, **(C)**Trigofoenoside A, and **(D)** Trigoneoside Va of FAE docking against LC3 protein (Pdb: 6TBE)

The two-dimensional theoretical binding modes for the candidate compounds (Fig. 10) exhibited substantial resemblance to the binding pattern observed in the native ligand (Navitoclax Analog)/Bcl-2 complex. Three saponins, *Protodioscin, Trigofoenoside A*, and *Trigoneoside Va*, demonstrated superior docking scores (−11.38, −11.11, and − 12.19 kcal/mol, respectively) relative to the other candidates (ranging from − 9.61 to − 6.78 kcal/mol) as well as reference ligands *doxorubicin* and native ligand (−10.12 and − 11.17 kcal/mol, respectively). The docking outcomes revealed that doxorubicin binds to the Bcl-2 active site through hydrogen bonding with GLU 133 (3.13 Å) and ARG 143 (2.94 Å), with additional binding to ASP 108 (2.2 Å). It also forms hydrophobic interactions with TYR 105 (2.6 Å). While *Trigoneoside Va* showed a notably higher docking score than the other constituents of the extract, reflecting its strong affinity toward the Bcl-2 active site due to stable hydrogen bonding with the key active-site residues GLU133 and GLY142, along with an additional interaction with ASP137 (2.89, 3.00, and 3.16 Å, respectively). In contrast, the Bcl-2/*Protodioscin* complex involves two hydrogen bonds with ASP100 (distances of 2.83 Å and 2.94 Å), the other with ARG103 (2.45 Å), and an additional hydrogen bond with ASN140 (3.07 Å). These interactions suggest that *Protodioscin* effectively stabilizes within the catalytic pocket through multiple hydrogen bonds, which may contribute to its enhanced binding energy. However, *Trigofoenoside A* binds to TYR 199, GLU 133, and GLU 111 at comparatively short distances of 2.52, 2.91, and 2.1 Å, respectively.

**Fig. 11:**
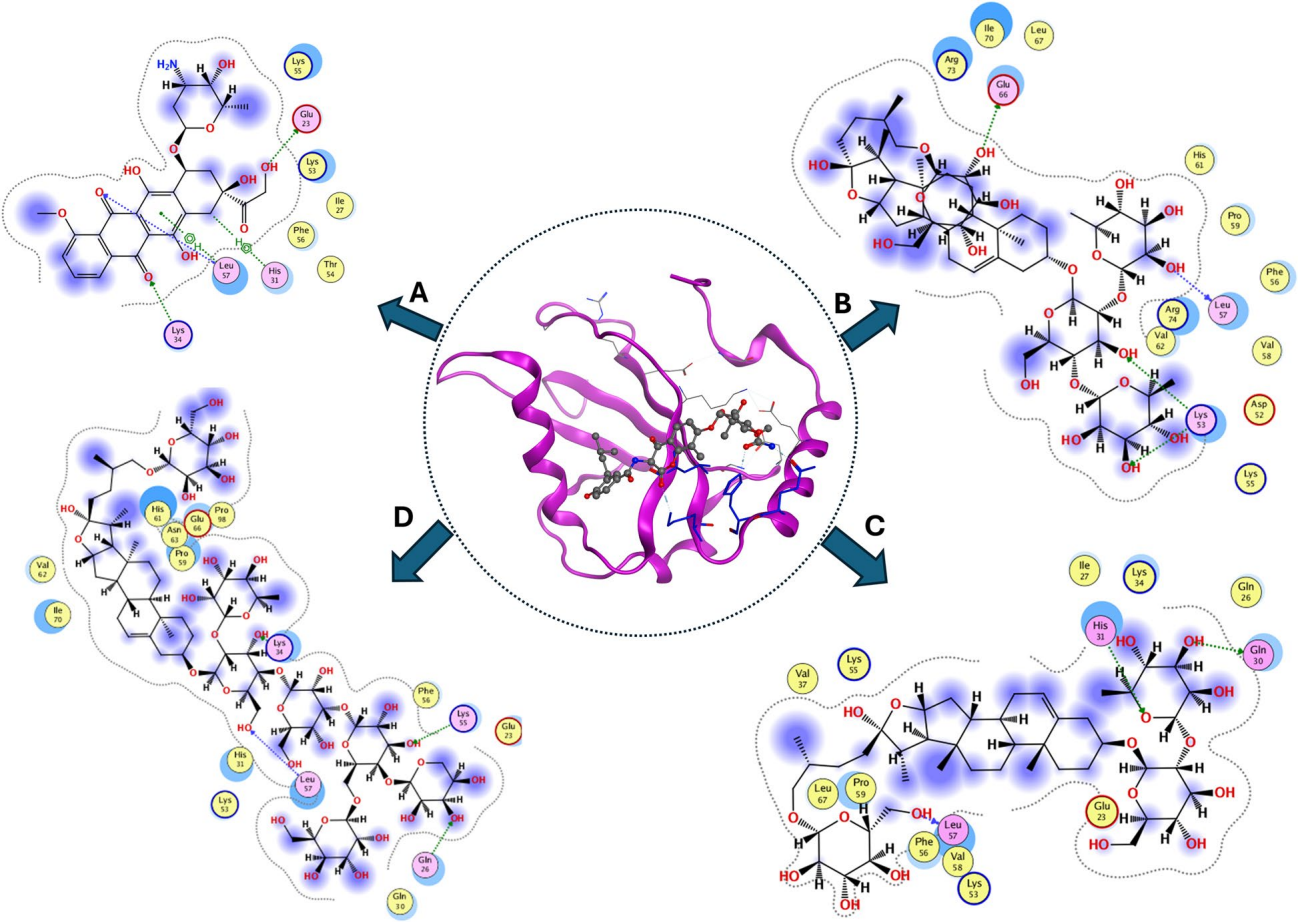
2D display of **(A)** Doxorubicin, **(B)** Protodioscin, **(C)** Trigofoenoside A, and **(D)** Trigoneoside Va of FAE docking against LC3 protein (Pdb: 6TBE). Molecular docking analysis suggested that selected steroidal saponins from FAE may interact favorably with Bcl-2 and LC3, providing a plausible molecular basis for the biological effects observed in vitro, and suggesting significant dual affinity toward both targets. This concordance indicates that FAE constituents may exert their effects through a complementary mechanism of interaction within the apoptotic and autophagic pathways, thereby enhancing doxorubicin's activities. Nevertheless, docking outcomes are predictive rather than confirmatory

Interestingly, the docking results for the targeted molecules in relation to Bcl-2 (Fig. 10) matched those for LC3 (Fig. 11). *Protodioscin*, *Trigofoenoside A*, and *Trigoneoside Va* with LC3 showed comparatively higher binding affinities than other targeted compounds (−10.59, −10.01, and − 13.26 kcal/mol, respectively), and both *Doxorubicin* and reference drug (−9.6 and − 10.4 kcal/mol, respectively), as displayed in Table 4. Other compounds docked with a range of −9.45 to −8 kcal/mol.

Similar to the reference substrate, *doxorubicin* occupied the active pocket through interacting with GLU 23, LYS 34, and LEU 57 besides hydrophobic interactions with HIS 31 and LEU 57 at distances (2.09, 2.17, 2.03, 3.4, and 2.64Å, respectively). In addition, *Trigoneoside Va* interacted with the essential residues by forming hydrogen bonds with LEU 57 and LYS 34, with additional binding to LYS 55 and GLN 26 at relatively short distances (2.35, 2.01, 2.34, and 2.23 Å, respectively). That may likely contribute to the enhanced stability and binding affinity of Trigoneoside Va within the active site. At the same time, the Protodioscin/LC3 complex is stabilized through hydrogen bonding with LEU 57 (2.01 Å). It also formed three hydrogen bonds with GLU 66 (2.17 Å) and LYS 53 (2.14 and 2.47 Å) at short distances, which may confer good affinity and high binding energy. *Trigofoenoside A* docked to the active pocket residues LEU 57, HIS 31, and GLN 30 by forming three hydrogen bonds at distances (2.66, 2.99, and 2.99 Å, respectively).

Molecular docking analysis suggested that selected steroidal saponins from FAE may interact favorably with Bcl-2 and LC3, providing a plausible molecular basis for the biological effects observed in vitro, and suggesting significant dual affinity toward both targets. This concordance indicates that FAE constituents may exert their effects through a complementary mechanism of interaction within the apoptotic and autophagic pathways, thereby enhancing doxorubicin’s activities. Nevertheless, docking outcomes are predictive rather than confirmatory.

## Conclusion

The present study provides in vitro evidence that fenugreek aqueous extract potentiates doxorubicin-induced cytotoxicity in HepG2 cells by modulating apoptotic, autophagic, and cell-cycle regulatory pathways. The observed enhancement enabled a partial reduction in the doxorubicin dose while maintaining anticancer efficacy, suggesting a potential strategy to reduce chemotherapy-associated toxicity. While molecular docking supported possible interactions between major FAE constituents and key regulatory proteins, further validation in additional cellular (e.g., Huh7 or Hep3B) and in vivo models is required before therapeutic relevance can be inferred.

## Supplementary Information

Supplementary Material 1: Additional file 1 (.pptx): UHPLC-QTOF-MS/MS spectra of tentatively identified compounds in fenugreek seed aqueous extract (FAE)

Supplementary Material 2: Additional file 2 (.docx): Summary of Molecular docking analysis results of compounds from FAE with apoptosis (Bcl-2) and autophagy (LC3) targets, including docking scores and amino acid interactions

Supplementary Material 3: Additional file 3 (.docx): "Uncropped, full-length original Western blot images corresponding to all cropped panels shown in Figures 6 and 7

## Data Availability

The authors declare that the data supporting the findings of this study are available within the paper and its Supplementary Information files. Any raw data files in a format other than the original are available from the corresponding author upon reasonable request.
