# Supplementary material for: Fenugreek seed extract–doxorubicin synergy against hepatocellular carcinoma in HepG2 cells: in vitro and in silico mechanistic studies
Source: BMC Complement Med Ther. 2026 May 6;26:171. doi: 10.1186/s12906-026-05386-3 (PMC13151130; doi:10.1186/s12906-026-05386-3)
Supplement: Supplementary file 1 — Supplementary Material 1: Additional file 1 (.pptx): UHPLC-QTOF-MS/MS spectra of tentatively identified compounds in fenugreek seed aqueous extract (FAE) [file 12906_2026_5386_MOESM1_ESM.pptx]

## Slide 1
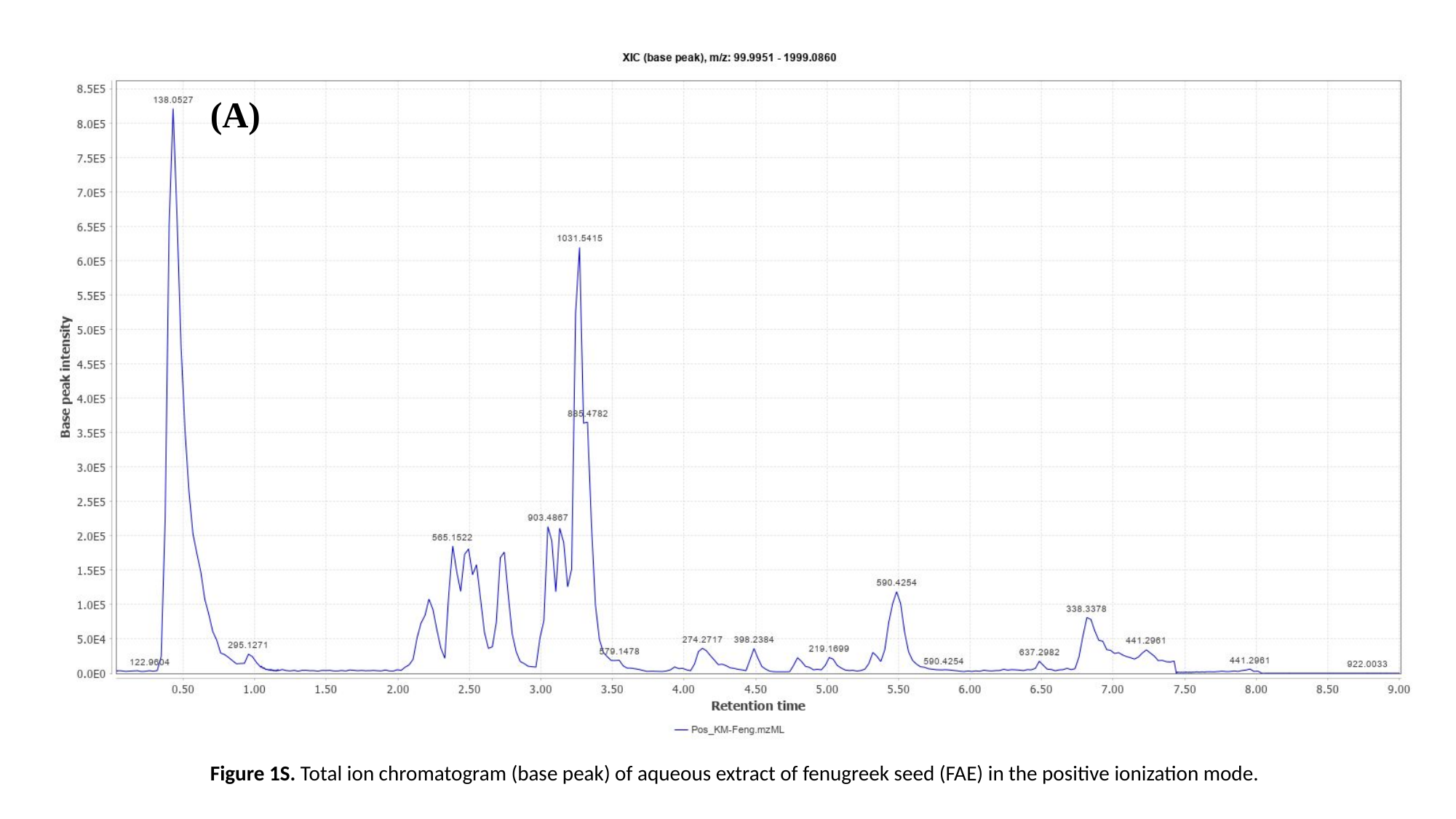

(A)
Figure 1S. Total ion chromatogram (base peak) of aqueous extract of fenugreek seed (FAE) in the positive ionization mode.

## Slide 2
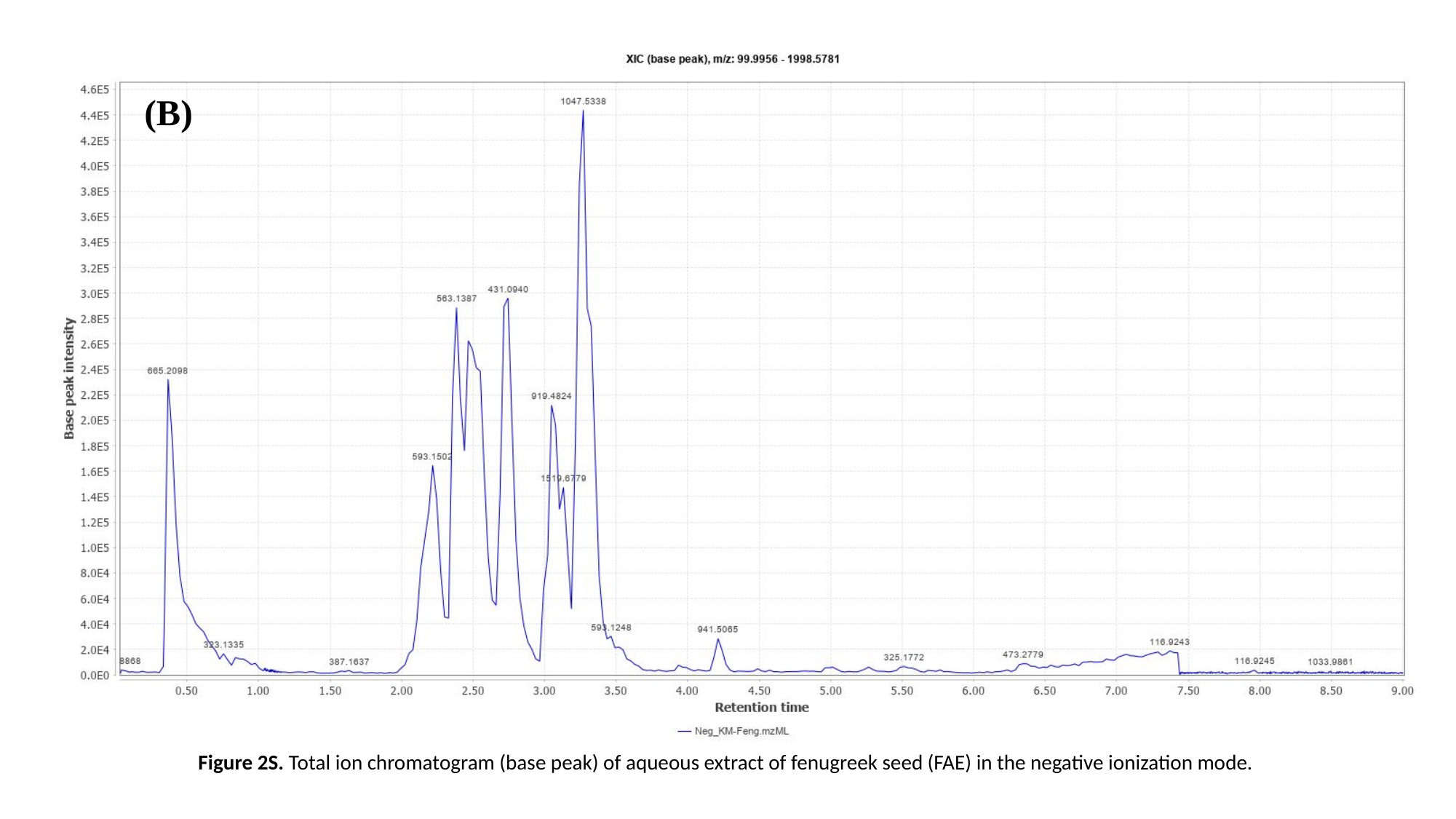

(B)
Figure 2S. Total ion chromatogram (base peak) of aqueous extract of fenugreek seed (FAE) in the negative ionization mode.

## Slide 3
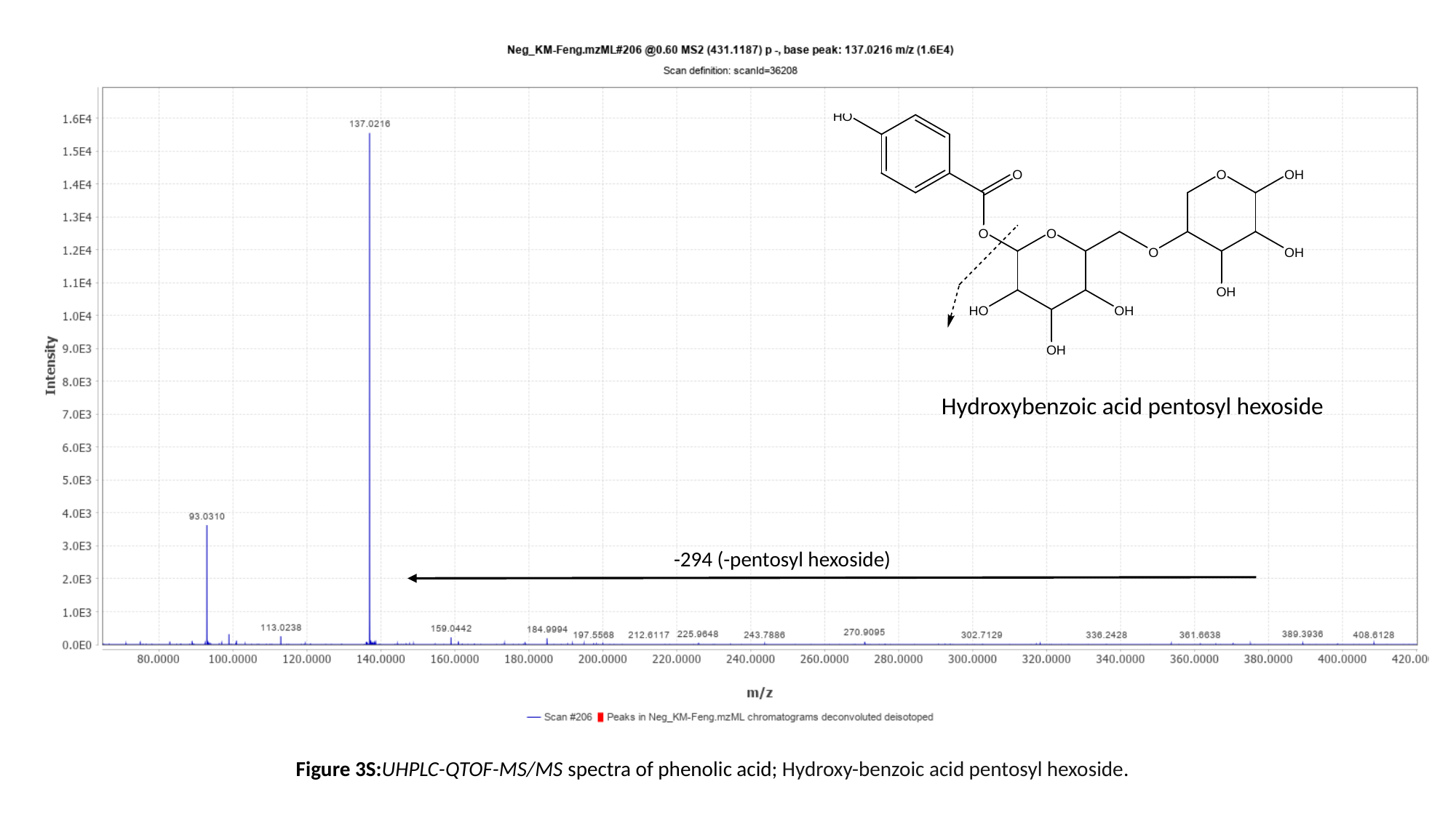

Hydroxybenzoic acid pentosyl hexoside
-294 (-pentosyl hexoside)
Figure 3S:UHPLC-QTOF-MS/MS spectra of phenolic acid; Hydroxy-benzoic acid pentosyl hexoside.

## Slide 4
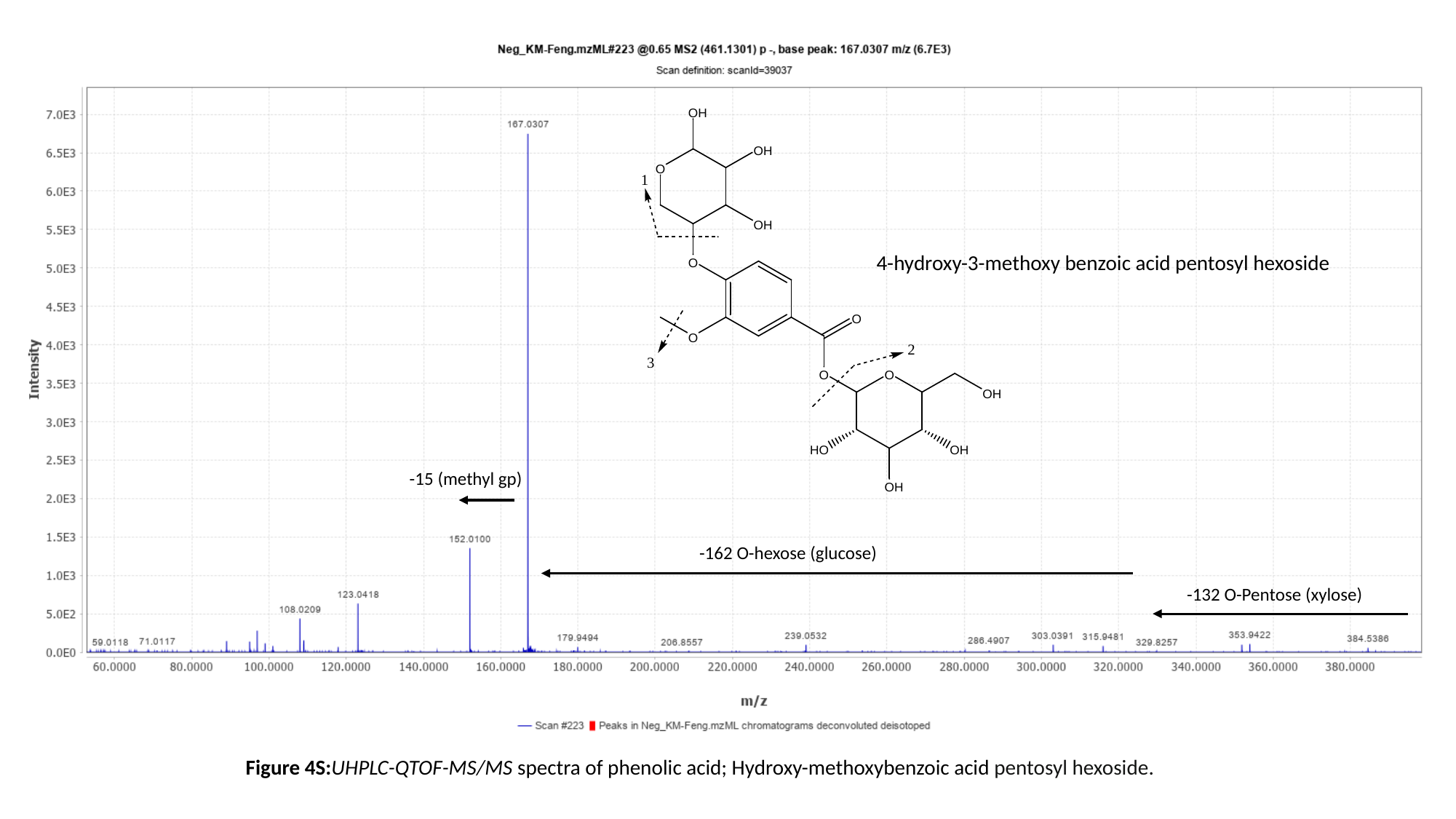

4-hydroxy-3-methoxy benzoic acid pentosyl hexoside
-15 (methyl gp)
-162 O-hexose (glucose)
-132 O-Pentose (xylose)
Figure 4S:UHPLC-QTOF-MS/MS spectra of phenolic acid; Hydroxy-methoxybenzoic acid pentosyl hexoside.

## Slide 5
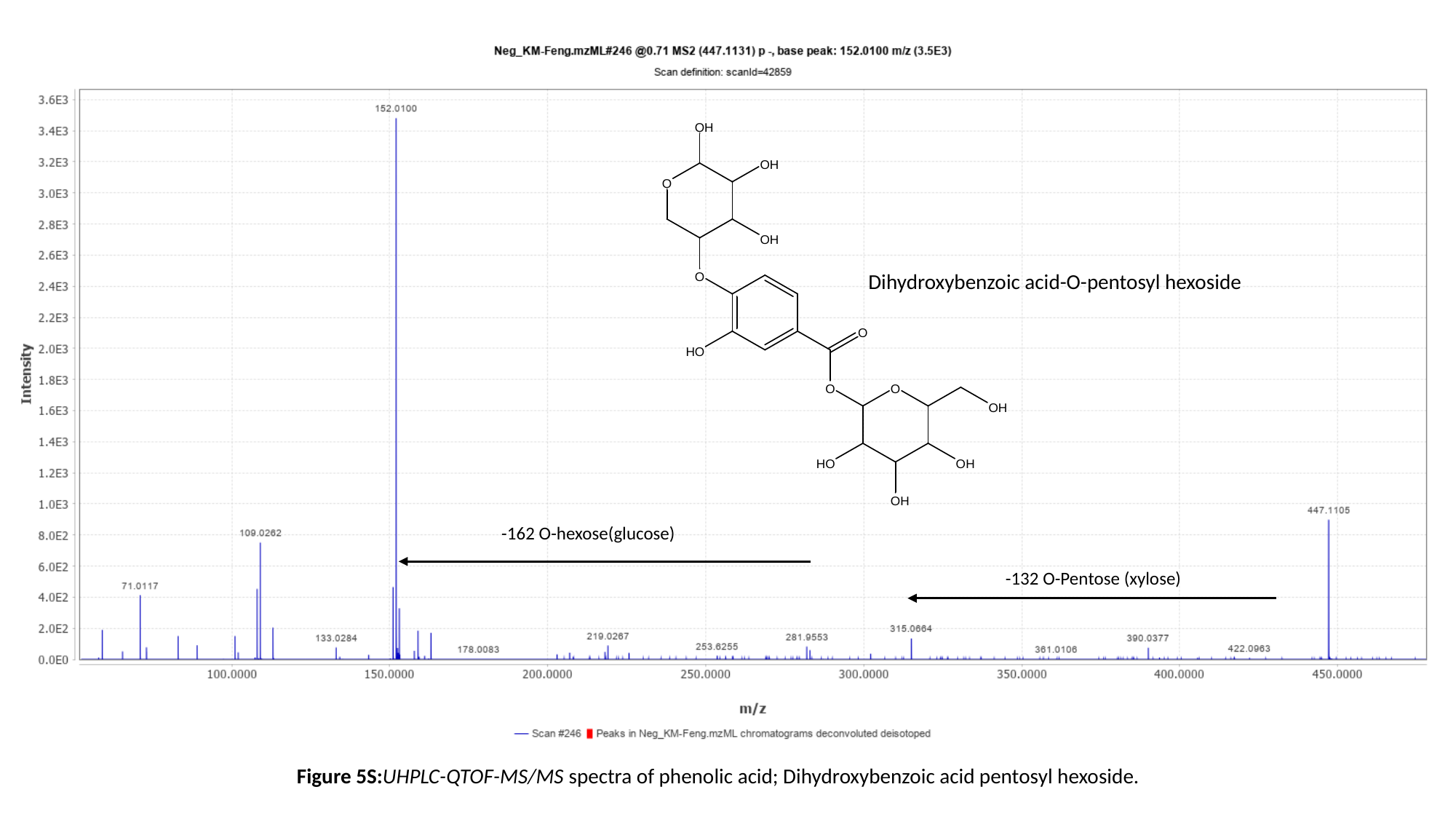

Dihydroxybenzoic acid-O-pentosyl hexoside
-162 O-hexose(glucose)
-132 O-Pentose (xylose)
Figure 5S:UHPLC-QTOF-MS/MS spectra of phenolic acid; Dihydroxybenzoic acid pentosyl hexoside.

## Slide 6
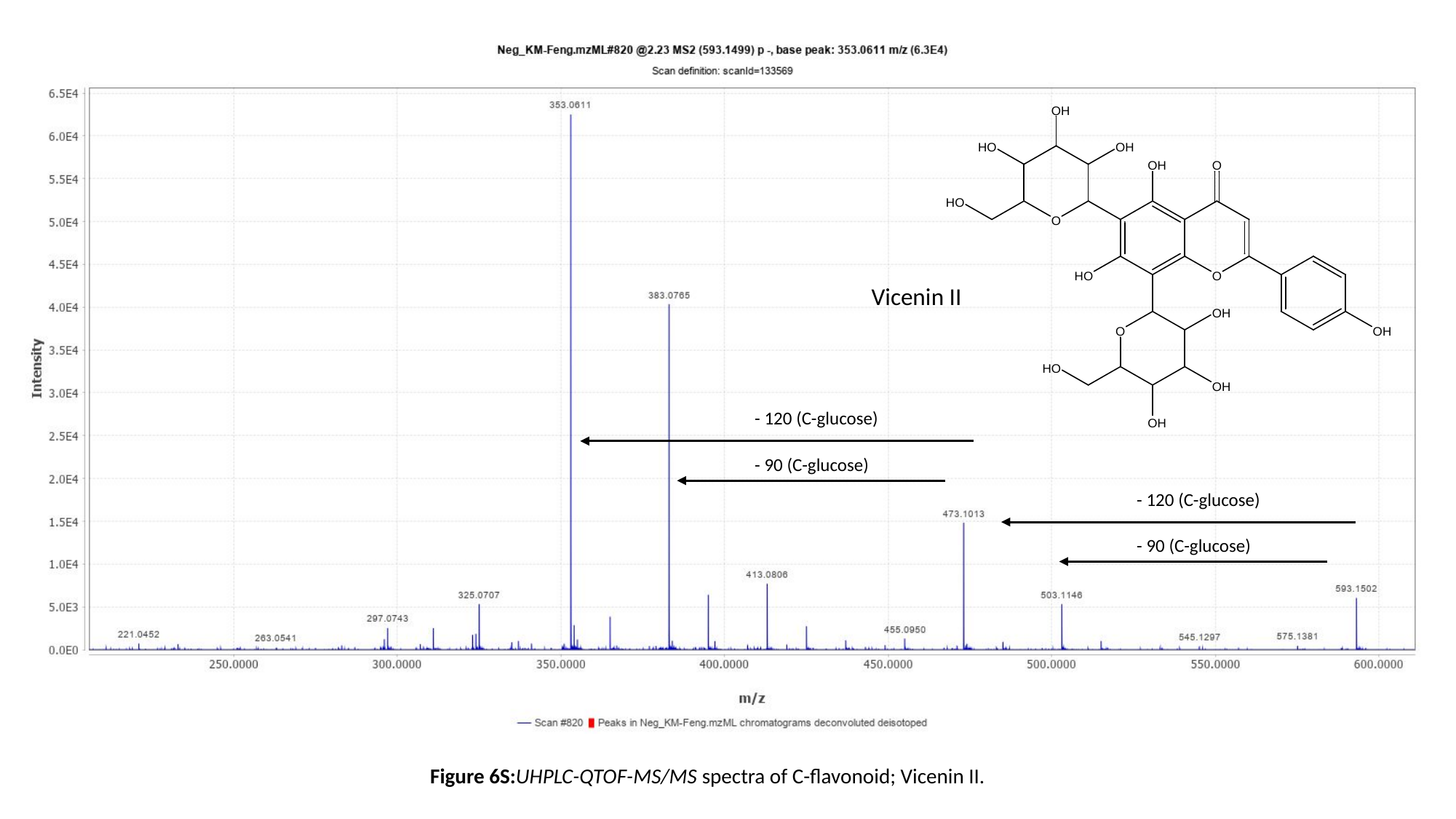

Vicenin II
- 120 (C-glucose)
- 90 (C-glucose)
- 120 (C-glucose)
- 90 (C-glucose)
Figure 6S:UHPLC-QTOF-MS/MS spectra of C-flavonoid; Vicenin II.

## Slide 7
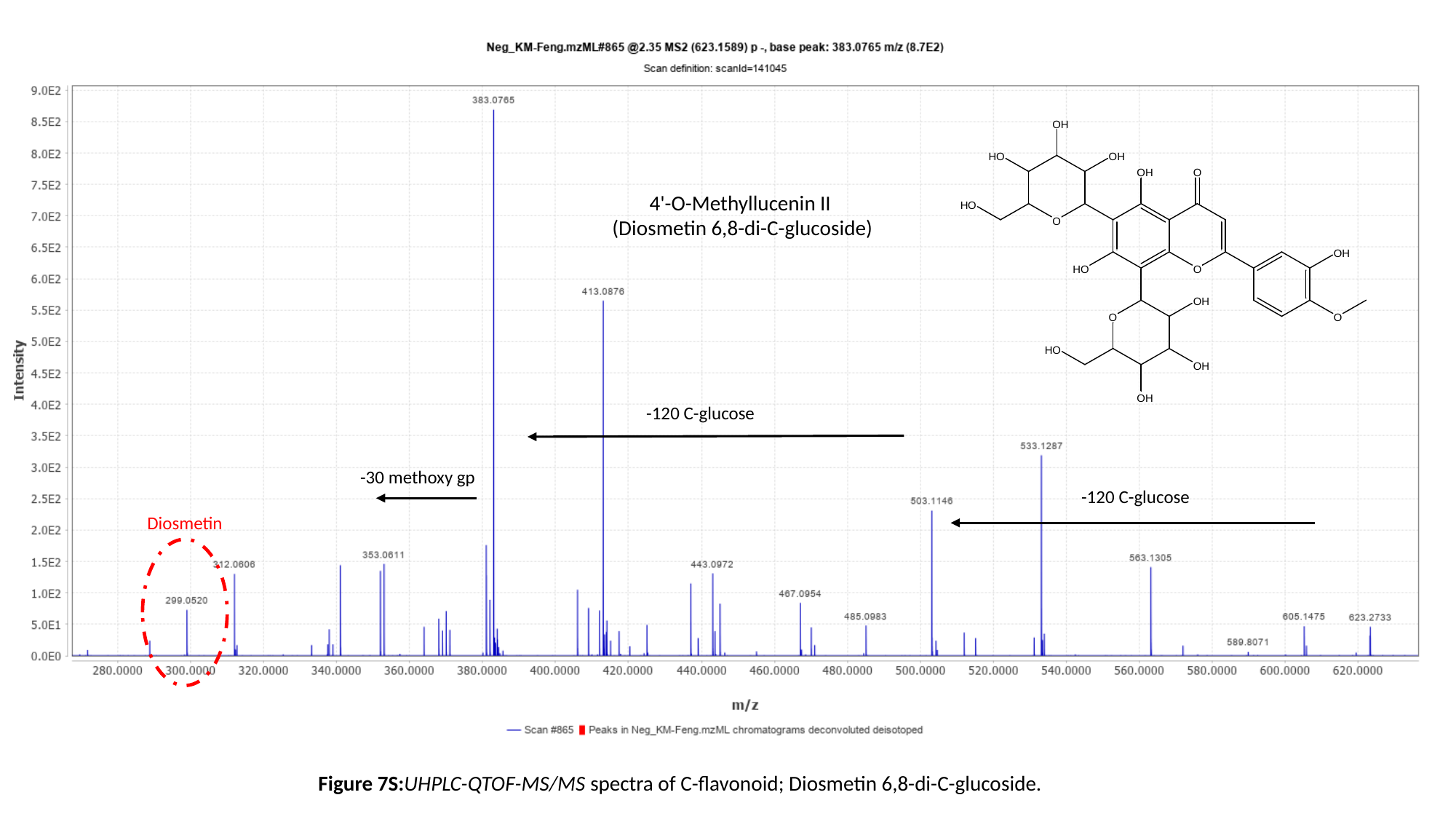

4'-O-Methyllucenin II
(Diosmetin 6,8-di-C-glucoside)
-120 C-glucose
-30 methoxy gp
-120 C-glucose
Diosmetin
Figure 7S:UHPLC-QTOF-MS/MS spectra of C-flavonoid; Diosmetin 6,8-di-C-glucoside.

## Slide 8
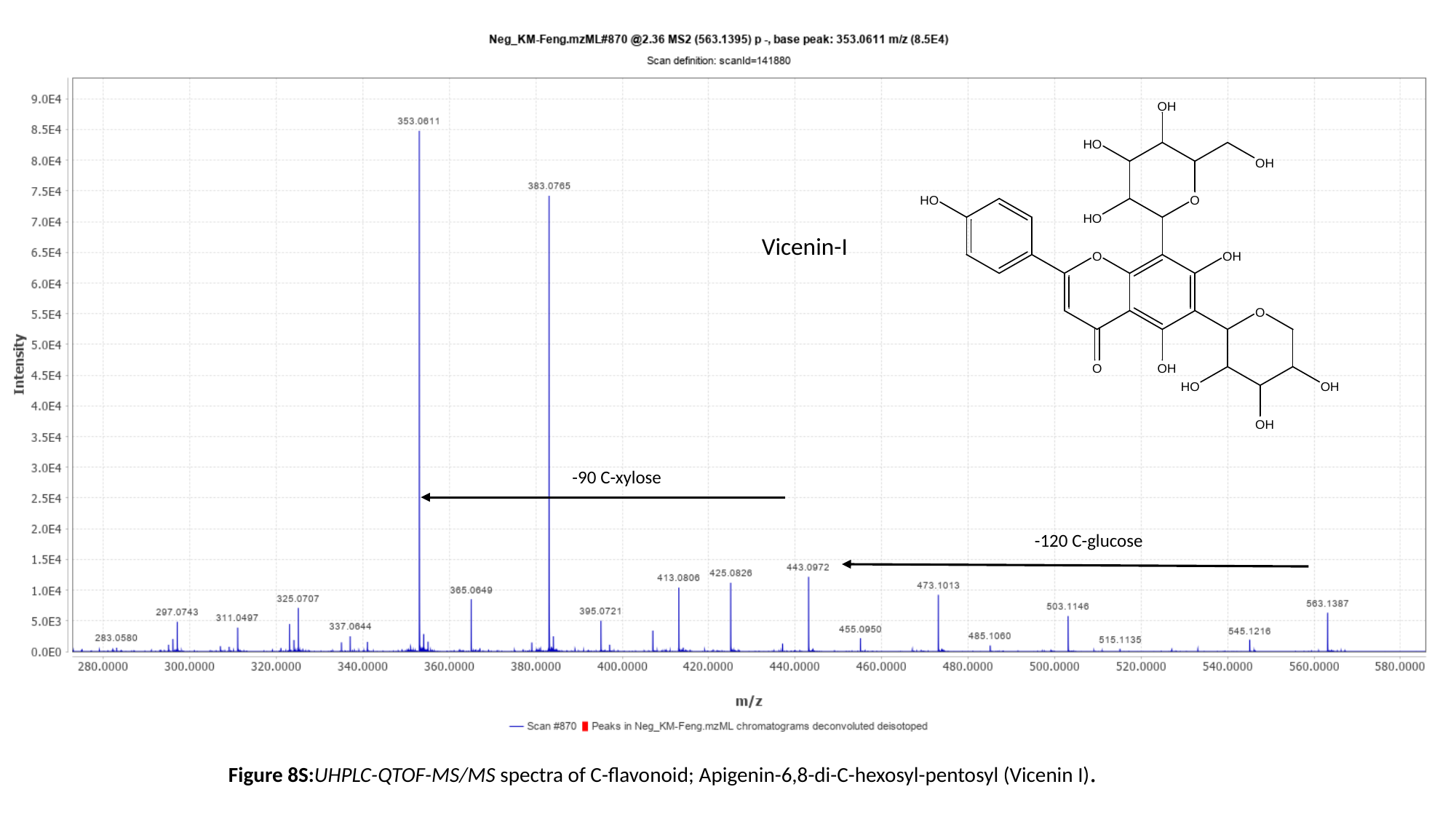

Vicenin-I
-90 C-xylose
-120 C-glucose
Vicenin-I
-90 C-xylose
-120 C-glucose
Figure 8S:UHPLC-QTOF-MS/MS spectra of C-flavonoid; Apigenin-6,8-di-C-hexosyl-pentosyl (Vicenin I).

## Slide 9
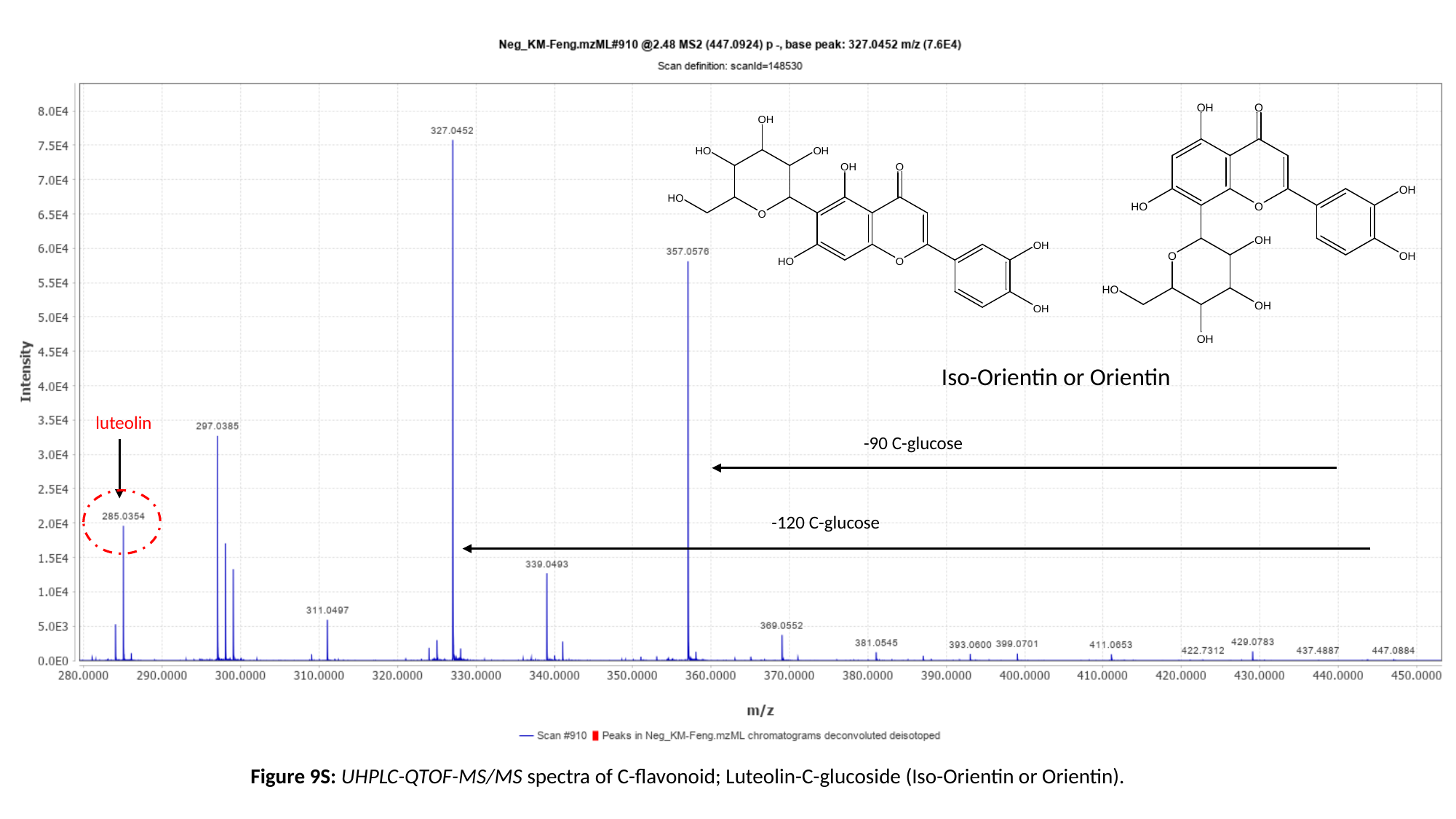

Iso-Orientin or Orientin
luteolin
-90 C-glucose
-120 C-glucose
Figure 9S: UHPLC-QTOF-MS/MS spectra of C-flavonoid; Luteolin-C-glucoside (Iso-Orientin or Orientin).

## Slide 10
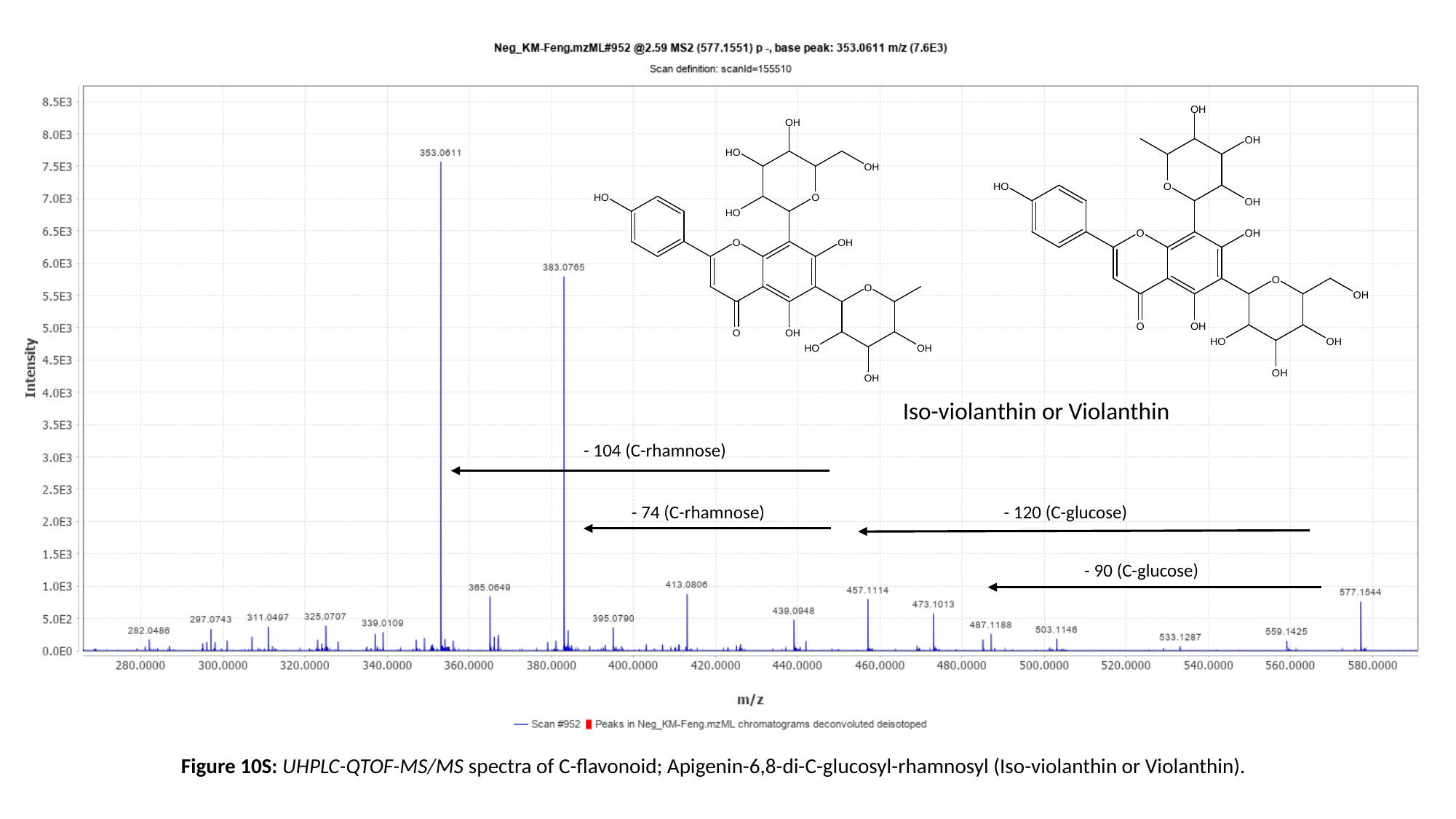

Iso-violanthin or Violanthin
- 104 (C-rhamnose)
- 74 (C-rhamnose)
- 120 (C-glucose)
- 90 (C-glucose)
Figure 10S: UHPLC-QTOF-MS/MS spectra of C-flavonoid; Apigenin-6,8-di-C-glucosyl-rhamnosyl (Iso-violanthin or Violanthin).

## Slide 11
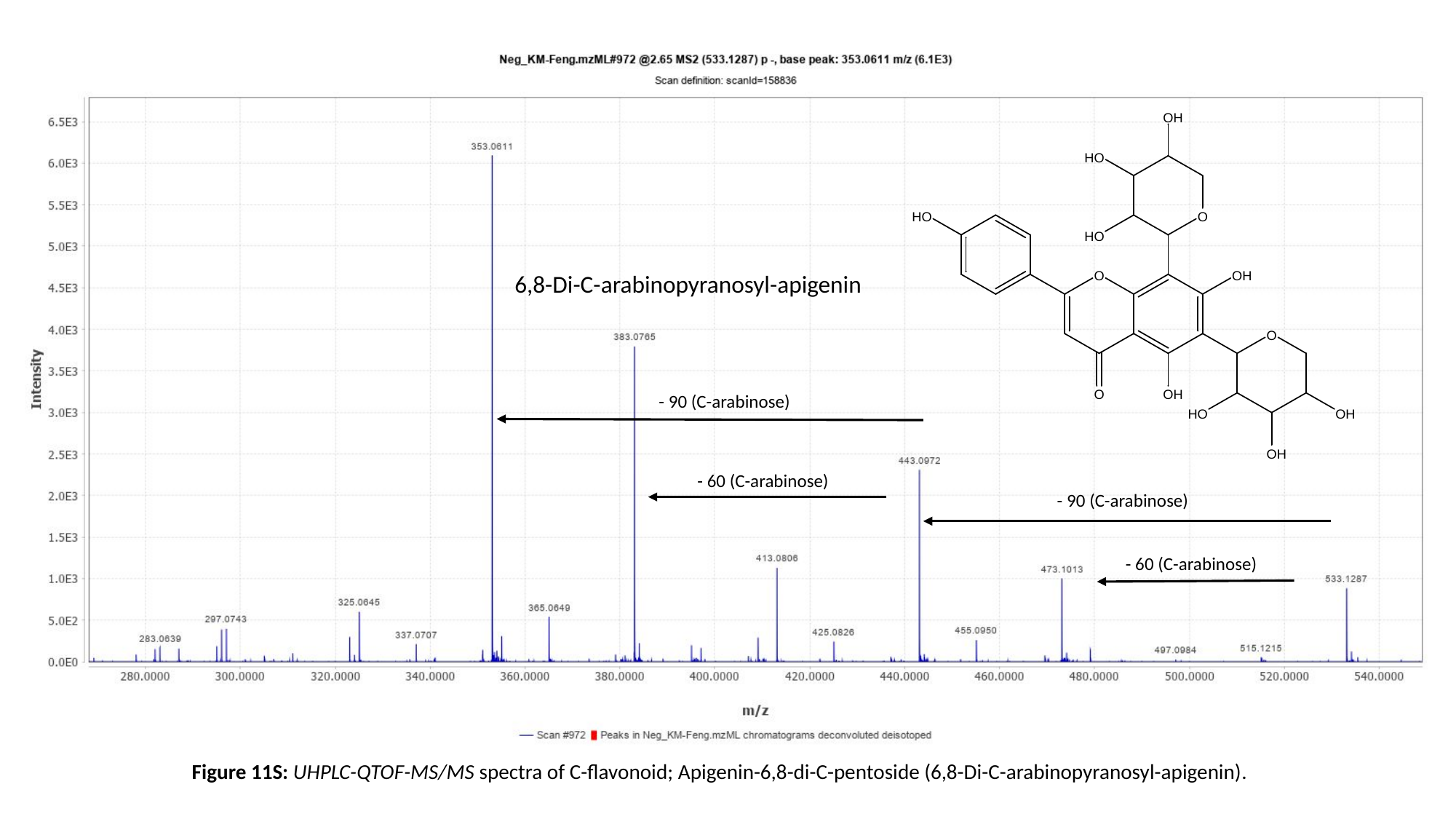

6,8-Di-C-arabinopyranosyl-apigenin
- 90 (C-arabinose)
- 60 (C-arabinose)
- 90 (C-arabinose)
- 60 (C-arabinose)
Figure 11S: UHPLC-QTOF-MS/MS spectra of C-flavonoid; Apigenin-6,8-di-C-pentoside (6,8-Di-C-arabinopyranosyl-apigenin).

## Slide 12
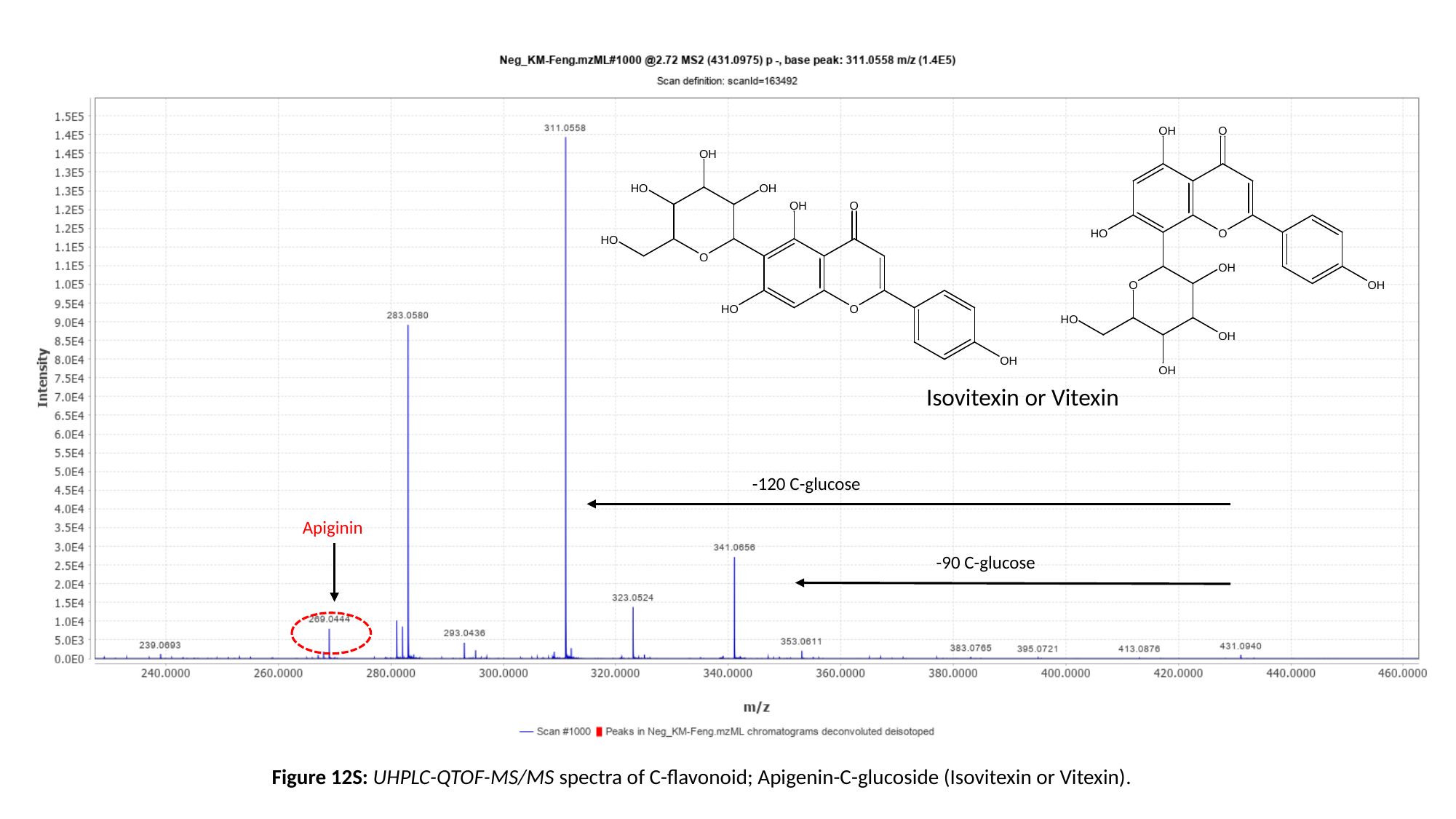

Isovitexin or Vitexin
-120 C-glucose
-90 C-glucose
Apiginin
Figure 12S: UHPLC-QTOF-MS/MS spectra of C-flavonoid; Apigenin-C-glucoside (Isovitexin or Vitexin).

## Slide 13
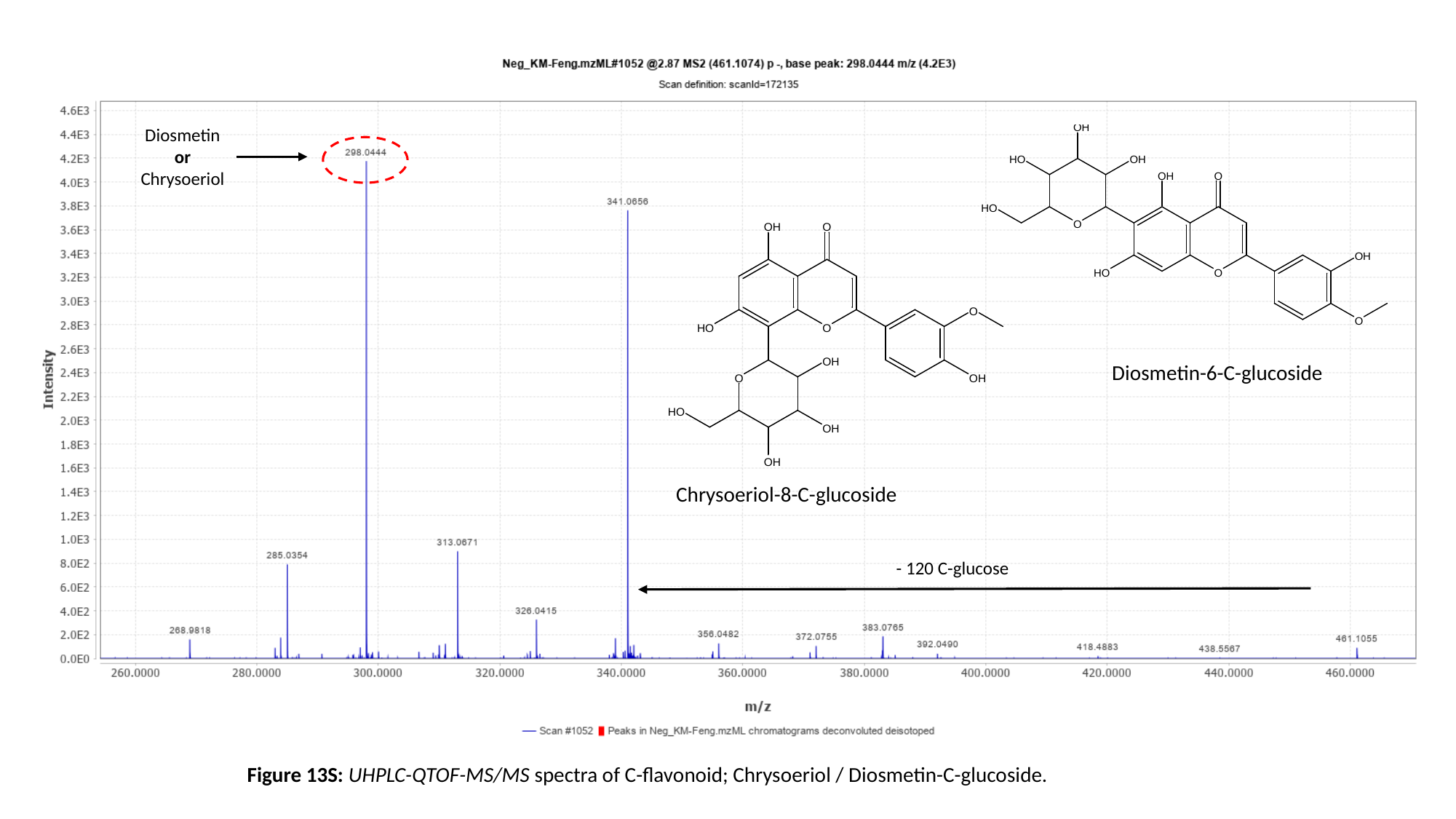

Diosmetin-6-C-glucoside
Chrysoeriol-8-C-glucoside
- 120 C-glucose
Diosmetin or Chrysoeriol
Figure 13S: UHPLC-QTOF-MS/MS spectra of C-flavonoid; Chrysoeriol / Diosmetin-C-glucoside.

## Slide 14
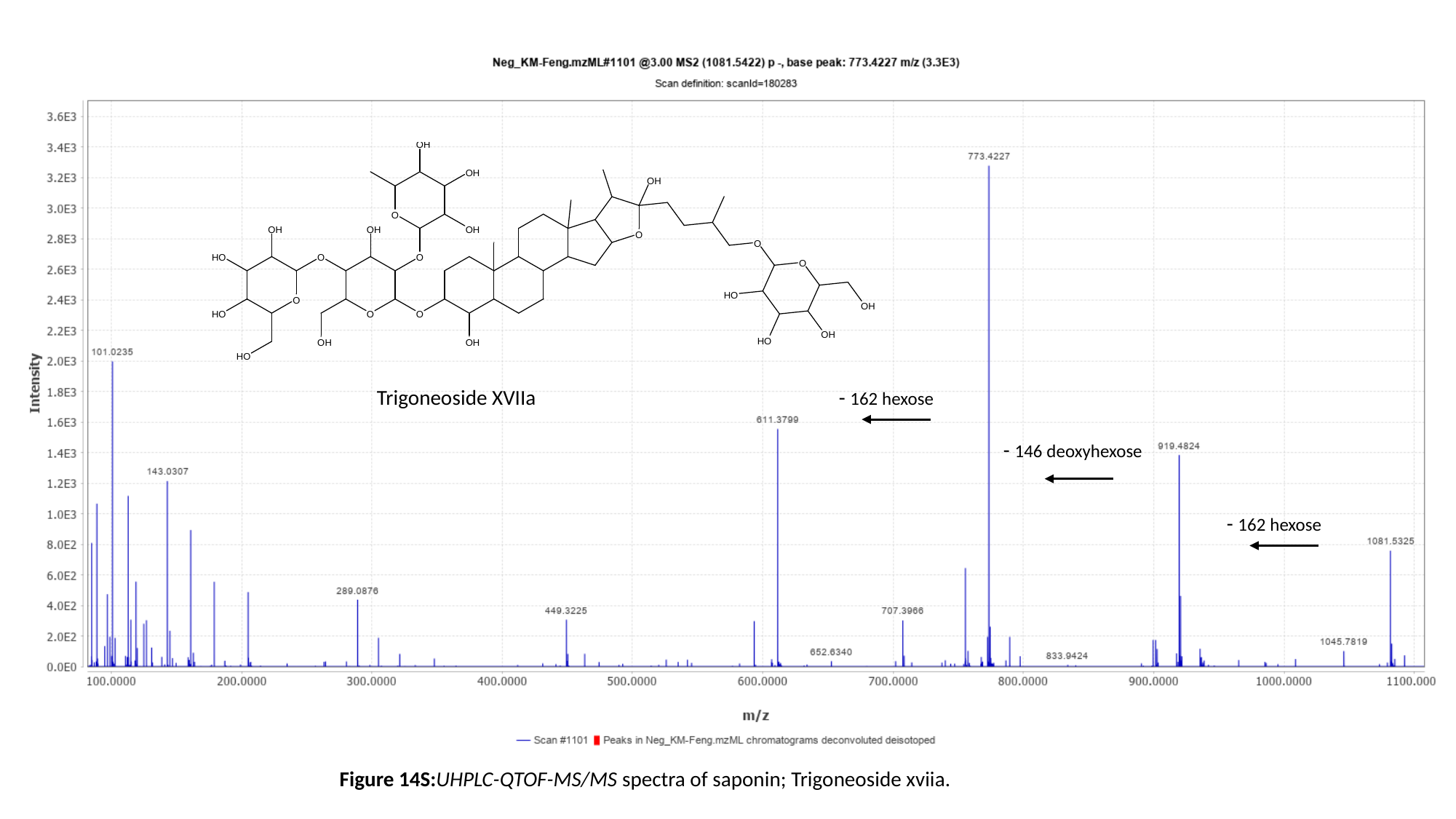

Trigoneoside XVIIa
- 162 hexose
- 146 deoxyhexose
- 162 hexose
Figure 14S:UHPLC-QTOF-MS/MS spectra of saponin; Trigoneoside xviia.

## Slide 15
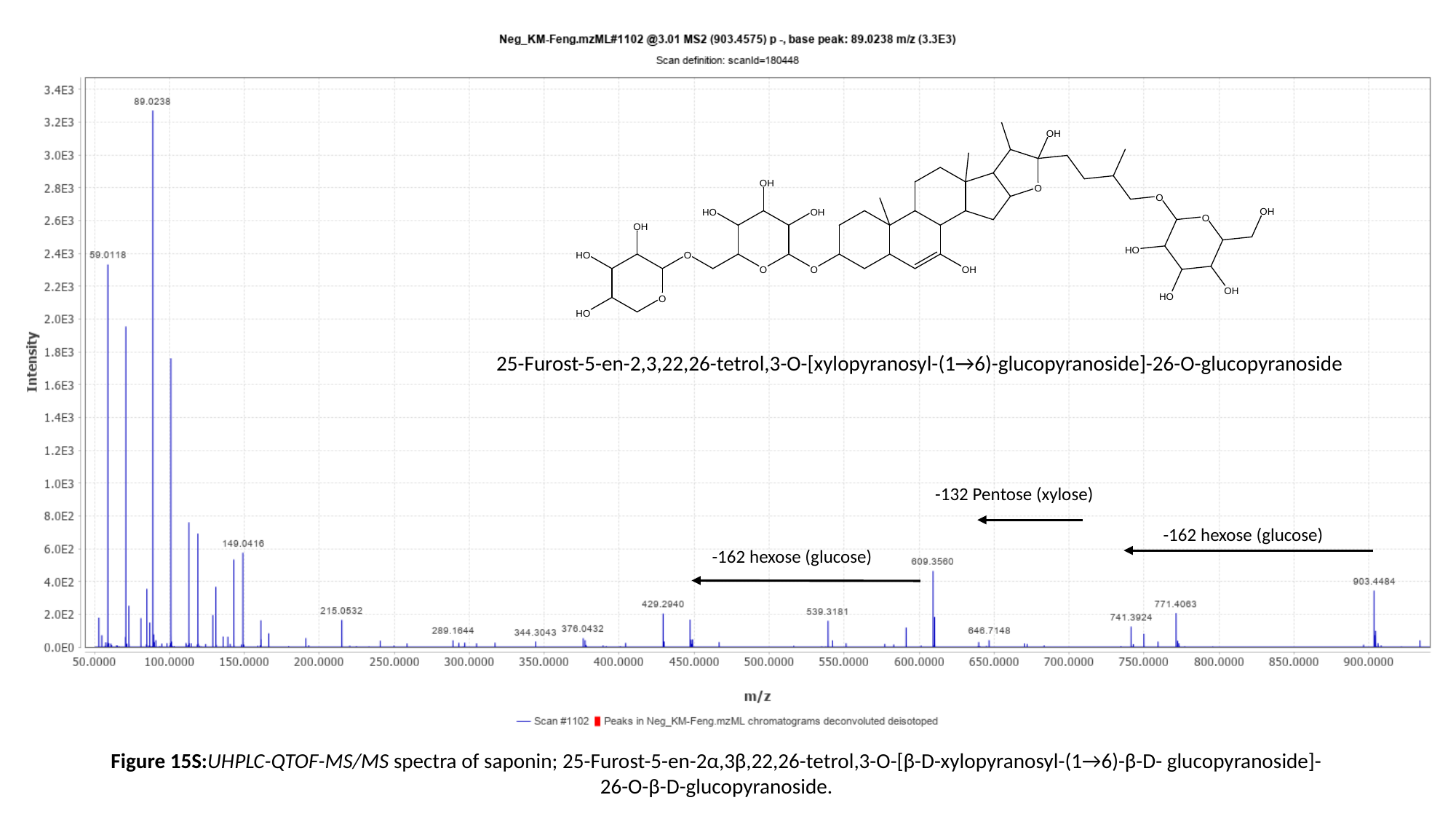

25-Furost-5-en-2,3,22,26-tetrol,3-O-[xylopyranosyl-(1→6)-glucopyranoside]-26-O-glucopyranoside
-132 Pentose (xylose)
-162 hexose (glucose)
-162 hexose (glucose)
Figure 15S:UHPLC-QTOF-MS/MS spectra of saponin; 25-Furost-5-en-2α,3β,22,26-tetrol,3-O-[β-D-xylopyranosyl-(1→6)-β-D- glucopyranoside]-26-O-β-D-glucopyranoside.

## Slide 16
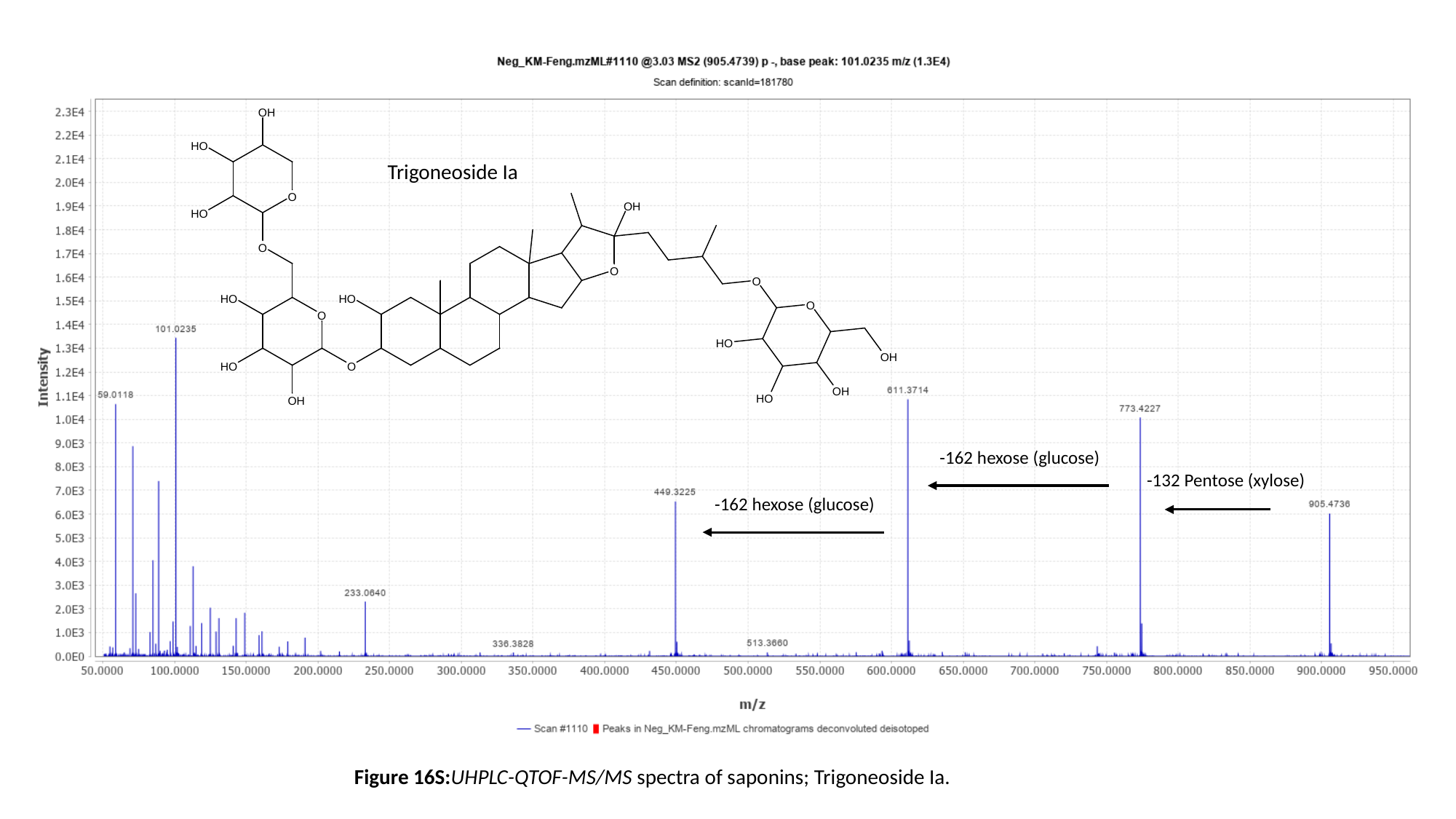

Trigoneoside Ia
-162 hexose (glucose)
-132 Pentose (xylose)
-162 hexose (glucose)
Figure 16S:UHPLC-QTOF-MS/MS spectra of saponins; Trigoneoside Ia.

## Slide 17
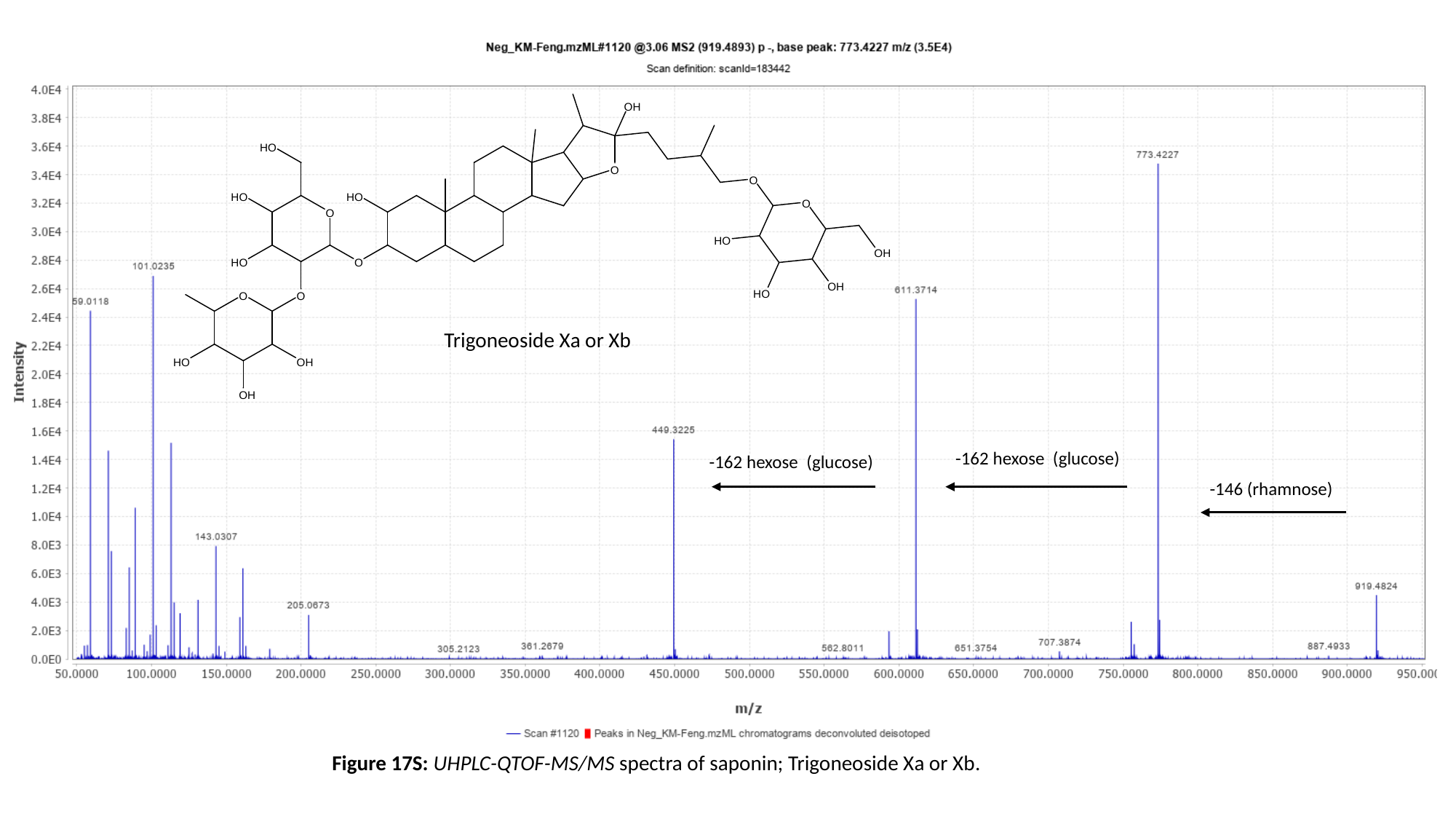

Trigoneoside Xa or Xb
-162 hexose (glucose)
-162 hexose (glucose)
-146 (rhamnose)
Figure 17S: UHPLC-QTOF-MS/MS spectra of saponin; Trigoneoside Xa or Xb.

## Slide 18
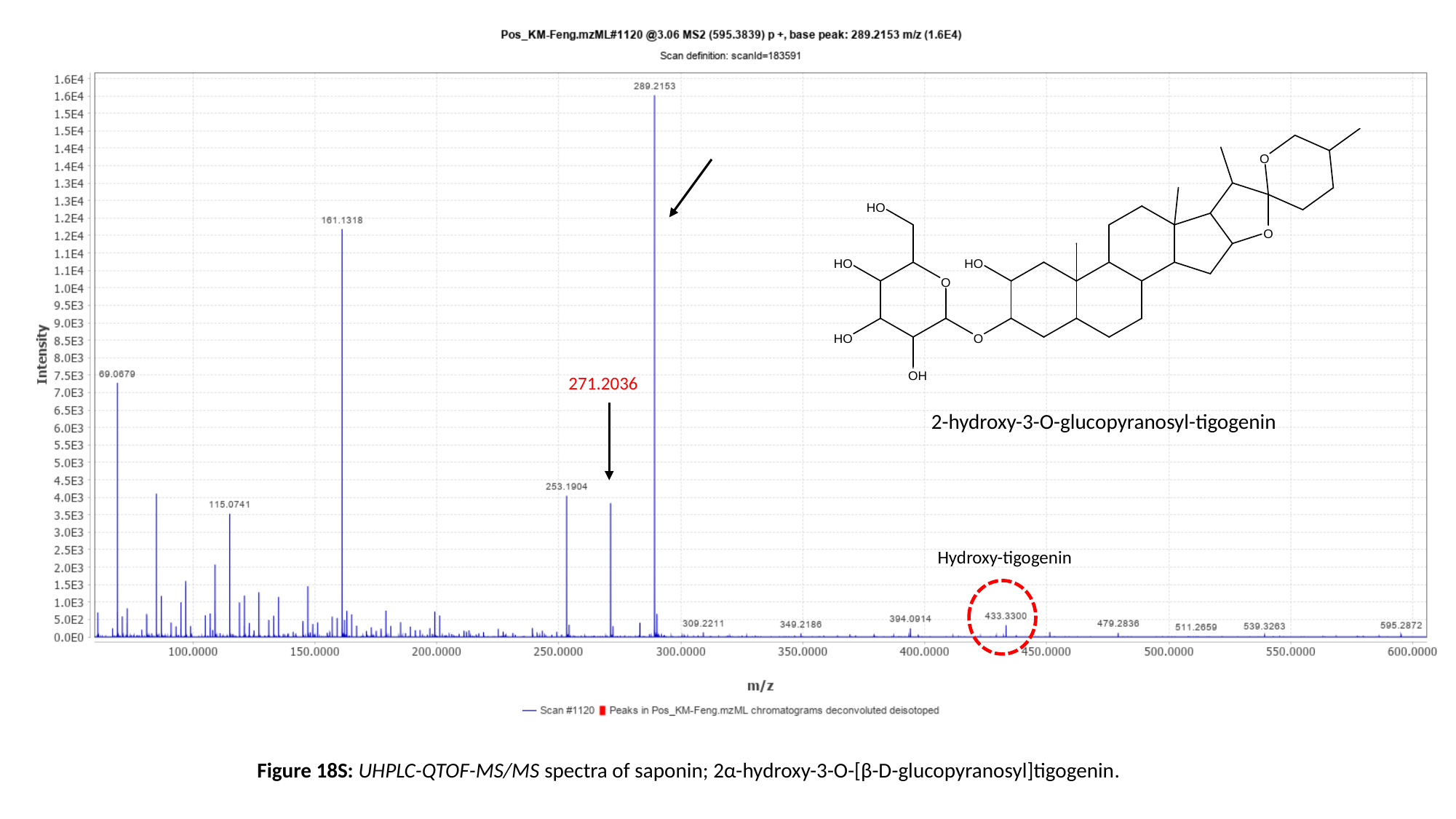

2-hydroxy-3-O-glucopyranosyl-tigogenin
271.2036
Hydroxy-tigogenin
Figure 18S: UHPLC-QTOF-MS/MS spectra of saponin; 2α-hydroxy-3-O-[β-D-glucopyranosyl]tigogenin.

## Slide 19
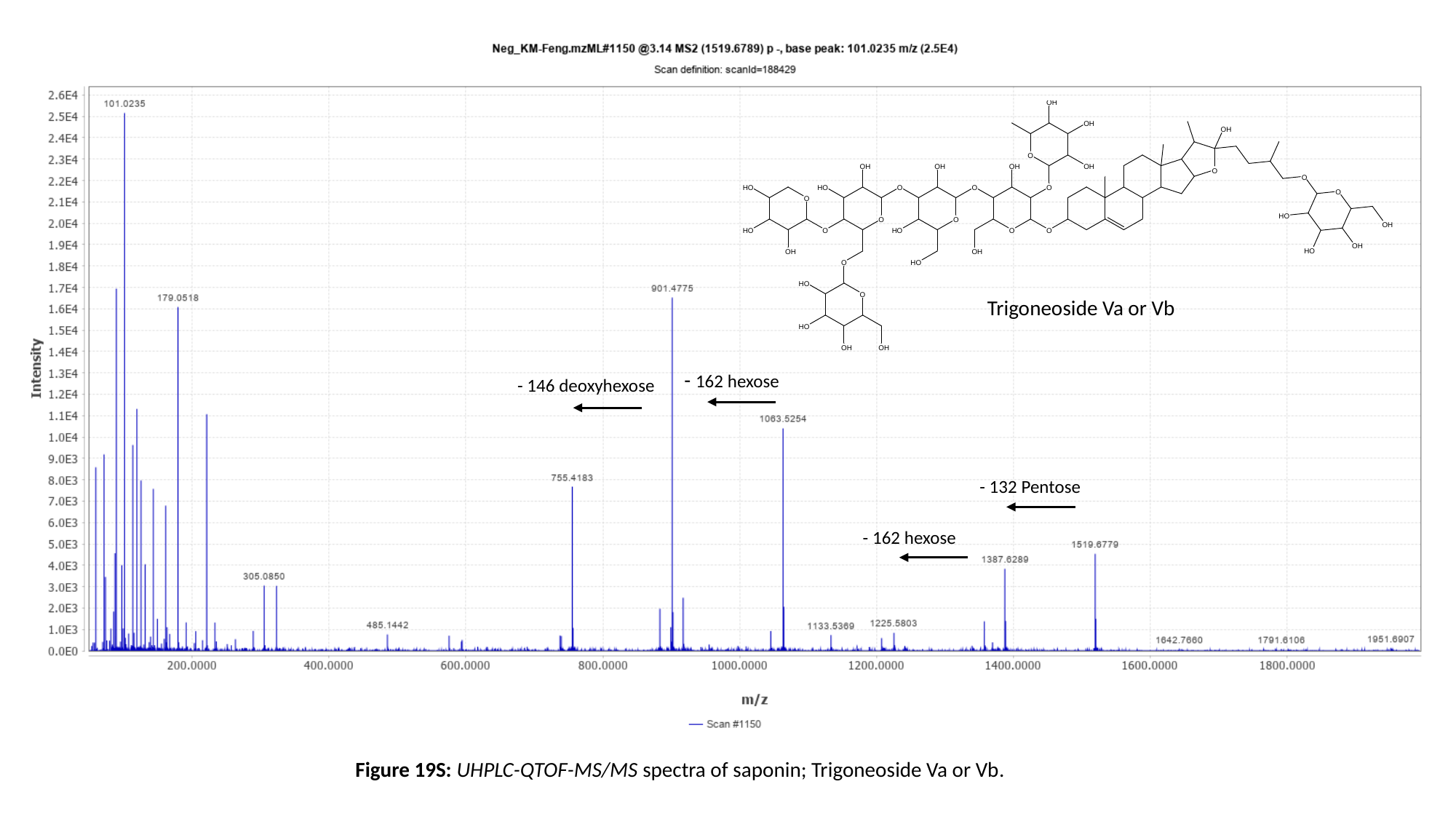

- 162 hexose
- 146 deoxyhexose
- 132 Pentose
- 162 hexose
Trigoneoside Va or Vb
Trigoneoside Va &Vb
Figure 19S: UHPLC-QTOF-MS/MS spectra of saponin; Trigoneoside Va or Vb.

## Slide 20
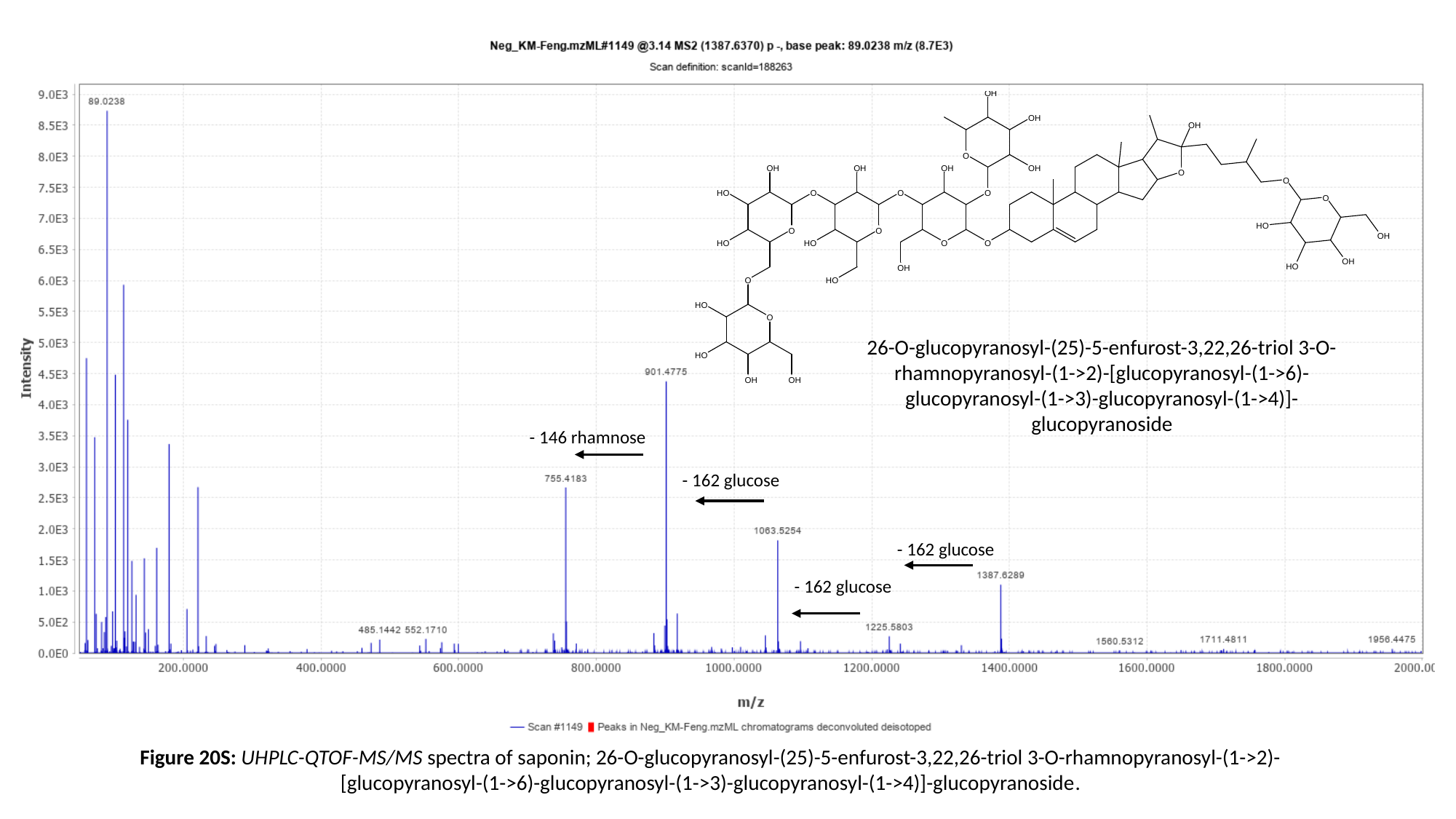

26-O-glucopyranosyl-(25)-5-enfurost-3,22,26-triol 3-O-rhamnopyranosyl-(1->2)-[glucopyranosyl-(1->6)-glucopyranosyl-(1->3)-glucopyranosyl-(1->4)]-glucopyranoside
- 146 rhamnose
- 162 glucose
- 162 glucose
- 162 glucose
Figure 20S: UHPLC-QTOF-MS/MS spectra of saponin; 26-O-glucopyranosyl-(25)-5-enfurost-3,22,26-triol 3-O-rhamnopyranosyl-(1->2)-[glucopyranosyl-(1->6)-glucopyranosyl-(1->3)-glucopyranosyl-(1->4)]-glucopyranoside.

## Slide 21
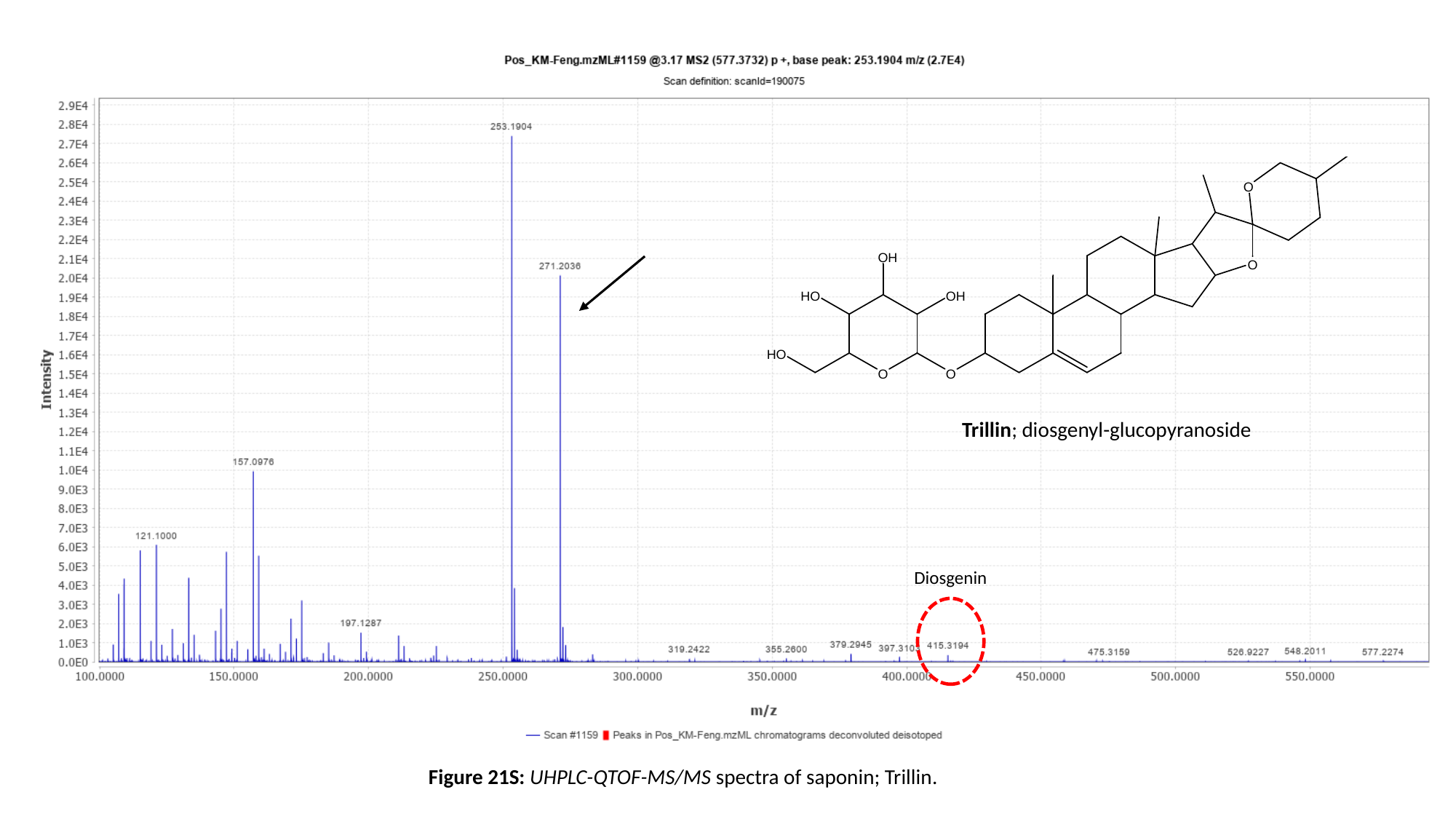

Trillin; diosgenyl-glucopyranoside
Diosgenin
Figure 21S: UHPLC-QTOF-MS/MS spectra of saponin; Trillin.

## Slide 22
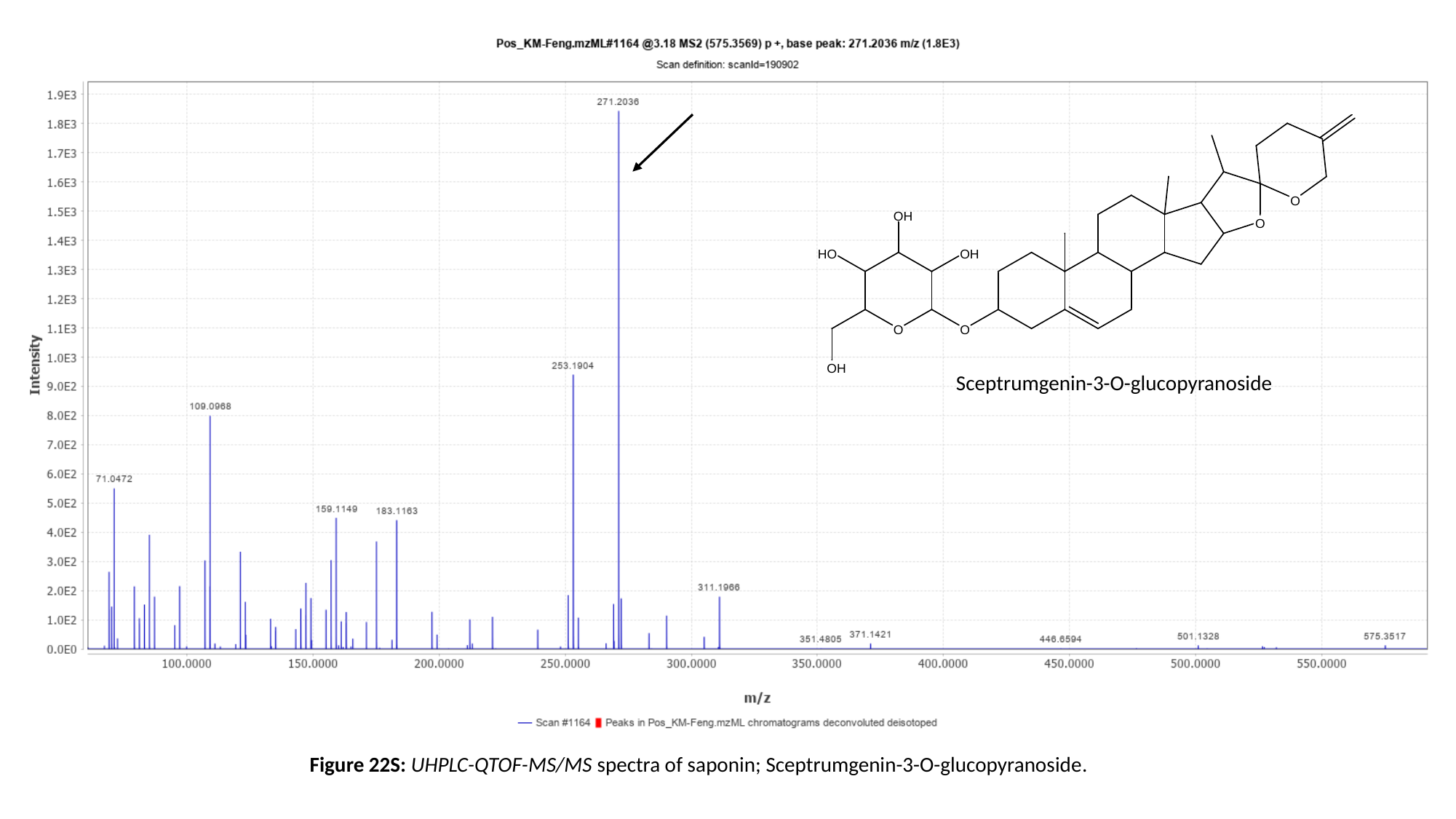

Sceptrumgenin-3-O-glucopyranoside
Figure 22S: UHPLC-QTOF-MS/MS spectra of saponin; Sceptrumgenin-3-O-glucopyranoside.

## Slide 23
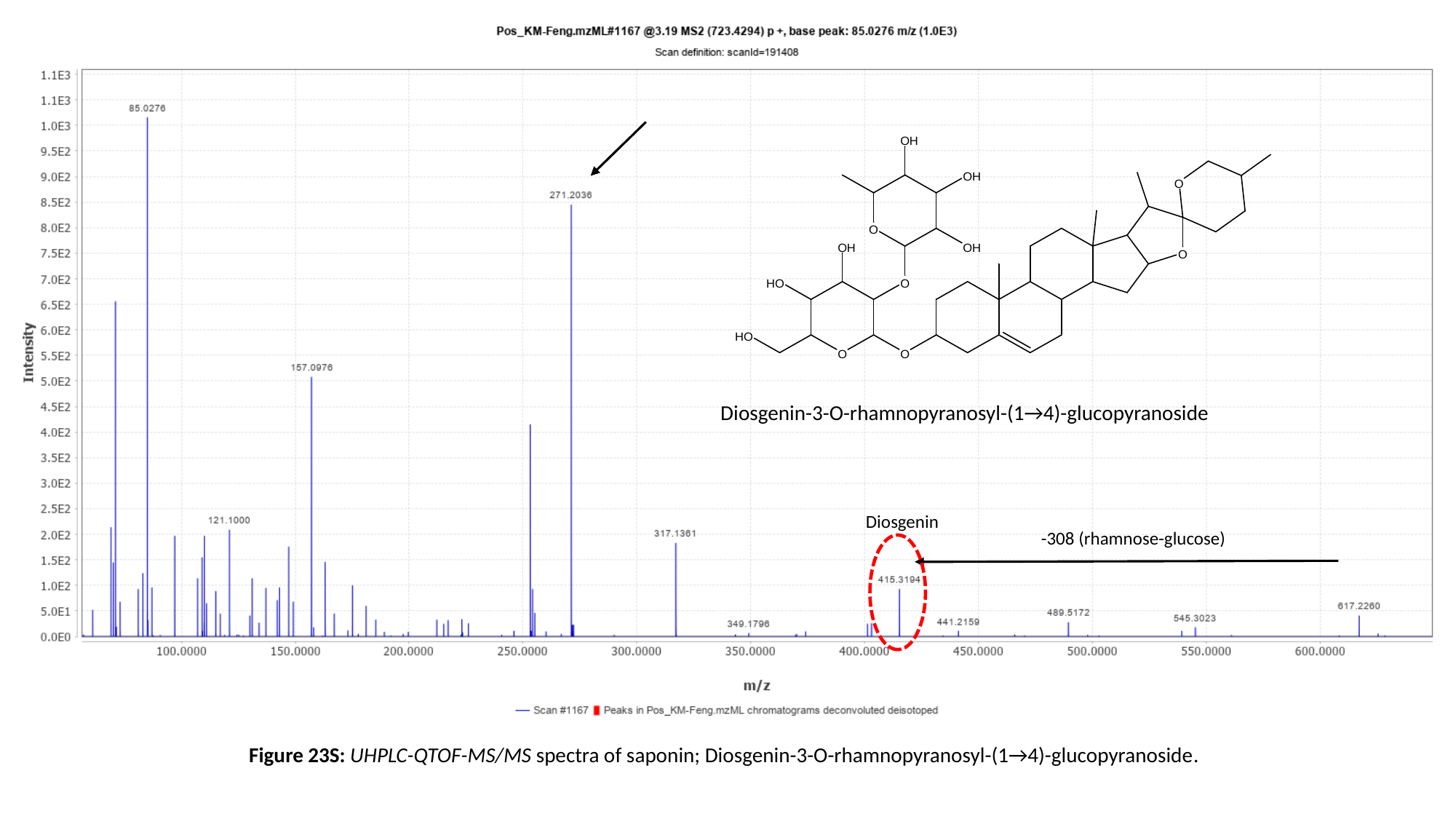

Diosgenin-3-O-rhamnopyranosyl-(1→4)-glucopyranoside
-308 (rhamnose-glucose)
Diosgenin
Figure 23S: UHPLC-QTOF-MS/MS spectra of saponin; Diosgenin-3-O-rhamnopyranosyl-(1→4)-glucopyranoside.

## Slide 24
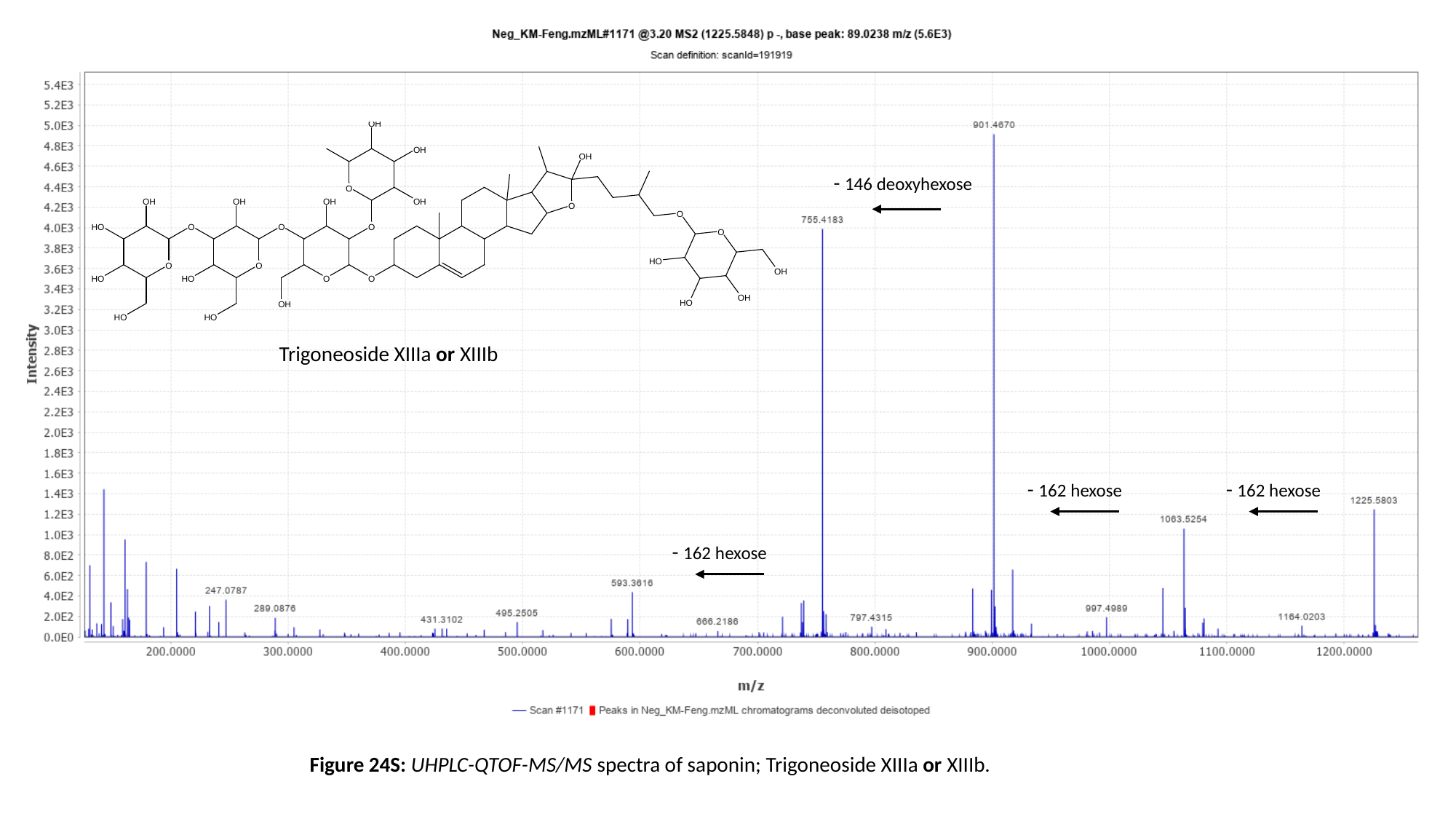

Trigoneoside XIIIa or XIIIb
- 146 deoxyhexose
- 162 hexose
- 162 hexose
- 162 hexose
Figure 24S: UHPLC-QTOF-MS/MS spectra of saponin; Trigoneoside XIIIa or XIIIb.

## Slide 25
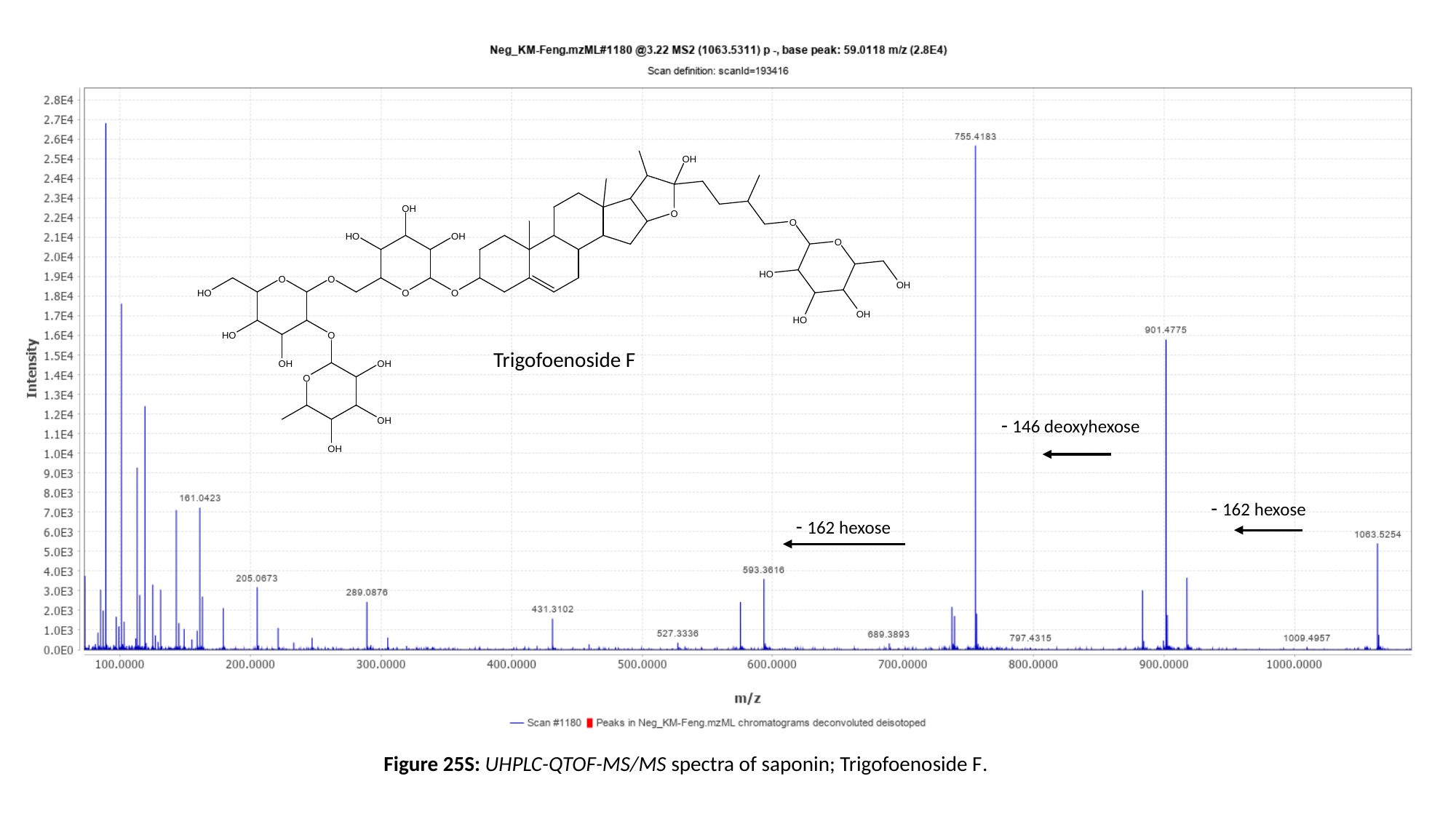

Trigofoenoside F
- 146 deoxyhexose
- 162 hexose
- 162 hexose
Figure 25S: UHPLC-QTOF-MS/MS spectra of saponin; Trigofoenoside F.

## Slide 26
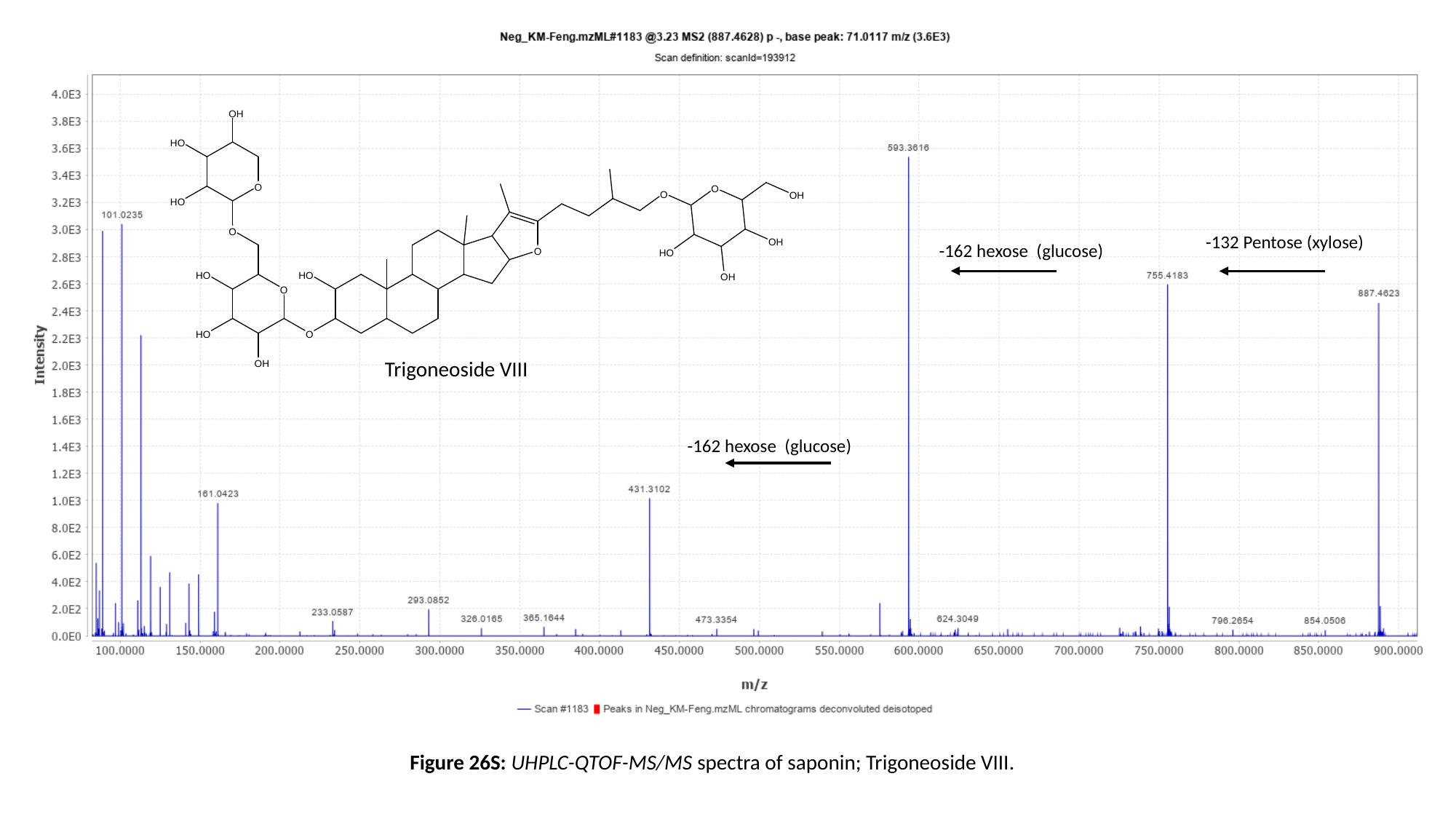

-132 Pentose (xylose)
-162 hexose (glucose)
Trigoneoside VIII
-162 hexose (glucose)
Figure 26S: UHPLC-QTOF-MS/MS spectra of saponin; Trigoneoside VIII.

## Slide 27
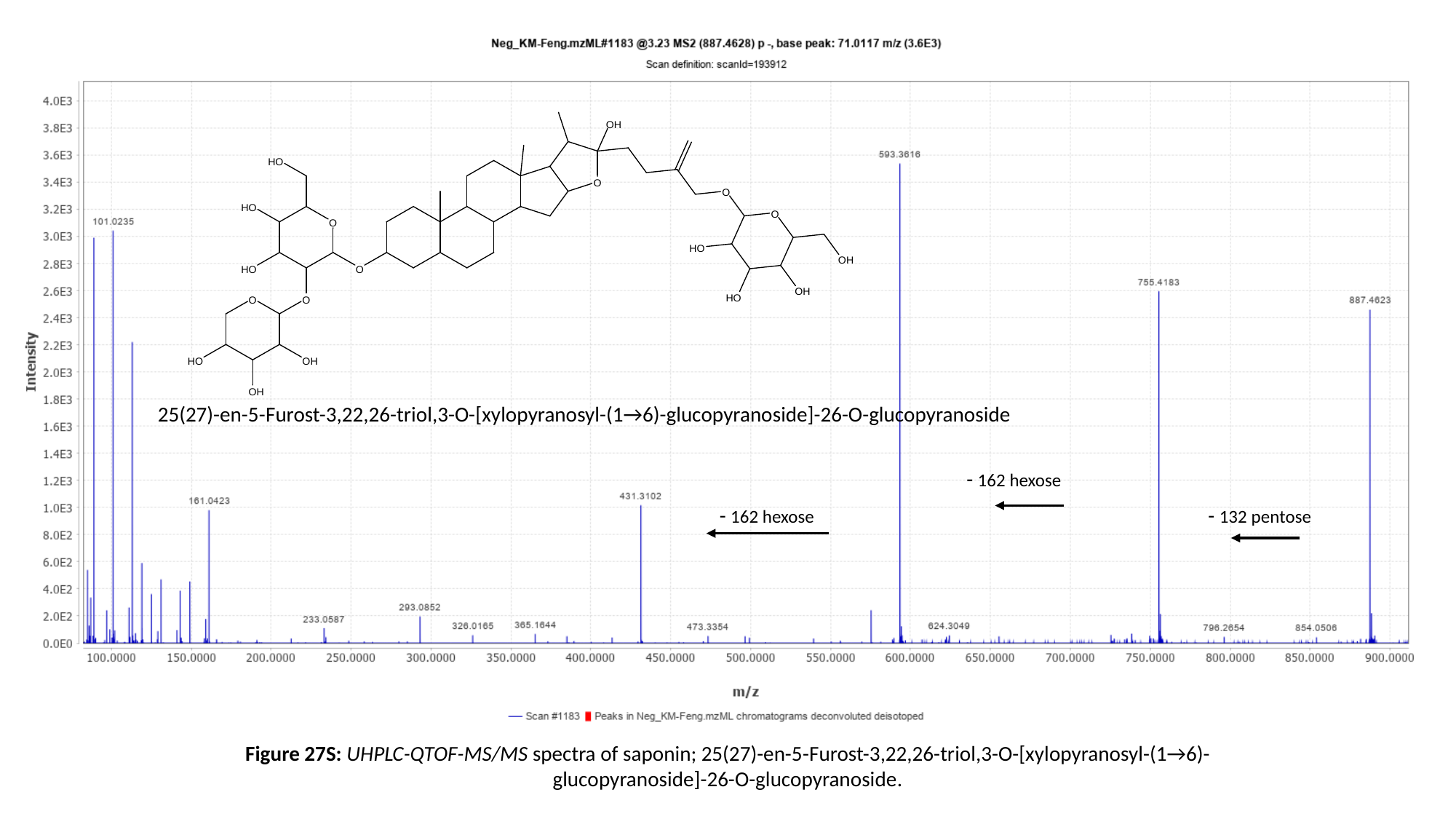

25(27)-en-5-Furost-3,22,26-triol,3-O-[xylopyranosyl-(1→6)-glucopyranoside]-26-O-glucopyranoside
- 162 hexose
- 162 hexose
- 132 pentose
Figure 27S: UHPLC-QTOF-MS/MS spectra of saponin; 25(27)-en-5-Furost-3,22,26-triol,3-O-[xylopyranosyl-(1→6)-glucopyranoside]-26-O-glucopyranoside.

## Slide 28
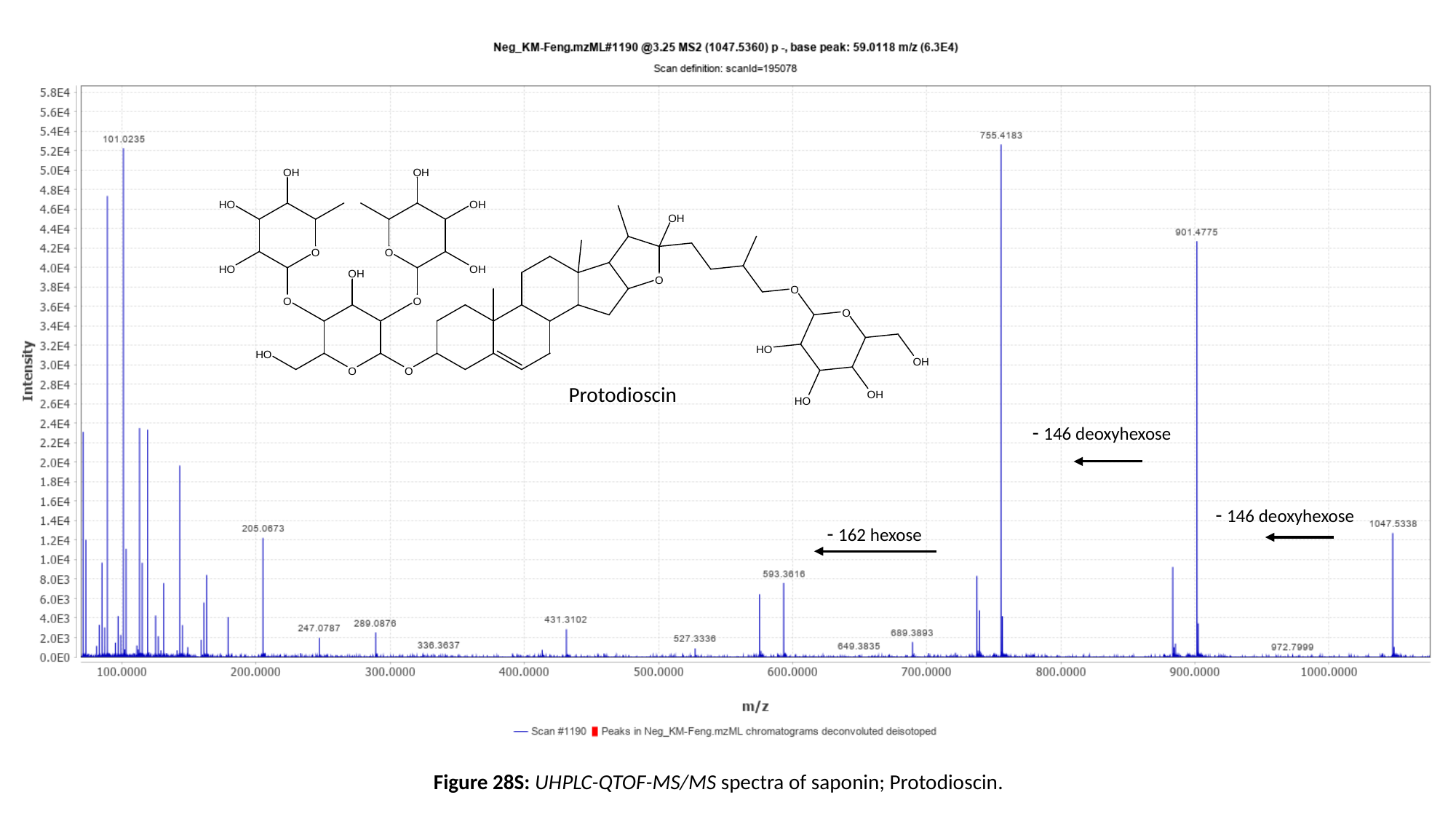

Protodioscin
- 146 deoxyhexose
- 146 deoxyhexose
- 162 hexose
Figure 28S: UHPLC-QTOF-MS/MS spectra of saponin; Protodioscin.

## Slide 29
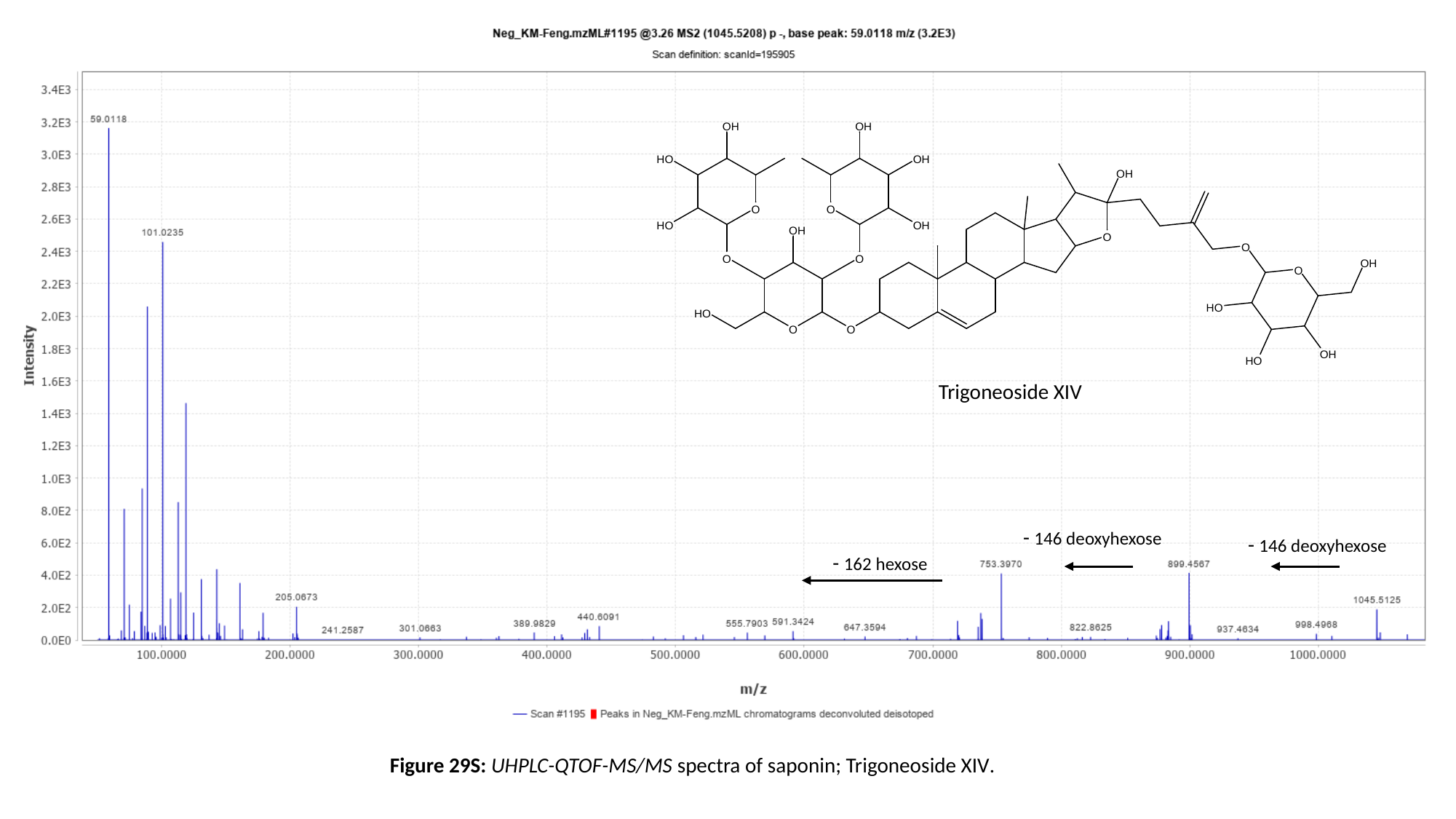

Trigoneoside XIV
- 146 deoxyhexose
- 146 deoxyhexose
- 162 hexose
Figure 29S: UHPLC-QTOF-MS/MS spectra of saponin; Trigoneoside XIV.

## Slide 30
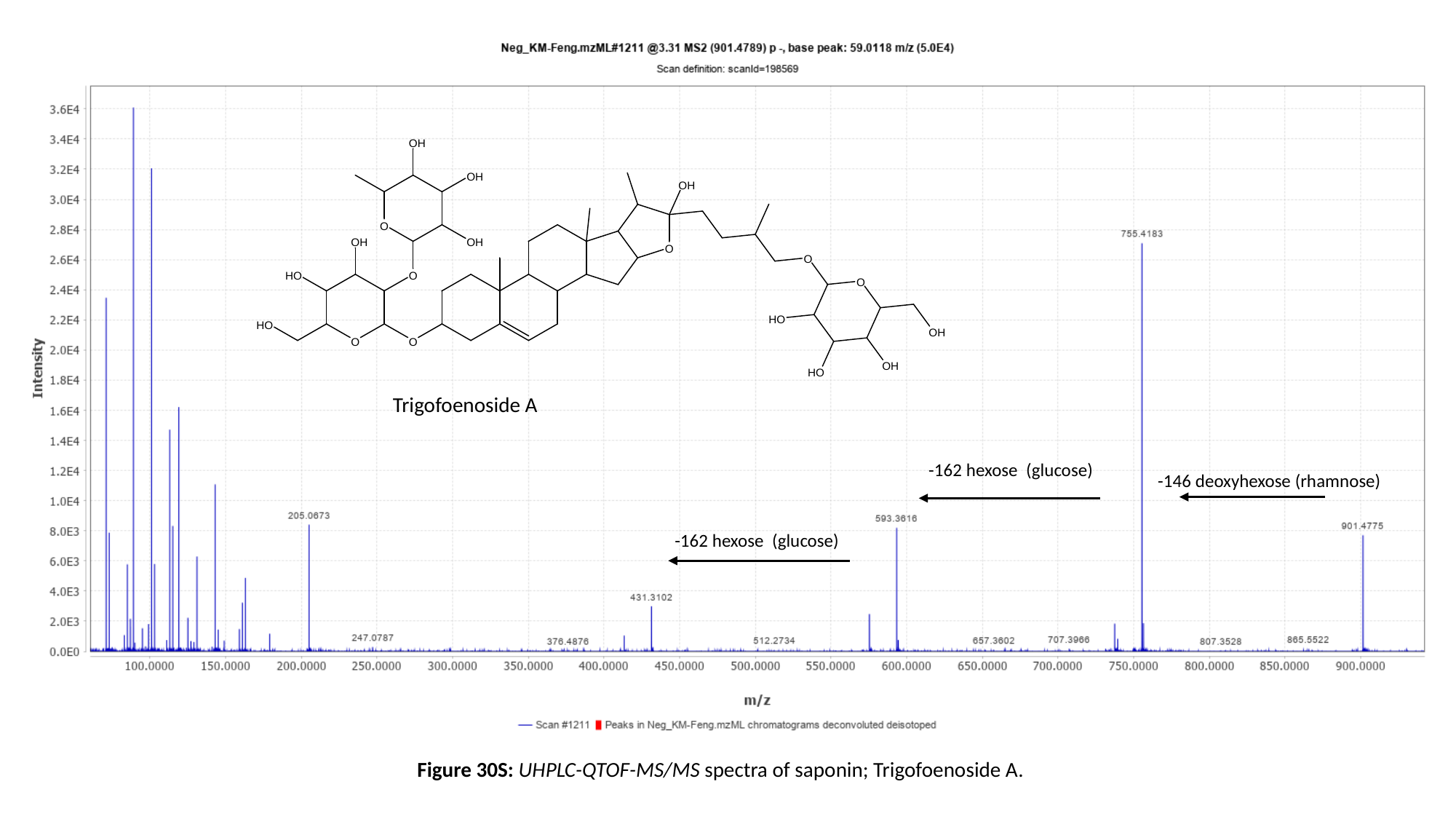

Trigofoenoside A
-162 hexose (glucose)
-146 deoxyhexose (rhamnose)
-162 hexose (glucose)
Figure 30S: UHPLC-QTOF-MS/MS spectra of saponin; Trigofoenoside A.

## Slide 31
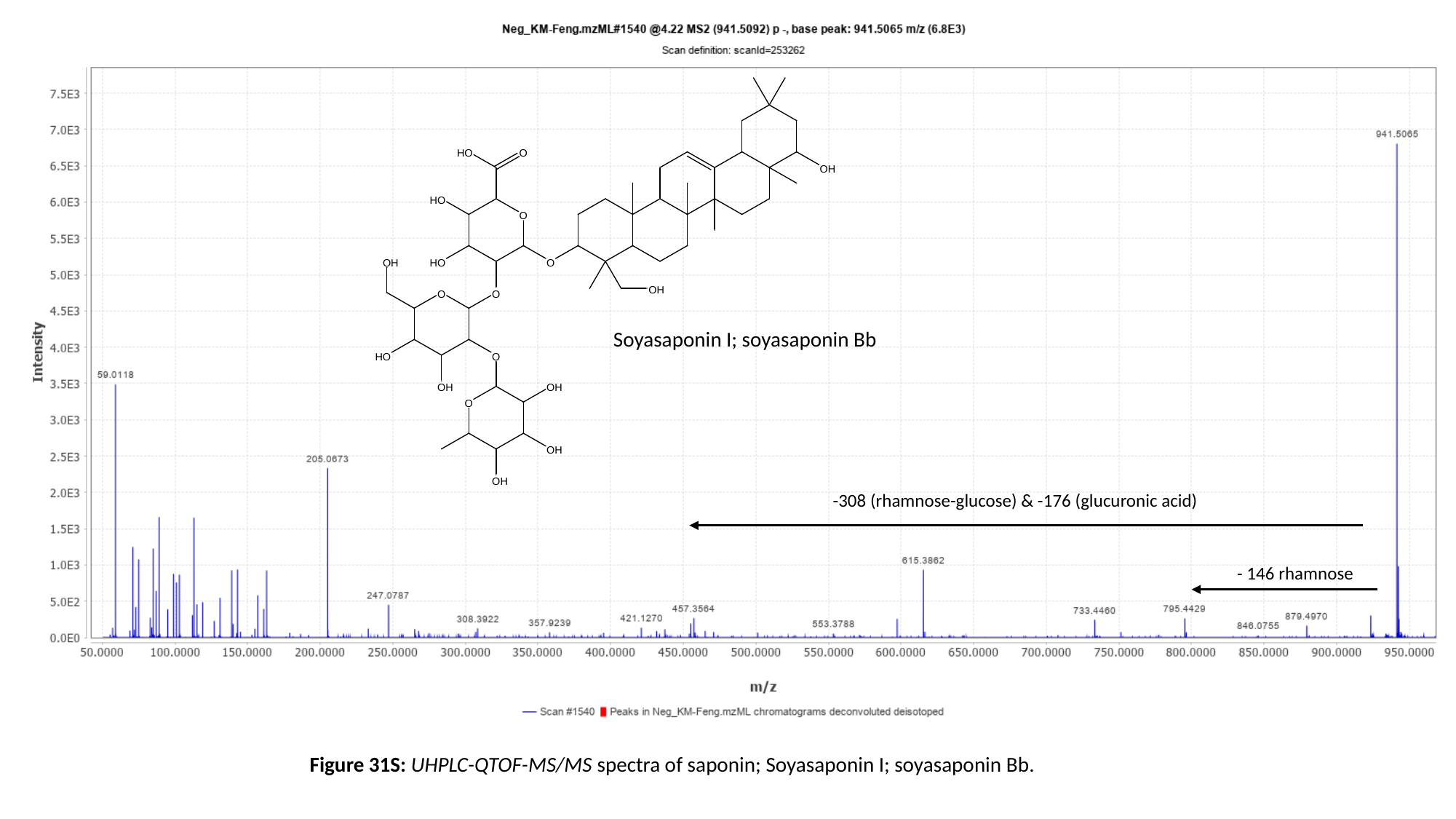

Soyasaponin I; soyasaponin Bb
-308 (rhamnose-glucose) & -176 (glucuronic acid)
- 146 rhamnose
Figure 31S: UHPLC-QTOF-MS/MS spectra of saponin; Soyasaponin I; soyasaponin Bb.

## Slide 32
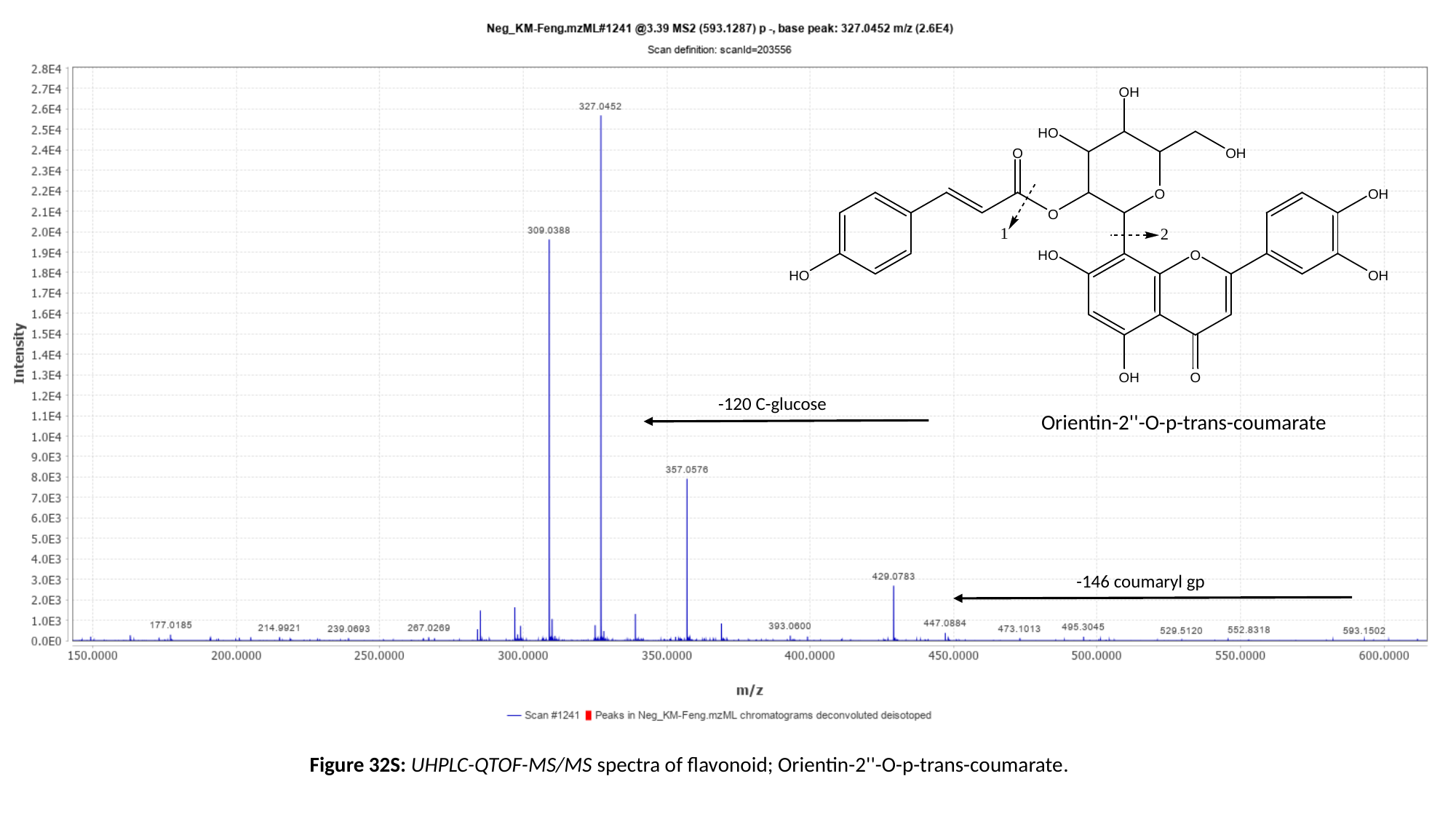

-120 C-glucose
Orientin-2''-O-p-trans-coumarate
-146 coumaryl gp
Figure 32S: UHPLC-QTOF-MS/MS spectra of flavonoid; Orientin-2''-O-p-trans-coumarate.

## Slide 33
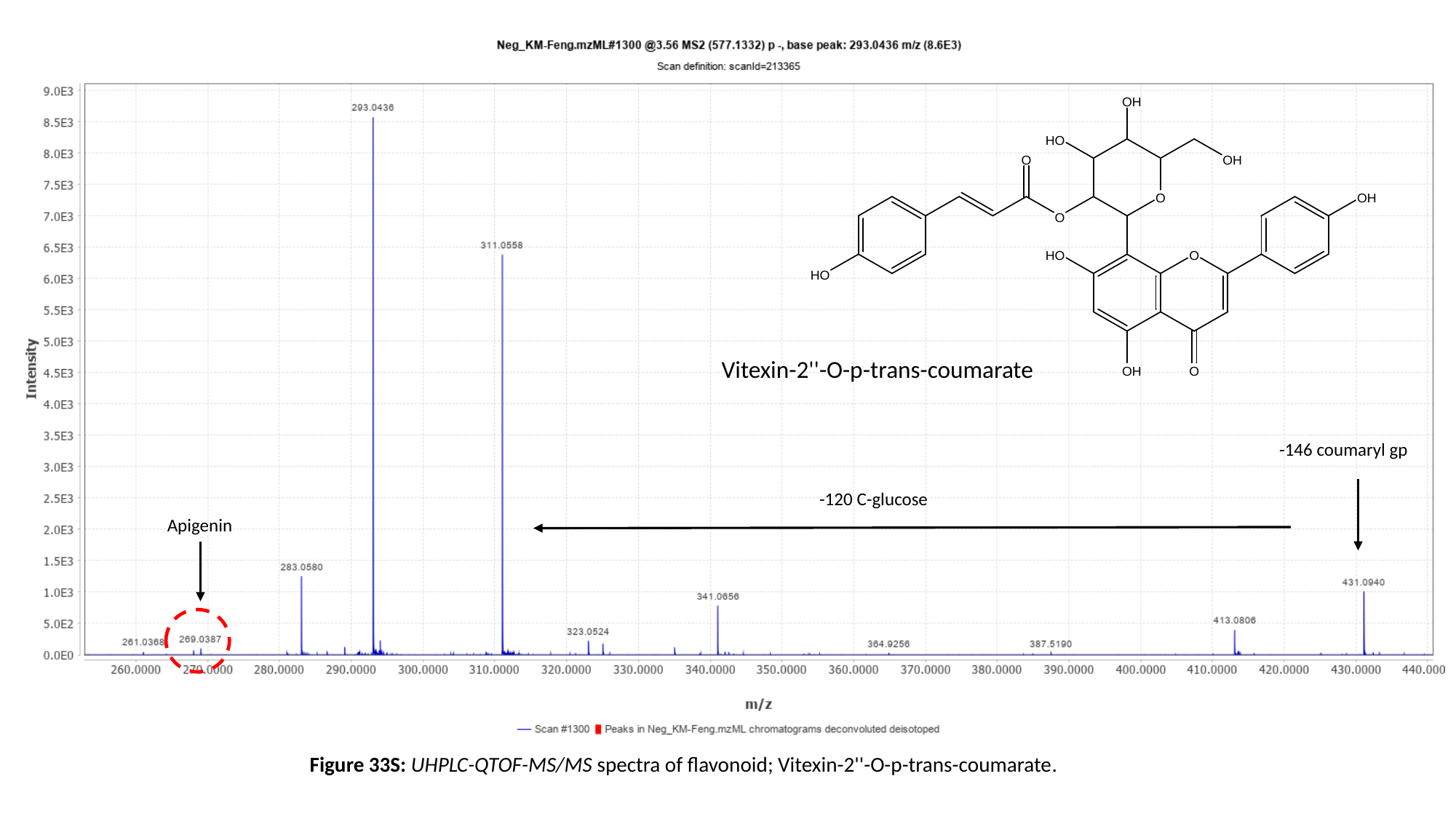

Vitexin-2''-O-p-trans-coumarate
-146 coumaryl gp
-120 C-glucose
Apigenin
Figure 33S: UHPLC-QTOF-MS/MS spectra of flavonoid; Vitexin-2''-O-p-trans-coumarate.

## Slide 34
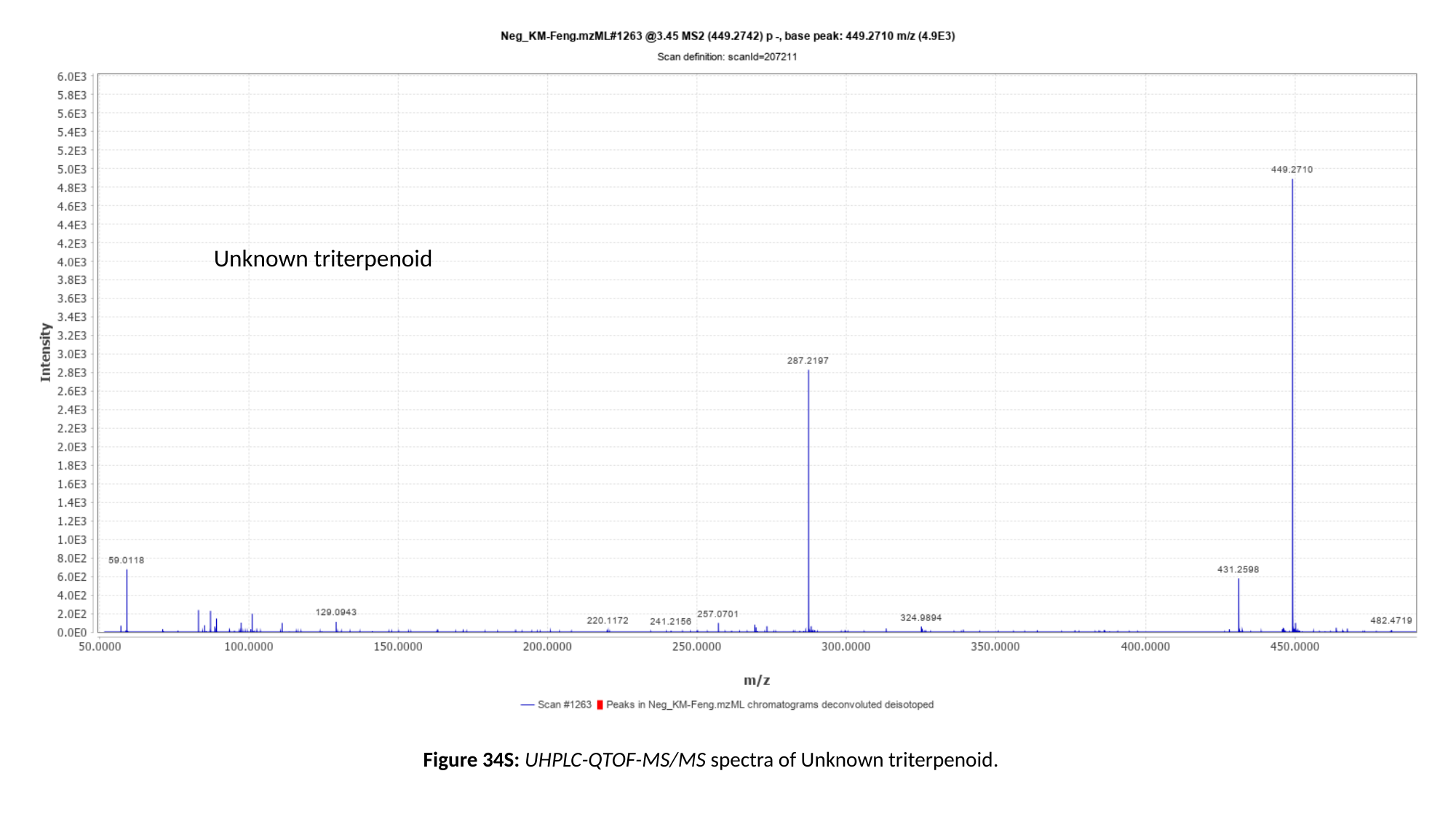

Unknown triterpenoid
Figure 34S: UHPLC-QTOF-MS/MS spectra of Unknown triterpenoid.

## Slide 35
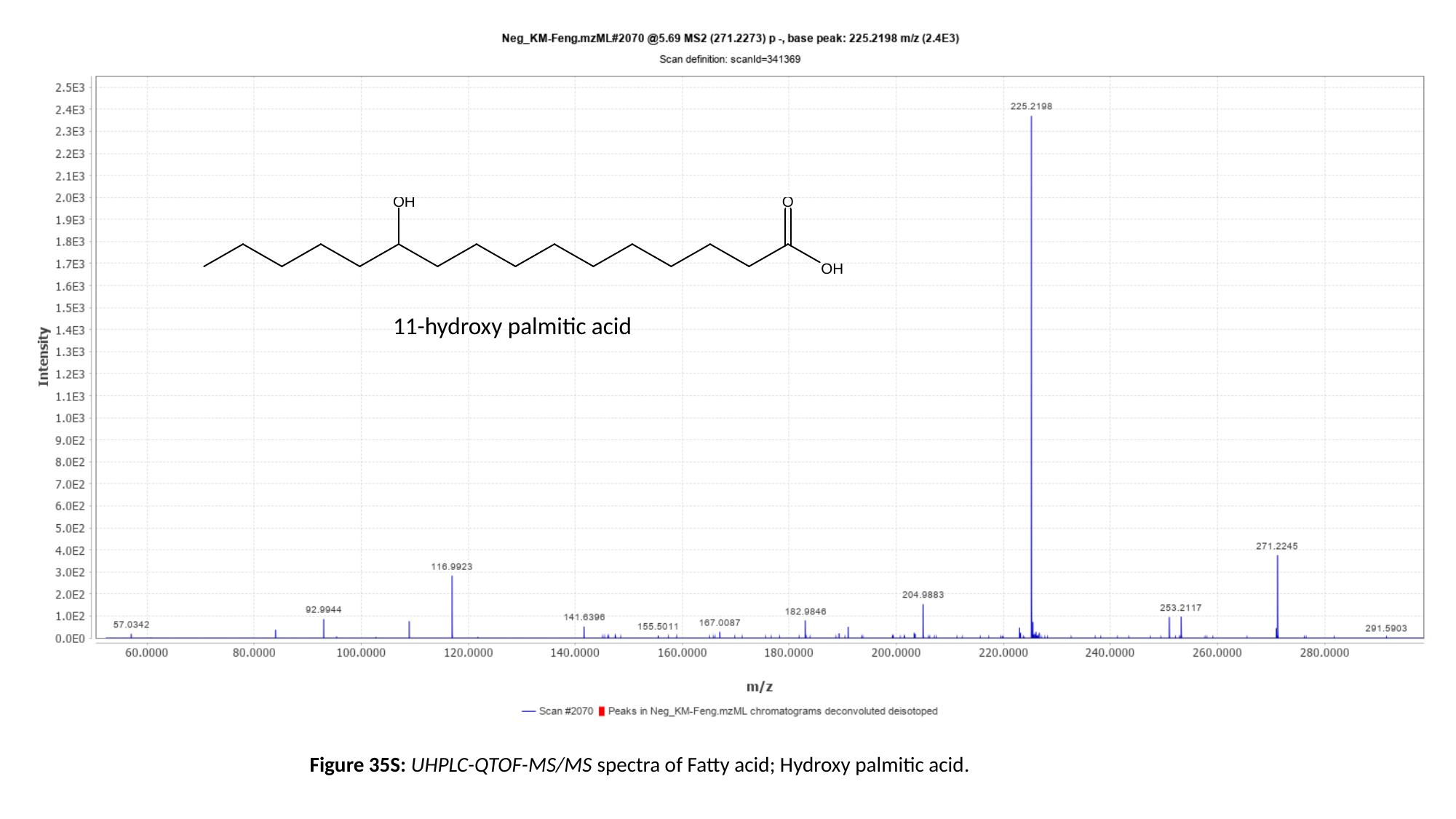

11-hydroxy palmitic acid
Figure 35S: UHPLC-QTOF-MS/MS spectra of Fatty acid; Hydroxy palmitic acid.
