# Supplementary material for: Fenugreek seed extract–doxorubicin synergy against hepatocellular carcinoma in HepG2 cells: in vitro and in silico mechanistic studies
Source: BMC Complement Med Ther. 2026 May 6;26:171. doi: 10.1186/s12906-026-05386-3 (PMC13151130; doi:10.1186/s12906-026-05386-3)
Supplement: Supplementary file 2 — Supplementary Material 2: Additional file 2 (.docx): Summary of Molecular docking analysis results of compounds from FAE with apoptosis (Bcl-2) and autophagy (LC3) targets, including docking scores and amino acid interactions [file 12906_2026_5386_MOESM2_ESM.docx]

**Additional File (3).** Full-length, unprocessed Western blot images corresponding to the cropped blot panels presented in the main manuscript figures.

**General Information.** All Western blot experiments were performed on HepG2 cell lysates. Proteins were detected using enhanced chemiluminescence (ECL) and imaged using a Bio-Rad ChemiDoc system. The original, uncropped blot images shown below were obtained directly from the service report provided by Nawah Scientific. No digital enhancement or background subtraction was applied to the main figures before cropping.

**Full-length Bax and Bcl-2 blots.** These blots correspond to the cropped panels shown in **Figure 6** of the main manuscript. The membrane shows full-length Bax (~34 kDa) and Bcl-2 (~26 kDa) bands with all lane edges visible. GAPDH was used as the loading control for normalization.

| **Bax** | **Bcl-2** |
| --- | --- |
| 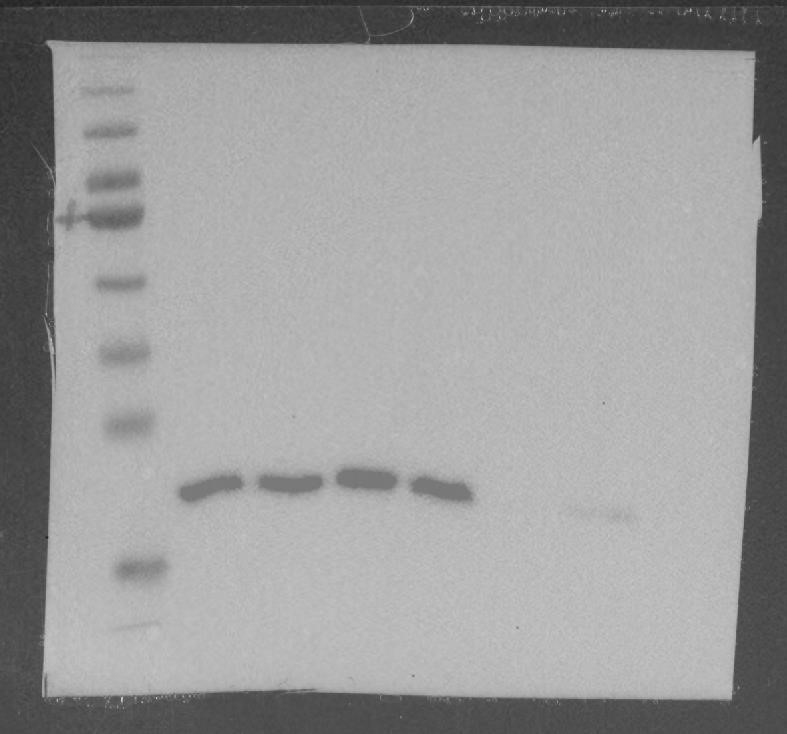 | 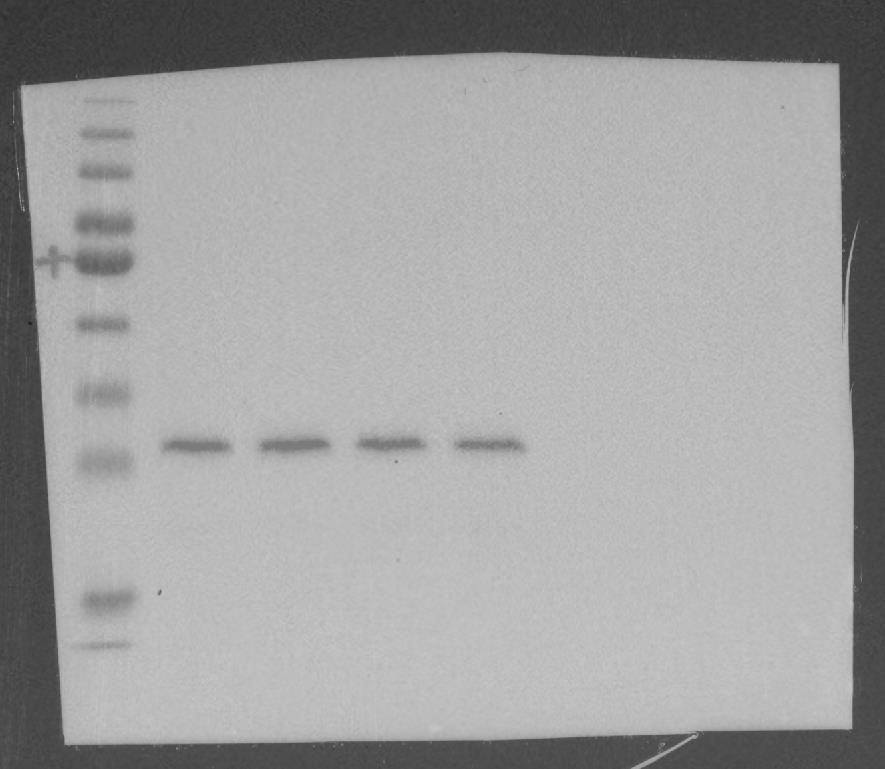 |

**Figure S1.** Uncropped blot corresponding to Figure 6D.

**Full-length LC3 and GAPDH blots.** These blots correspond to the cropped panels shown in **Figure 7** of the main manuscript. The full membrane displays LC3 (~11 and 17 kDa) and GAPDH (~34–36 kDa) bands across all treatment conditions. All blot edges are retained, and no lanes were removed or rearranged.

| **LC3** | **GAPDH** |
| --- | --- |
| 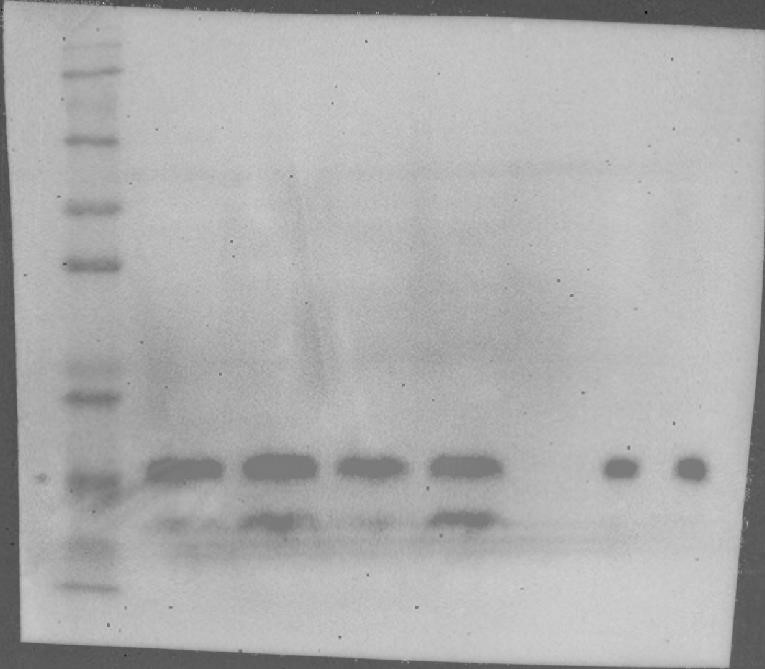 | 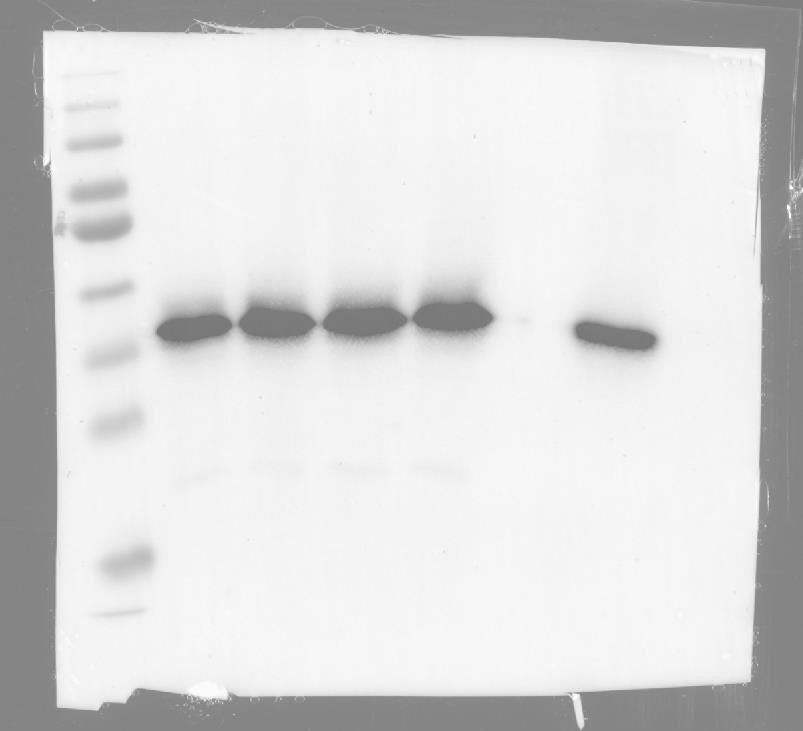 |

**Figure S2.** Uncropped blot corresponding to Figure 7C.

**Lane and Treatment Description (All Blots).**

Lane M: Prestained protein ladder (Thermo Scientific PageRuler Prestained NIR).
Lane 1: Control (untreated HepG2 cells).
Lane 2: Fenugreek aqueous extract (FAE).
Lane 3: Doxorubicin (DOX).
Lane 4: Combination (FAE + DOX).
+Ve Control: Positive control (where applicable).

**Western blot images.**

| **Name** | **Band** |
| --- | --- |
|  | **M 1 2 3 4 +Ve Control** |
| **Bax** | **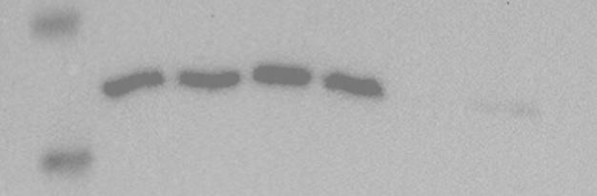**  26 kDa  15 kDa |
| **Bcl-2** | **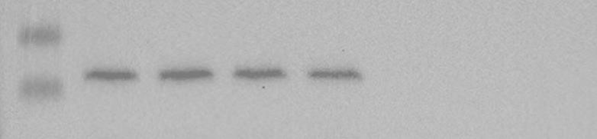**  34 kDa  26 kDa |
| **LC3** | **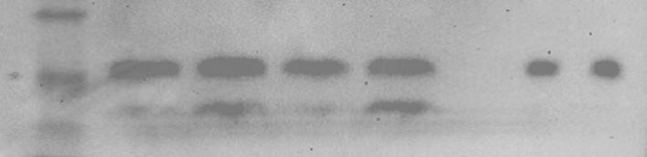**  20 kDa  11 kDa  17 kDa |
| **GABDH** | **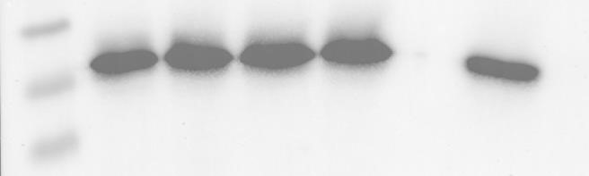**  43 kDa  34 kDa  26 kDa |

**Immunoblot band intensity**

| **Sample ID** | **Band intensity** | | **Normalization Bax**  **To**  **GAPDH** | **Normalization**  **Bax**  **To**  **Control** |
| --- | --- | --- | --- | --- |
|  | **GAPDH** | **Bax** |  |  |
| **Control** | 23367862 | 203456 | 0.008707 | 1 |
| **Fenugreek** | 27073980 | 205135 | 0.007577 | 0.870234185 |
| **Doxorubicin** | 27326628 | 238640 | 0.008733 | 1.003010947 |
| **Combination** | 26490516 | 212394 | 0.008018 | 0.920874196 |
| **Sample ID** | **Band intensity** | | **Normalization Bcl-2**  **To**  **GAPDH** | **Normalization**  **Bcl-2**  **To**  **Control** |
|  | **GAPDH** | **Bcl-2** |  |  |
| **Control** | 23367862 | 126720 | 0.005423 | 1 |
| **Fenugreek** | 27073980 | 145035 | 0.005357 | 0.987858021 |
| **Doxorubicin** | 27326628 | 126360 | 0.004624 | 0.852702208 |
| **Combination** | 26490516 | 100305 | 0.003786 | 0.698242017 |
| **Sample ID** | **Band intensity** | | **Normalization**  **LC3A/B-I**  **To**  **GAPDH** | **Normalization**  **LC3A/B-I To Control** |
|  | **GAPDH** | **LC3A/B-I**  **(Lower bands)** |  |  |
| **Control** | 23367862 | 56071 | 0.002399 | 1 |
| **Fenugreek** | 27073980 | 126175 | 0.00466 | 1.942235 |
| **Doxorubicin** | 27326628 | 48265 | 0.001766 | 0.736083 |
| **Combination** | 26490516 | 156085 | 0.005892 | 2.455565 |
| **Sample ID** | **Band intensity** | | **Normalization**  **LC3A/B-II**  **To**  **GAPDH** | **Normalization**  **LC3A/B-II**  **To**  **Control** |
|  | **GAPDH** | **LC3A/B-II**  **(Upper bands)** |  |  |
| **Control** | 23367862 | 299328 | 0.012809 | 1 |
| **Fenugreek** | 27073980 | 418166 | 0.015445 | 1.20578 |
| **Doxorubicin** | 27326628 | 250792 | 0.009178 | 0.716472 |
| **Combination** | 26490516 | 304934 | 0.011511 | 0.898643 |

**Replicates.** The blots shown represent one experimental replicate selected for presentation in the main figures.

**Source of Raw Data.** All original blot images and densitometric analyses were generated by Nawah Scientific.
