# Supplementary material for: Fenugreek seed extract–doxorubicin synergy against hepatocellular carcinoma in HepG2 cells: in vitro and in silico mechanistic studies
Source: BMC Complement Med Ther. 2026 May 6;26:171. doi: 10.1186/s12906-026-05386-3 (PMC13151130; doi:10.1186/s12906-026-05386-3)
Supplement: Supplementary file 3 — Supplementary Material 3: Additional file 3 (.docx): "Uncropped, full-length original Western blot images corresponding to all cropped panels shown in Figures 6 and 7 [file 12906_2026_5386_MOESM3_ESM.docx]

**Additional file 2:** Summary of Molecular docking analysis results of compounds from FAE with apoptosis (Bcl-2) and autophagy (LC3) targets, including docking scores and amino acid interactions.

**Experimental Method.**

Docking studies were carried out using Molecular Operating Environment (MOE 2020.0901, Chemical Computing Group, Montreal, Canada) as the computational software at the Department of [Medicinal Chemistry](https://www.sciencedirect.com/topics/pharmacology-toxicology-and-pharmaceutical-science/pharmaceutical-chemistry), Faculty of Pharmacy, Assiut University, Assiut/Egypt. All molecular modeling studies were carried out on an Intel(R) Xeon(R) CPU E5-1650 3.20 GHz processor with 16 GB of memory and the Windows 11 Professional operating system. The X-ray crystallographic structures of apoptosis regulator Bcl-2 and LC3A (PDB: ID: 4MAN and 6TBE, respectively) complexed with their native substrates (Navitoclax Analog and novobiocin, respectively) were downloaded from the RCSB protein data bank [(www.rcsb.org).](https://www.rcsb.org/) The X-ray crystallographic structure of the target protein was prepared, and all water molecules were removed. The studied compounds were built using the MOE software's builder interface and subjected to conformational search. Conformers were subjected to energy minimization until an RMSD gradient of 0.0001 kcal/mol and an [RMS](https://www.sciencedirect.com/topics/pharmacology-toxicology-and-pharmaceutical-science/rhabdomyosarcoma) distance of 0.1 Å with the Amber14: EHT force-field, and the partial charges were automatically calculated. The obtained database was then saved as an MDB file for use in the docking calculations.

Affinity dG was used as the scoring function, and it estimates the free energy of binding of the ligand from a given pose. Alpha PMI was used as a placement method.

The docking protocol is initially validated by redocking the co-crystalized ligands into the active sites of both proteins (Bcl-2 and LC3) to assign binding modes and root-mean-square deviation (RMSD) as docking parameters. The results disclosed the redocked pose overlayed with the co-crystalized ligands. The rmsd values were less than 2 and showed identical interactions as in Figures 1S and 2S.

Docking of the energy-minimized conformations was performed using the MOE Dock Wizard. The vicinity of the co-crystallized ligand was specified to be the docking site. Conformers of each tested compounds were checked for interactions with active-site residues and for docking scores relative to rmsd values, and compared with those of co-crystalized ligands.


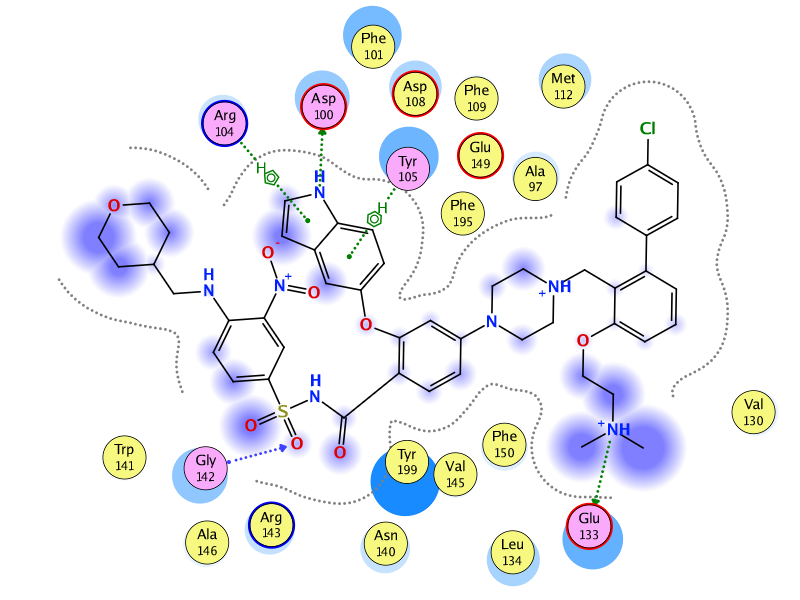


**Figure 1S.** 2D display of co-crystalized ligand (**Navitoclax** Analog) with Bcl-2 (pdb:4MAN).


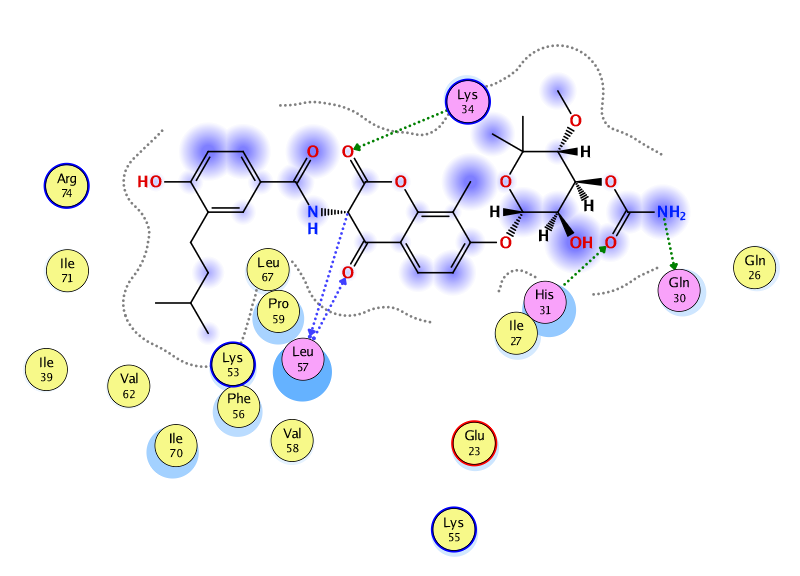


**Figure 2S.** 2D display of co-crystalized ligand (Novobiocin) in LC3 (pdb: 6TBE).


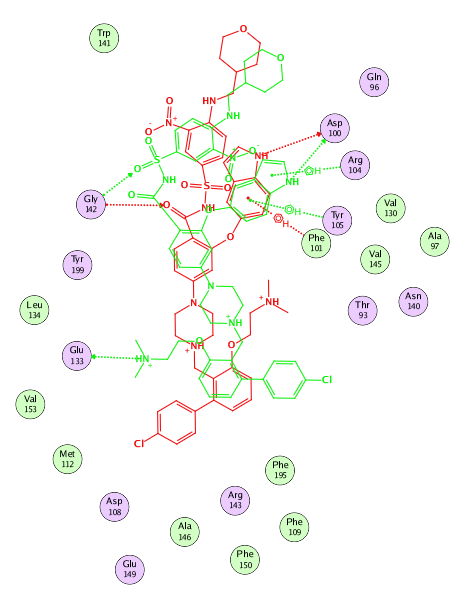

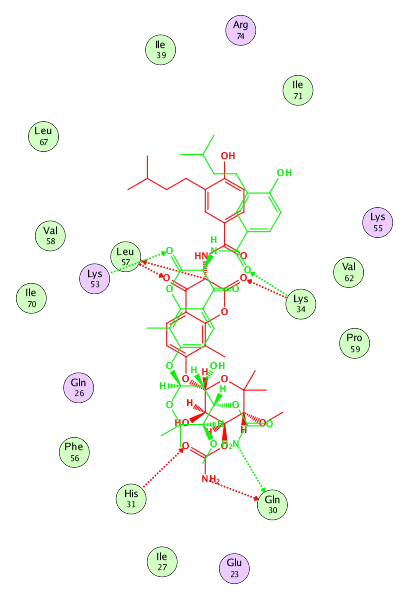


**(A)**

**(B)**

**Figure 3S. (A)** Overlay of co-crystalized ligand (green), and redocked ligand (red) into the active site of Bcl-2 (PDB ID: 4MAN), **(B)** Overlay of co-crystalized ligand (green), and redocked ligand (red) into the active site of LC3 (PDB ID: 6TBE).

**Table (1S)**: Summary of Molecular docking analysis of remaining compounds with Bcl-2 and LC3 proteins.*

|  |  | **Bcl-2** | | **LC3** | |
| --- | --- | --- | --- | --- | --- |
|  | **Compounds** | **Docking Score** | **Interactions residues (Distance)** | **Docking Score** | **Interactions residues (Distance)** |
| **1** | 2-hydroxy-3-O-[β-D-glucopyranosyl]tigogenin | -9.33 | ASP 100 (2.83)  ARG 104 (3.53) | -8.5258 | LEU 57 (2.91)  LYS 53 (2.93)  LYS 53 (3.39)  ARG 74 (3.09)  GLN 26 (2.95) |
| **2** | Isoorientin | -8.11 | ASP 100 (3.17)  ARG 143 (2.97) | -8.8323 | GLU 23 (2.88)  GLN 30 (2.89)  LYS 53 (3.25)  LEU 57 (3.62) |
| **3** | Isoviolanthin | -9.44 | ASP 108 (2.82)  GLU 133 (2.85)  ARG 143 (2.87) | -8.7801 | GLN 30 (3.02)  HIS 31 (3.16)  LEU 57 (3.03)  4LYS 53 (3.31)  L5YS 34 (3.02) |
| **4** | Isovitexin | -9.56 | ASP 100 (2.77)  ARG 104 (3.27) | -8.303 | HIS 31 (2.93)  LYS 53 (3.01)  LYS 34 (3.15)  LYS 34 (2.79) |
| **5** | Orientin | -8.76 | ASP 108 (3.09)  TYR 105 (4.06)  LEU 134 (4.73) | -8.1582 | GLN 30 (2.98)  GLU 23 (3.34)  LYS 55 (3.17)  HIS 31 (3.27) |
| **6** | Orientin-2''-O-p-trans-coumarate | -9.61 | ASP 100 (2.96)  GLY 142 (4.45) | -9.003 | LEU 57 (2.99)  GLU 23 (3.23)  LEU 57 (3.09)  LYS 34 (2.96)  LYS 53 (2.90) |
| **7** | Soyasaponin I | -6.70 | ARG 104 (3.01)  ARG 104 (3.11)  ASP 100 (3.50) | -9.45 | LYS 53 (2.90) |
| **8** | Trigoneoside Xa | -9.57 | ASP 100 (2.90)  ASP 108 (3.04) | -9.674 | GLN 30 (3.03)  LYS 53 (3.04)  LYS 55 (2.93) |
| **9** | Trillin; diosgenyl-β-D-glucopyranoside | -8.51 | GLU 133 (3.09)  ARG 136 (3.18) | -9.13 | LEU 57 (3.01)  LYS 34 (2.99)  LYS 53 (3.04) |
| **10** | Vicenin-II | -9.31 | ASP 137 (3.30)  GLU 133 (2.72)  PHE 101 (3.99) | -8.602 | LEU 57 (2.86)  LYS 34 (3.97)  LYS 34 (3.96)  ARG 74 (2.85) |
| **11** | Vicenin-I | -8.71 | ASP 100 (3.09)  ASN 140 (2.97)  GLY 142 (4.28) | -8.804 | GLN 30 (3.09)  HIS 31 (3.14)  LYS 53 (3.01)  LYS 34 (2.89) |
| **12** | Violanthin | -9.23 | GLU 133 (2.85)  ASP 137 (2.91)  GLU 133 (3.05) | -8.82 | LEU 57 (2.99)  LYS 55 (2.93) |
| **13** | Vitexin | -8.32 | ASP 100 (2.78)  ARG 104 (3.28) | -8.005 | HIS 31 (2.93)  LEU 57 (3.06)  LYS 34 (2.87)  LYS 34 (3.64) |
| **14** | Vitexin-2''-O-p-trans-coumarate | -7.61 | ASP 100 (3.25)  TYR 105 (3.96)  TYR 199 (3.70) | -8.747 | GLU 23 (3.16)  LEU 57 (3.09)  LYS 34 (2.95)  LYS 53 (2.91) |

*Docking score measured in (kcal/mol) and distances in Å.
